# The food contaminant acetamide is not an *in vivo* clastogen, aneugen, or mutagen in rodent hematopoietic tissue

**DOI:** 10.1016/j.yrtph.2019.104451

**Published:** 2019-11

**Authors:** Martha M. Moore, Bhaskar Gollapudi, Rajendra Nagane, Nadeem Khan, Manish Patel, Tushar Khanvilkar, Avani M. Roy, E. Ramesh, Bryan Bals, Farzaneh Teymouri, Rance Nault, Venkataraman Bringi

**Affiliations:** a Ramboll, 23 Alban Lane, Little Rock, AR, 72223, USA; b Exponent, 1800 Diagonal Rd Suite 500, Alexandria, VA, 22314, USA; c Jai Research Foundation India, NH-8 Near Daman Ganga Bridge Valvada, Vapi, Gujarat, 396 105, India; d Eurofins Advinus Limited, Peenya II Phase, Bangalore, 560 058, India; e MBI International, 3815 Technology Blvd, Lansing, MI, 48910, USA; f Department of Biochemistry and Molecular Biology, Institute for Integrative Toxicology, Michigan State University, 1129 Farm Lane Rm 248, East Lansing, MI, 48824, USA; g Department of Chemical Engineering and Materials Science, Michigan State University, 428 S Shaw Lane Rm 2100, East Lansing, MI, 48824, USA

**Keywords:** Genotoxicity, *In vivo* micronucleus test, Mutagenicity, *In vivoPig-A* gene mutation assay

## Abstract

Acetamide (CAS 60-35-5) is classified by IARC as a Group 2B, possible human carcinogen, based on the induction of hepatocellular carcinomas in rats following chronic exposure to high doses. Recently, acetamide was found to be present in a variety of human foods, warranting further investigation. The regulatory body JECFA has previously noted conflicting reports on acetamide's ability to induce micronuclei (MN) in mice *in vivo*. To better understand the potential *in vivo* genotoxicity of acetamide, we performed acute MN studies in rats and mice, and a subchronic study in rats, the target species for liver cancer. In the acute exposure, animals were gavaged with water vehicle control, 250, 1000, or 2000 mg/kg acetamide, or the positive control (1 mg/kg mitomycin C). In the subchronic assay, bone marrow of rats gavaged at 1000 mg/kg/day (limit dose) for 28 days was evaluated. Both acute and subchronic exposures showed no change in the ratio of polychromatic to total erythrocytes (P/E) at any dose, nor was there any increase in the incidence of micronucleated polychromatic erythrocytes (MN-PCE). Potential mutagenicity of acetamide was evaluated in male rats gavaged with vehicle control or 1500 mg/kg/day acetamide using the *in vivoPig-a* gene mutation assay. There was no increase in mutant red blood cells or reticulocytes in acetamide-treated animals. In both acute and sub-chronic studies, elevated blood plasma acetamide in treated animals provided evidence of systemic exposure. We conclude based on this study that acetamide is not clastogenic, aneugenic, or mutagenic *in vivo* in rodent hematopoietic tissue warranting a formal regulatory re-evaluation.

## Highlights

• *In vivo* micronucleus tests with acetamide in mice and rats.
• Acetamide blood plasma levels demonstrated evidence of exposure.
• Acetamide does not induce micronuclei in rats and mice.
• Acetamide does not increase mutations in the rat *Pig-a* gene mutation assay.

## Abbreviations

IARC: International Agency for Research on Cancer
JECFA: Joint FAO/WHO Expert Committee on Food Additives
OECD: Organization for Economic Co-operation and Development
MN: Micronucleus
MN-PCE: Micronucleated polychromatic erythrocytes
P/E: Ratio of polychromatic to total erythrocytes
JRF: Jai Research Foundation

## 1 Introduction

Acetamide (CAS 60-35-5), a simple hydrophilic amide with the formula CH_3_CONH_2_, has been classified by the International Agency for Research on Cancer (IARC) as a Group 2B possible human carcinogen (IARC, 1999). Chronic studies in rats conducted by the US National Cancer Institute tested daily acetamide exposure at a single high dose of 1000 mg/kg/day, administered via diet, and reported neoplastic nodules and carcinomas of the liver in 1/47 and 41/47 acetamide-treated male rats and 3/48 and 33/48 female rats, respectively, while none of the control rats developed liver tumors (Fleischman et al., 1980). These findings are consistent with other reports of high doses of acetamide in diet causing liver cancer in rats (Flaks et al., 1983; Weisburger et al., 1969). In mice, two dietary dose levels (~1770 and ~3540 mg/kg/day) were tested and an increase in the incidence of malignant lymphomas was observed (controls, 0/95; low-dose, 7/50; high-dose, 7/46; p = 0.004, Cochran-Armitage test for trend), but females showed a reverse dose-response relationship for malignant lymphomas, with 10/92 in controls, 3/41 at the low dose, 2/46 at the high dose (Fleischman et al., 1980).

Acetamide has been investigated as a residue from some pesticides (WHO, 2005), as a solvent with unique properties (Zhang et al., 2012), as an impurity in the manufacture of pharmaceuticals (Snodin, 2011), and as a metabolite of drugs such as phenacetin (Hinson, 1983), metronidazole (Koch et al., 1979) and acetohydroxamic acid (Putcha et al., 1984). However, more recent findings show that acetamide is present throughout the universe from intra-stellar clouds (Hollis et al., 2006) to common foods (Vismeh et al., 2018). Earlier reports indicating that acetamide was present in milk, eggs, and meat have been recently confirmed by Vismeh et al. (2018) who surveyed milk and beef samples from different states in the US. Wei, 2016 reported the appearance of acetamide in the NMR spectra of roasted chicory roots which was also confirmed by Vismeh et al. (2018) who reported the presence of acetamide in roasted coffee beans and instant coffee but not raw coffee beans. Acetamide is also formed as a by-product during ammoniation of forages for ruminants (Chundawat et al., 2010) and widespread adoption of ammoniation technology for feed production could result in acetamide being present as a feed contaminant. These recent findings suggest that acetamide should be considered as a food and feed contaminant, that human exposure to acetamide is higher than previously recognized, and, is thus, a relevant public health topic.

In 2005, the FAO/WHO Joint Expert Committee on Food Additives (JECFA) considered acetamide as a food flavoring agent, and indicated that the possibility of a genotoxic mechanism for the induction of cancer by acetamide could not be discounted (Abbott et al., 2006). This JECFA concern was based on potential clastogenic/aneugenic activity reported in an *in vivo* mouse bone marrow micronucleus (MN) test published by Chieli et al. (1987) and cited by JECFA (Abbott et al., 2006). It should be noted that this study contained multiple deficiencies compared to the recommendations of the current OECD Test Guideline (TG 474) for *in vivo* MN assays. While the acetamide used in this study was purchased from Aldrich Co. (USA), the authors recrystallized it before use and do not provide any information on the purity of the test material before or after recrystallization. In the study, female C57BL/6 mice (6–8 weeks old) were treated by gavage with acetamide dissolved in water. A single dose (200 mg/kg) using only 3 mice was investigated in the publication. Two sampling times (6 and 30 h) after treatment were used and a total of 3000 cells were scored from the 3 treated mice and a total of 5000 cells scored from 5 untreated control mice. The negative controls were reported as having 3.6% micronucleated polychromatic erythrocytes (MN-PCE) (range of 3–5%) and the acetamide treated animals were reported to have 19.3% MN-PCE (with a range of 17–21%). Thus, the number of dose levels, the number of mice per dose group, and the number of cells scored per animal were insufficient per current standards. Furthermore, there was no measure of toxicity in the treated animals and the sampling times post treatment do not meet the current recommendations. Most importantly, the incidence of 3.6% MN-PCE in the control is an order of magnitude higher than the values reported in various strains of mice (Salamone and Mavournin, 1994) and raises significant concern as to the adequacy of the methodology used in identifying MN-PCE and the laboratory quality control procedures.

Later attempts by several groups to reproduce the Chieli et al. (1987) result have all proven to be unsuccessful, and acetamide was found to be consistently negative in other published *in vivo* mouse MN tests (De Boeck et al., 2005; Mirkova, 1996; Miura et al., 1994; Morita et al., 1997). Furthermore, these tests were performed in mice which, unlike rats, do not develop liver tumors following high dose acetamide exposure (Fleischman et al., 1980) warranting additional investigation in both sexes and species which are guided by GLP and OECD recommendations.

A conclusive evaluation of acetamide's ability to induce MN using the current guidelines is an important step as a part of understanding potential modes of action for its carcinogenicity and in assessing its health risk as a food contaminant. It has been demonstrated to be non-mutagenic in bacterial reverse mutation tests by many investigators (Claxton et al., 1988; Dybing et al., 1987; Emmert et al., 2006; Miura et al., 1994), including a 2014 European Chemicals Agency submission conducted under Organization for Economic Co-operation and Development (OECD) guidelines (TG 471) and GLP standards (ECHA, 2018). Conversely, acetamide has been reported to be positive in a yeast recombination assay (Schiestl, 1989) and in Drosophila (Batise-Alentorn et al.; Monoz and Mazar-Barnett). However, these test systems do not necessarily provide information a as material's ability to induce genetic damage in mammals. Furthermore, the OECD test guideline for Drosophila was deleted in 2014, indicating that this test is no longer a part of the OECD recommended tests (OECD, 2017).

Sasaki et al. (2000) reported a positive finding for acetamide in stomach, colon, lung/respiratory tract and bone marrow, as a part of a large investigation of multiple chemicals, in the comet assay; the result was obtained at a single dose of 5000 mg/kg, administered intraperitoneally. This study was not consistent with the new OECD TG for the comet assay (TG 489) which recommends a limit dose of 2000 mg/kg for administration periods of less than 14 days and that the exposure route be consistent with that for possible human exposure. Specifically, the revised OECD test guidelines for *in vivo* genotoxicity assays, list intraperitoneal exposure as generally not recommended as a relevant route for assessing potential human risk. Hence, this test result should not be interpreted as providing evidence that acetamide is genotoxic *in vivo*. Furthermore, the comet assay, which only measures primary DNA damage, does not provide definitive evidence that a test substance can induce mutations *in vivo*.

Given the expression of concern by JECFA regarding acetamide genotoxicity based on a single positive *in vivo* mouse MN study, the absence of an *in vivo* rat MN study, and in anticipation of public interest in understanding the health risks of acetamide in foods, we conducted new studies in male and female mice and rats guided by the latest OECD 474 test guidelines to resolve the uncertainty regarding acetamide's ability to induce MN-PCE *in vivo*. We chose to conduct the study in both mice and rats to provide a definitive evaluation in mice and to provide new information for the rat which was the only species to demonstrate liver tumors in the long-term studies (Flaks et al., 1983; Fleischman et al., 1980; Weisburger et al., 1969). We evaluated the induction of MN-PCE in both an acute exposure and a subchronic exposure study. Additionally, to provide information as to whether acetamide is mutagenic in rodents, we conducted the *in vivo* *Pig-a* gene mutation assay. We note that the subchronic MN-PCE and *Pig-a* studies were a part of a larger transcriptomics 28-day study focused on the liver that will be published separately (Nault et al., 2019); in this study the gene expression results did not indicate that genotoxicity was induced in the liver by the acetamide exposure.

## 2 Materials and methods

### 2.1 Chemicals

Acetamide (CAS No. 60-35-5) was purchased from Tokyo Chemical Industry Co. Ltd. and had an assigned purity of 99.2%. Mitomycin C (CAS No. 50-07-7) was purchased from Sigma Chemical Corporation. Chemicals used for analytical chemistry, acetamide-d_3_ (acetamide-2.2.2-d_3_, CAS No. 23724-06-9) was purchased from Clearsynth Labs Ltd. and 9-xanthrydrol (CAS No. 90-46-0) was purchased from Sigma Chemical corporation. Acetamide and mitomycin C were dissolved in distilled water prior to administration. Acetamide solutions were used within 2 h of preparation. Acetamide is shown to be stable for at least 23 h at room temperature (see study report G16085; http://dataverse.harvard.edu/dataverse/acetamide).

### 2.2 Animals

*In vivo* assays consisted of three independent studies evaluating acute or subchronic acetamide exposure. For all studies, animals were randomly assigned to treatment groups, acclimatized to laboratory conditions for at least 5 days, and observed at least once daily for clinical symptoms. Animals were housed with no more than 4 mice per cage and no more than 3 rats per cage. Animals were randomly assigned to various treatment groups based on body weights; body weight variation was within ±20% of the mean body weight for each sex of each species. Animals were fed *ad libitum* with standard rodent pellet feed (Teklad Certified Global 16% Protein Rodent Diet) and purified water. Environmental conditions were maintained at 22 ± 3° C, relative humidity at between 30% and 70%, photoperiod of 12 h light and darkness, and air exchanges at a 12–15 vol per hour. Parameters for these studies were selected based on the updated 2016 OECD guideline for *in vivo* MN tests in rodents (OCED TG 474). All experiments were conducted in compliance with the OECD Principles of Good Laboratory Practice (GLP), and all plans and data were verified by an internal QA department at the Jai Research Foundation (JRF; Vapi, Gujarat, India) for acute exposures, or Eurofins Advinus Limited (Bengaluru, Karnataka, India) for subchronic exposures. Experiments were conducted with the approval of Institutional Animal Ethics Committees at JRF and Eurofins Advinus Ltd. which are both accredited by the Association for Assessment and Accreditation of Laboratory Animal Care (AAALAC). Detailed study reports were deposited onto the Harvard Dataverse (http://dataverse.harvard.edu/dataverse/acetamide).

### 2.3 Dose selection

As acetamide was not anticipated to be toxic, acute exposures consisted of a high dose of 2000 mg/kg/day based on the maximum limit dose recommended in OECD guideline TG 474. The medium dose of 1000 mg/kg/day was selected based on the estimated exposure levels used in the long-term rodent carcinogenicity studies. The low dose of 250 mg/kg/day was chosen to approximate the 200 mg/kg/day dose at which Chieli et al. (1987) reported their positive effect. Consistent with OECD guidelines, the range between doses were no more than 4-fold apart. The subchronic MN assessment was performed at a single (limit) dose of 1000 mg/kg/day in male and female rats treated for 28 days based on OECD guideline TG 474 for evaluations lasting longer than 14 days. As the *Pig-a* gene mutation does not have an OECD guideline, a higher dose of 1500 mg/kg/day, for the 28 day treatment was selected in male rats, the more sensitive sex for cancer development based on chronic studies.

### 2.4 Acute micronucleus assay

Healthy male and female Hsd:ICR(CD1) mice aged 8–10 weeks and Wistar (RccHan:WIST) rats aged 9–10 weeks were obtained from the JRF Animal Breeding Facility. Female animals were nulliparous and non-pregnant. For each species, five treatment groups were evaluated including negative (vehicle control), 250, 1000, and 2000 mg/kg acetamide treatment groups, and positive controls receiving 1 mg/kg dose of mitomycin C. A total of 6 animals/sex/group were evaluated. Acetamide was administered in 10 mL/kg body weight by oral intubation for two consecutive days, 24 ± 1 h apart. Mytomycin C was administered by a single intraperitoneal injection. Body weight and clinical signs were recorded prior to dosing on each day and before sacrifice by CO_2_ asphyxiation 22–23 h (mice) or 23–24 h (rats) following the last treatment. Femoral bones from each animal were excised and the epicondyle tips were removed. Bone marrow was expelled by flushing with fetal bovine serum and the aspirated cell contents were dissociated by mixing and centrifugation. A smear was prepared, fixed with methanol, and stained with 5% Giemsa. Slides were coded and then manually counted under a light microscope. The proportion of immature erythrocytes among the total erythrocytes (immature + mature), i.e., P/E ratio was determined for each animal by counting a minimum of 500 erythrocytes. A minimum of 4500 polychromatic immature erythrocytes, per animal, was scored for the incidence of MN-PCE.

### 2.5 Subchronic micronucleus assay

Samples for the subchronic MN assay were obtained from an independent study designed to evaluate dose-dependent transcriptomic effects of subchronic acetamide exposure. In short, healthy male and female Wistar rats (Crl:WI) aged 8–10 weeks were obtained from Charles Rivers Laboratories (Wilmington, MA, USA). Female animals were nulliparous and non-pregnant. Five animals/sex/group were evaluated for water vehicle control and 1000 mg/kg/day treatment groups. Acetamide was administered in aliquots of 10 mL/kg body weight by oral intubation daily, 24 ± 3 h apart. Rats were examined for clinical signs at least twice daily. On day 29 (24 h following the final dose), the right femoral bone from each animal was excised and the femur head removed. Bone marrow was flushed with fetal bovine serum, and the contents were centrifuged. Approximately 10 μL of the cell suspension was spread evenly on a glass slide, dried, and stained with acridine orange. Slides were manually counted by fluorescence microscopy. A minimum of 500 erythrocytes and 4000 polychromatic immature erythrocytes were scored per animal for P/E ratio and MN-PCE, respectively. Strain-specific laboratory historical control data prepared from 5 Wistar rats/sex previously treated in a separate study with cyclophosphamide monohydrate (15 mg/kg; single intraperitoneal injection; 18–24 h following a single dose) were used for positive control. The mean %MN PCE (±SD) was 0.81 (0.19) and 0.88 (0.15) for males and females, respectively, and these means were significantly different (p < 0.01) compared to negative controls of the same species and sex.

### 2.6 *In vivoPig-a* gene mutation assay

Blood for the *Pig-a* gene mutation assay was collected from rats (6/group) gavaged with vehicle control or 1500 mg/kg/day acetamide in the same subchronic study used for the micronucleus assay. Blood was collected approximately 24 h after 28 consecutive days of dosing and stored according the MutaFlow Rodent Blood Freezing Kit manual (Litron laboratories, Rochester, NY, USA). Samples were sent to Litron Laboratories for processing and analysis. Briefly, frozen blood samples were thawed and the freezing solution washed out. Leukocytes were depleted using Lympholyte-Mammal (Cedarlane, Burlington, NC, USA) and leukodepleted samples labeled with antibodies for wild-type erythrocytes (anti-CD59-PE) and remaining platelets (anti-CD61-PE), washed, centrifuged, and re-suspended with anti-PE microbeads. A pre-column aliquot was added to nucleic acid dye (SYTO 13) and counting beads, then placed on ice until analysis. The remaining sample was added to a pre-rinsed LS column in a magnetic field which retains wild-type erythrocytes and platelets in the column and allows mutant cells to pass through. The post column mutant cell samples were resuspended in nucleic acid dye (SYTO 13) and counting beads. Pre- and post-column samples were analyzed on a BD FACSCalibur flow cytometer with CellQuest Pro software version 5.2 (Becton Dickinson, San Jose, CA, USA).

### 2.7 Evidence of exposure

Plasma samples were taken from each animal at sacrifice. Acetamide levels were measured in plasma using a validated analytical method described by Vismeh et al. (2018). Briefly, plasma samples were treated with a methanol/HCl solution at 40 °C for 120 min in the presence of excess 9-xanthydrol to derivatize acetamide, using propionamide as an internal standard. GC-MS analysis was conducted using an Agilent VF-5MS column at 30 m in length and a temperature of 250 °C. Quantitation was achieved by comparing peak area ratio for xanthyl acetamide (MW 239) with standard curves generated using derivatized product of acetamide-d_3_ (acetamide-2,2,2-d_3_) in plasma. Prior to use in the study, the analytical method was validated independently at JRF and Eurofins-Advinus. A Student's t-test was used to determine evidence of exposure for each individual group compared to their respective controls.

### 2.8 Criteria for data interpretation and statistical analyses

In the acute evaluation, percent MN in polychromatic erythrocytes (% MN-PCE) and P/E ratio were evaluated for normality using the Shapiro-Wilk's test. Normality tests for male and female treatment groups, for both species, were not significant, therefore a Bartlett test was performed to verify homogeneity of variance before conducting an ANOVA test followed by a Dunnett's *t*-test. Evaluation of % MN-PCE and P/E ratio for the positive control group in males was not significant using the Shapiro-Wilk's test for normality, which was subsequently assessed by the F-test for homogeneity of variance followed by a *t*-test. Shapiro-Wilk's test of normality was significant for female % MN-PCE, therefore a non-parametric Mann-Whitney test was performed. Statistical analyses were considered different at a significance level of 0.01 (*p* ≤ 0.01).

In the subchronic study, % MN-PCE underwent square root transformation and were tested for normality using the Shapiro-Wilk test, homogeneity of within group variances using Levene's test, and statistical differences determined by ANOVA. Statistical analyses were performed using Systat v12.0 (Systat Software Inc., San Jose, CA, USA) and data were considered different at a significance level of 0.05.

The data interpretation criteria for acute and subchronic studies were those recommended in OECD TG 474. A clear positive response included 1) a positive dose trend or a clear increase for a single dose/sampling time, 2) a statistically significant difference between the concurrent negative control and a treated group of animals, and 3) a requirement that one (or more) doses exceed the 95% confidence interval for the MN-PCE frequency observed in the historical negative control distribution. A clear negative response was defined as the absence of all three of these criteria.

For the *Pig-a* gene mutation assay, a value of 0.1 was added to the number of mutated reticulocytes to enable log transformation and significant changes (*p* ≤ 0.01) were determined by Student's t-test in R version 3.5.2. One outlier value in vehicle control animals was identified using Grubbs test for outliers, however, statistical significance was not impacted whether it was included or not.

## 3 Results and discussion

### 3.1 Micronucleus data

The experiments met the expected quality criteria in that there were no deviations from the protocols, no mortality or appearance of clinical symptoms, and both the negative and positive control values were consistent with laboratory historical distributions. As shown in Table 1, Table 2, Table 3, there was no decrease in the ratio of polychromatic to total erythrocytes (P/E) observed in either rats or mice at any dose or time-point tested indicating lack of bone marrow toxicity.

**Table 1:** Bone marrow MN data after acute (2 days) acetamide exposure in mice.

	Dose (mg/kg/day)	Animals Tested	MN-PCE counts	% MN-PCE (s.d.)	P/E Ratio (s.d.)	Total PCE Counted
Male	Distilled water	6	2,0,1,1,0,1	0.017 (0.015)	0.491 (0.024)	27063
	250	6	1,2,1,0,0,1	0.017 (0.015)	0.496 (0.025)	27060
	1000	6	0,1,0,0,1,2	0.013 (0.016)	0.498 (0.018)	27043
	2000	6	2,1,1,0,1,0	0.017 (0.015)	0.493 (0.024)	27086
	1 (Mitomycin-C)	6	47,40,49,82,65,72	1.308^1^ (0.363)	0.511 (0.039)	27127
Female	Distilled water	6	1,1,1,0,0,1	0.013 (0.010)	0.521 (0.025)	27077
	250	6	1,0,1,2,0,3	0.025 (0.027)	0.515 (0.022)	27041
	1000	6	0,2,1,0,2,1	0.020 (0.018)	0.522 (0.031)	27095
	2000	6	0,1,2,1,0,1	0.017 (0.015)	0.510 (0.025)	27054
	1 (Mitomycin-C)	6	65,43,57,54,48,96	1.340^1^ (0.422)	0.499 (0.018)	27066

^1^Denotes significant difference (p < 0.01) compared to species and sex matched negative control.

**Table 2:** Bone marrow MN data after acute (2 days) acetamide exposure in rats.

	Dose (mg/kg/day)	Animals Tested	MN-PCE counts	% MN-PCE (s.d.)	P/E Ratio (s.d.)	Total PCE Counted
Male	Distilled water	6	0,0,2,0,1,1	0.013 (0.016)	0.494 (0.031)	27072
	250	6	1,1,1,1,0,0	0.013 (0.010)	0.477 (0.018)	27060
	1000	6	2,1,1,2,0,0	0.020 (0.018)	0.498 (0.016)	27100
	2000	6	1,1,0,1,2,0	0.017 (0.015)	0.497 (0.027)	27097
	1 (Mitomycin-C)	6	43,51,46,39,35,38	0.9271^1^ (0.129)	0.514 (0.026)	27157
Female	Distilled water	6	1,2,0,1,1,0	0.017 (0.015)	0.495 (0.009)	27223
	250	6	1,2,1,2,0,0	0.020 (0.018)	0.497 (0.028)	27079
	1000	6	2,0,1,2,1,0	0.020 (0.018)	0.491 (0.020)	27055
	2000	6	0,1,1,1,0,1	0.013 (0.010)	0.508 (0.025)	27115
	1 (Mitomycin-C)	6	37,59,60,35,34,34	0.9571^1^ (0.283)	0.502 (0.028)	27054

^1^Denotes significant difference (p < 0.01) compared to species and sex matched negative control.

**Table 3:** Bone marrow MN data after subchronic (28 days) acetamide exposure in rats.

	Dose (mg/kg/day)	Animals Tested	MN-PCE counts	% MN-PCE (s.d.)	P/E Ratio (s.d.)	Total PCE Counted
Male	Distilled water	5	3,5,4,8,4	0.12 (0.05)	0.46 (0.01)	20487
	1000	5	5,8,5,5,4	0.13 (0.04)	0.46 (0.02)	20619
Female	Distilled water	5	5,4,4,3,4	0.10 (0.02)	0.47 (0.01)	20243
	1000	5	4,4,6,5,3	0.11 (0.03)	0.45 (0.02)	20269

The percentage of MN-PCE did not increase in either mice or rats acutely treated at up to 2000 mg/kg acetamide (Table 1, Table 2), or in rats following subchronic exposure (Table 3) at 1000 mg/kg/d. The concurrent positive control groups in both mice and rats yielded a statistically significant increase in the number of MN-PCE in comparison to the negative (vehicle) control group in the acute exposures and was consistent with the laboratory historical positive control data. For subchronic evaluations, the number and percentage of MN-PCE did not increase in the rats treated for 28 days at a dose of 1000 mg/kg/d. The positive control values, obtained from historical studies of the same laboratory, were significantly increased compared to the negative control group. Negative control values were consistent with laboratory historical values. Based on these data and the interpretation criteria we can conclude that acetamide does not induce MN-PCE in either mice or rats at acute (2 day) doses of 250, 1000, or 2000 mg/kg, or in rats following treatment with 1000 mg/kg/day for 28 days.

### 3.2 *In vivoPig-a* gene mutation data

The *in vivo* *Pig-a* gene mutation assay is a flow cytometry-based assay for mutations in the X-linked *Pig-a* (phosphatidylinositol glycan, class A) gene resulting in impaired production of glycosylphosphatidylinositol (GPI) cell surface anchors (Dobrovolsky, 2010; Gollapudi, 2015). The assay is noted to be both sensitive and specific, and is currently being evaluated by OECD for the development of new test guidelines for the assay. Acetamide exposure did not cause increases in the frequency of *Pig-a* gene mutations in red blood cells or reticulocytes (Table 4) supporting previous *in vitro* reports that acetamide is not a gene mutagen in the Ames test (Emmert et al., 2006). Lower average estimates for mutant RBCs or RETs, which were not statistically significant, are due to the outlier vehicle treated animal.

**Table 4:** *Pig-a* gene mutation assay after subchronic (28 days) acetamide exposure in rats.

	Dose (mg/kg/day)	Animals Tested	Mutant RBCs per 10^6^ total RBCs (s.d)	Mutant RETs per 10^6^ total RETs (s.d)
Male	Distilled water	6	2.6 (5.4)	7.1 (14.8)
	1500	6	0.5 (0.2)	0.6 (0.5)

### 3.3 Evidence of exposure

Plasma concentrations of acetamide were higher in all treatment groups and time-points compared to the negative controls for both mice and rats (Table 5). Based on the finding of Putcha et al. (1984), which showed that acetamide is rapidly and completely distributed in rat body tissues, these findings provide convincing evidence that bone marrow was exposed to acetamide since this tissue is highly perfused. Previous reports indicate little to no metabolism of acetamide (Dybing et al., 1987; Putcha et al., 1984); thus the primary exposure of the bone marrow (and the liver) is likely to the parent molecule.

**Table 5:** Evidence of tissue exposure to acetamide in both mice and rats.^a^.

	Dose (mg/kg/day)	Acetamide Concentration in Plasma in ppm (s.d)			
		Mice		Rats	
		Male	Female	Male	Female
Acute (2 days)	0	0.4 (0.1)	0.4 (0.1)	1.5 (0.3)	1.5 (0.1)
	250	35.7^2^ (14.8)	11.0^2^ (9.7)	187.0^2^ (56.5)	127.5^2^ (36.1)
	1000	171.3^2^ (106.0)	68.2^2^ (50.4)	405.5^2^ (71.2)	263.4^2^ (66.8)
	2000	183.5^2^ (81.7)	114.8^2^ (58.0)	827.6^2^ (261.0)	572.7^2^ (259.0)
Subchronic (28 days)	0	NA	NA	0.6 (0.1)	0.6 (0.1)
	1000	NA	NA	517.3^2^ (30.0)	348.3^2^ (95.2)
	1500	NA	NA	593.5^2^ (184.1)	456.0^2^ (197.1)

^2^Denotes significant difference (p ≤ 0.05) compared to species, sex, and time matched negative control.

a Data presented is mean of six animals per treatment group with standard deviation in parentheses.

### 3.4 Implications and conclusions

In its 2005 analysis (Abbott et al., 2006), JECFA referred to a positive *in vivo* mouse MN assay result by Chieli et al. (1987) and comet assay by Sasaki et al. (2000) on acetamide. Several groups attempted to reproduce the positive result of Chieli et al. (1987), investigating multiple strains of mice, animals of both sexes, higher doses of acetamide, different modes of administration, different sampling times post acetamide exposure, and use of both bone marrow and peripheral blood samples to quantify MN-PCE induction (De Boeck et al., 2005; Dybing et al., 1987; Miura et al., 1994; Morita et al., 1997). These studies showed that acetamide was inactive in inducing MN-PCE in mice under all conditions tested. The Sasaki et al. (2000) i.p. comet study was conducted at a single dose of 2.5X the currently recommended top dose and therefore, cannot be considered relevant.

The present studies were designed to provide a definitive conclusion regarding acetamide's clastogenicity/aneugenicity in an appropriately conducted two species rodent erythrocyte MN assay, that include confirmation of bone marrow exposure to acetamide. The doses that we used represent the limit doses recommended by the OECD test guideline and also represent the doses tested in the rodent cancer bioassay (Fleischman et al., 1980). In addition to demonstrating that acetamide does not induce MN-PCE in mice and rats, we demonstrate that acetamide is not a mutagen in the *Pig-a* gene mutation assay, consistent with the previous negative findings in the Ames test (Claxton et al., 1988; Dybing et al., 1987; ECHA, 2018; Emmert et al., 2006; Miura et al., 1994). Further investigations concerning the potential toxicity of acetamide were recently completed and there was no evidence for genotoxicity in the liver of rats in a 28-day transcriptomics study (Nault et al., 2019). Based on the results of our studies, we believe that acetamide should be reconsidered for regulatory evaluation.

## Funding

The work described in this paper was supported by a grant from the Bill and Melinda Gates Foundation [OPP1142801] to Michigan State University and its technology development affiliate, the Michigan Biotechnology Institute (MBI).

## Declaration of interests

The authors declare that they have no known competing financial interests or personal relationships that could have appeared to influence the work reported in this paper.

## Author contributions

The study was designed by MMM, BG, RN, FT and VB. The experiments were performed by scientists at Jai Research Foundation (RN, NK, MP, TK, AR) and Eurofins Advinus (ER). The data were evaluated by all of the authors. The manuscript was prepared by MMM, BG, RN, BB, and VB with input from all of the authors. All of the authors read and approved the final version of the manuscript prior to submission.

## Appendix A Supplementary data

Multimedia component 3 Multimedia component 3

The final, complete reports from JRF, Eurofins Advinus Ltd., and Litron Laboratories have been deposited into the Harvard Dataverse (http://dataverse.harvard.edu/dataverse/acetamide).
