# Supplementary material for: The food contaminant acetamide is not an in vivo clastogen, aneugen, or mutagen in rodent hematopoietic tissue
Source: Regul Toxicol Pharmacol. 2019 Nov;108:104451. doi: 10.1016/j.yrtph.2019.104451 (PMC6876283; doi:10.1016/j.yrtph.2019.104451)
Supplement: Multimedia component 2 [file mmc2.pdf]

JAI RESEARCH FOUNDATION

Original Final Report 2 of 2

STUDY TITLE

MICRONUCLEUS TEST OF ACETAMIDE IN RAT

DATA REQUIREMENT
GUIDELINES: OECD 474

STUDY DIRECTOR/REPORT AUTHOR: AVANI K. SOLANKI
avani.solanki@jrfonline.com

STUDY COMPLETION: NOVEMBER 15, 2017

SPONSOR

MICHIGAN STATE UNIVERSITY
220 TROWBRIGE RD, EAST LANSING MI
48824, UNITED STATES

TEST FACILITY

JAI RESEARCH FOUNDATION
DEPARTMENT OF TOXICOLOGY
VALVADA - 396 105
DIST. VALSAD
GUJARAT
INDIA

STATEMENT OF GOOD LABORATORY PRACTICE COMPLIANCE

Test item : Acetamide

Study Title : Micronucleus Test of Acetamide in Rat

Except as noted below, the study described in this report was conducted in compliance with the following Good Laboratory Practice Standard:

Organisation for Economic Co-operation and Development (OECD)
ENV/MC/CHEM (98)17 and all subsequent OECD consensus documents

Exception: Test item characterisation (composition), stability, and method of synthesis and location of documents for the synthesis is the responsibility of the Sponsor.

There were two amendments to the study plan generated (APPENDIX 6). There was no deviation from the study plan.

November 15, 2017
Avani K. Solanki, M.Sc. Date
Study Director

November 16, 2017
Manish V. Patel, Ph.D. Date
Test Facility Management

Sponsored and Submitted By:

JAI RESEARCH FOUNDATION

STATEMENT OF QUALITY ASSURANCE

Test item : Acetamide

Study Title : Micronucleus Test of Acetamide in Rat

This study was audited and the final report examined with respect to the protocol, standard operating procedures and raw data for conformance with the OECD Principles of Good Laboratory Practice. The report was determined to be a full and accurate reflection of the procedures adopted and the raw data generated during the study.

The audits were carried out according to the standard operating procedures of the Quality Assurance Unit of Jai Research Foundation (JRF) and in compliance with the OECD monograph N° 4, ENV/JM/MONO(99)20 (1999).

Findings resulting from the audits were reported to the Study Director and the Management on the dates specified below. These reports are kept in the GLP Archives at JRF.

Inspection/Audit			Reporting Dates to	
**N°**	**Details**	**Date**	**Study Director**	**Facility Management**
94463	Study plan	August 12, 2017	August 12, 2017	August 12, 2017
95834	Body weight, dosing (day 1) and dose formulation analysis	September 23, 2017	September 23, 2017	September 23, 2017
96042	Plasma sample analysis	September 29, 2017	September 29, 2017	September 29, 2017
96427	Slide scoring	October 12, 2017	October 12, 2017	October 12, 2017
96786	Raw data and report	October 27, 2017	October 27, 2017	October 27, 2017
97331	Final report	November 15, 2017	November 15, 2017	November 15, 2017

Number of study plan amendment(s) reviewed: 02

In addition, other processes related to this type of study were inspected periodically by the Quality Assurance. The most recent process inspected is identified below:

JAI RESEARCH FOUNDATION

STATEMENT OF QUALITY ASSURANCE (Continued)

Inspection			Reporting Dates to	
N°	Date	Details	Study Director	Facility Management
94871	Processes of micronucleus test	August 01, 2017 to August 25, 2017	August 25, 2017	August 25, 2017

Associated laboratory and support functions are subject to regular facility inspections in accordance with the Quality Assurance procedures.

BHAVYA PATEL, B.Pharm.
Jr. QUALITY ASSURANCE OFFICER, JRF
DATE: November 15, 2017

JAI RESEARCH FOUNDATION

CONTENTS

PAGE N°

TITLE PAGE ... 1
STATEMENT OF GOOD LABORATORY PRACTICE COMPLIANCE ... 2
STATEMENT OF QUALITY ASSURANCE ... 3
CONTENTS ... 5
SUMMARY ... 7
1. INTRODUCTION ... 8
1.1 Study Objective ... 8
1.2 Study Guidelines ... 8
1.3 Justification for Selection of the Test System ... 8
1.4 Test Facility and Study Period ... 8
1.5 Personnel Involved in the Study ... 8
1.6 Archives ... 9
2. EXPERIMENTAL PROCEDURE ... 10
2.1 Test Item ... 10
2.2 Positive Control ... 10
2.3 Solvent and Chemicals ... 11
2.4 Instruments and Equipment ... 11
2.5 Principle ... 12
2.6 Animal Welfare ... 12
2.7 Test Animals ... 12
2.8 Acclimatisation ... 12
2.9 Identification ... 12
2.10 Environmental Conditions ... 13
2.11 Husbandry Practices ... 13
2.12 Feed and Water ... 13
2.13 Selection of Vehicle ... 13
2.14 Rationale for Selection of Route of Administration ... 13
2.15 Main Study ... 14
2.16 Dose Formulation Preparation, Sampling and Analysis ... 14
2.16.1 Analytical Acceptance Criteria ... 15
2.17 Evidence of Tissue Exposure ... 15
2.18 Slide Preparation ... 16
2.19 Scoring of Bone Marrow Micronucleus ... 16
2.20 Calculation ... 16
2.21 Statistical Evaluation of Results ... 17

JAI RESEARCH FOUNDATION

2.22 Historical Control Data 17
2.23 Assay Acceptance and Evaluation Criteria 17
2.23.1 Acceptance Criteria 17
2.23.2 Evaluation and Interpretation Criteria 18
3. RESULTS 19
3.1 Main Study 19
3.1.1 Clinical Observations, Body Temperature and Body Weight 19
3.1.2 Micronucleated Polychromatic Erythrocytes 19
3.1.3 Dose Formulation Analysis 20
3.1.4 Evidence of Exposure 20
4. CONCLUSION 21
5. REFERENCES 22

TABLE 1: Summary of Mean Body Temperature- Main Study 24
TABLE 2: Summary of Mean Body Weight 26
TABLE 3: Summary of Micronucleated Polychromatic Erythrocytes in Bone Marrow Cells 27

APPENDIX 1: Individual Clinical Observations – Main Study 28
APPENDIX 2: Individual Body Temperature - Main Study 31
APPENDIX 3: Individual Body Weight (g) - Main Study 33
APPENDIX 4: Total Erythrocytes and P/E Ratio 35
APPENDIX 5: Frequency of Micronucleated Polychromatic Erythrocytes 37
APPENDIX 6: Signed Study Plan and Study Plan Amendments 39
APPENDIX 7: Dose Formulation Analysis and Plasma Sample Analysis Report 60
APPENDIX 8: Historical Control Data 95
APPENDIX 9: Water Analysis Reports 101
APPENDIX 10: Feed Analysis Reports 105
APPENDIX 11: Bedding Material Analysis Reports 107
APPENDIX 12: Certificate of Analysis of Acetamide (Provided by Supplier) 109
APPENDIX 13: Certificate of Analysis of Acetamide (Generated at JRF) 110
APPENDIX 14: GLP Endorsement of Compliance 111

JAI RESEARCH FOUNDATION

SUMMARY

STUDY TYPE : Micronucleus Test - Rat (RccHan: WIST); OECD 474 (July 2016)

TEST ITEM : Acetamide [99.2% w/w – Provided by Supplier; 99.198% w/w – Generated at JRF]

CITATION : Avani K. Solanki. Micronucleus Test of Acetamide in Rat. Jai Research Foundation, India. Laboratory report number: 485-1-06-17728; November 15, 2017.

SPONSOR : Michigan State University, U.S.A.

EXECUTIVE SUMMARY: This study was performed to evaluate the micronucleus induction potential of acetamide in rat. Sixty RccHan: WIST rats were divided into 5 groups, each group comprising 6 animals/sex. The main study was conducted at the dose levels of 250, 1000 and 2000 mg acetamide/kg body weight (Groups II, III and IV, respectively). A concurrent vehicle (distilled water) control group (Group I) was maintained along with the acetamide treated animals. Acetamide was dissolved in distilled water and administered orally for two consecutive days. Animals were sacrificed approximately between 18-24 hours after the final treatment. Before sacrifice blood samples were collected from each treatment group and vehicle control group to demonstrate the target organ exposure. A concurrent positive control group (Group V) was treated with a single intraperitoneal injection of Mitomycin-C at the dose level of 1 mg/kg body weight.

No toxicity to bone marrow [decrease in polychromatic to total erythrocytes ratio (P/E)] was observed in all animals treated at the dose levels of 250, 1000 and 2000 mg/kg body weight, when compared with the concurrent vehicle control group. All animals exhibited normal behavior and there were no mortalities. The number and percentage of micronucleated polychromatic erythrocyte (MNPCE) were not increased in animals treated with acetamide up to the dose level of 2000 mg/kg body weight when compared with the vehicle control group. No statistically significant effects on body weight were observed in any of the animals from positive control or treatment groups, when compared with the concurrent vehicle control group. The positive control group yielded a statistically significant increase in the number of micronucleated polychromatic erythrocytes (MNPCE) in comparison to the vehicle control group.

The dose formulation analysis revealed that the doses complied for the presence of test item for its nominal concentration (± 10%) of active ingredient (% CV < 10%). Plasma concentration of acetamide in different groups revealed that the test item reached the target tissue, i.e. bone marrow. Negative and positive control data were consistent with historical control distributions.

From the results of the present study, it is concluded that acetamide does not have micronucleus induction potential.

COMPLIANCE: Signed and dated GLP and Quality Assurance statements are provided. There was no deviation from regulatory requirements.

JAI RESEARCH FOUNDATION

1. INTRODUCTION

1.1 Study Objective

This study was performed to evaluate the micronucleus induction potential of acetamide in rat. The study was conducted in compliance with Principles of GLP (OECD 1998).

1.2 Study Guidelines

The present study was conducted according to:

OECD, 2016: The Organisation for Economic Co-operation and Development (OECD), Guidelines for Testing of Chemicals, Volume II, OECD 474, Mammalian Erythrocyte Micronucleus Test, adopted by the Council on July 29, 2016.

1.3 Justification for Selection of the Test System

The rat was selected as the test system of choice because it is a readily available rodent species. It has been historically shown to be a suitable model for assessing the micronucleus induction potential and is recommended by the OECD and other regulatory authorities. The results of the study are believed to be of value in predicting the micronucleus induction potential of the test item in humans.

1.4 Test Facility and Study Period

This study was performed at the Department of Toxicology, Jai Research Foundation, Valvada - 396 105, Dist. Valsad, Gujarat, India.

Study Initiation	:	August 30, 2017
Experiment Start	:	September 04, 2017
Experiment Completion	:	October 14, 2017
Study Completion	:	November 15, 2017

1.5 Personnel Involved in the Study

Study Director	:	Avani K. Solanki, M.Sc.
Deputy Study Director	:	Dr. Rajendra M. Nagane, M.V.Sc.
Study Personnel	:	Pradeep D. Tekale, M.Sc.
		Dibya Ranjan Panda, M.Sc.
		Durga N. Chejara, M.Pharm.
		Jainisha D. Rathod, M.Sc.
		Bindi S. Patel, M.Sc.
		Deval S. Mehta, Ph.D.
		Indrajitsinh. M. Barad, M.Sc.
		Geeta J. Singh, M.Sc.
		Akash D. Dhangar, M.Sc.
Statistical Analyst	:	Ranjit Singh, M.Sc. (Statistics)

JAI RESEARCH FOUNDATION

1.6 Archives

All original raw data including any storage medium for electronically recorded data, documentation, the signed study plan, the study plan amendments, the draft report, one original final report, slides and the representative sample of the test item will be retained in the GLP Archives at Jai Research Foundation for a period of ten years. At the end of this period, the Sponsor's instructions will be sought to either extend the archiving period or return the archived material to the Sponsor or dispose of the material.

JAI RESEARCH FOUNDATION

2. EXPERIMENTAL PROCEDURE

2.1 Test Item

Details of the test item provided by the Supplier:

Test Item Name	Acetamide
IUPAC Name	Acetamide
CAS Number	60-35-5
Molecular Formula	C_2H_5NO
Molecular Weight	59.07 g/mol
Molecular Structure	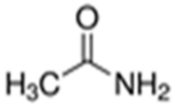
Batch/Lot Number	QYD4G
Purity<br>(Provided by Supplier)	99.2% w/w (Refer Certificate of Analysis in APPENDIX 12)
Purity (Generated at JRF)	99.198% w/w (Refer Certificate of Analysis in APPENDIX 13)
Manufactured by	Tokyo Chemical Industry Co. Ltd
Supplied to JRF by	Procured by JRF from Tokyo Chemical Industry Co. Ltd on behalf of sponsor
Date of Receipt	July 29, 2017
Retest Date	December 03, 2017
Appearance	White Solid
**Storage Condition (at JRF)**	As per the instruction received from the Sponsor on storage of the test item, the test item was stored :<br>Storage Temperature : Room temperature<br>Storage Container : In original container as supplied by the Sponsor<br>Storage Condition : Stored in its original container in isolated, dry, cool and well-ventilated area.<br>Storage Location : Test Item Control Office, JRF

Source of Molecular Weight, Molecular Formula and Molecular Structure: www.sigmaaldrich.com

2.2 Positive Control

Name	:	Mitomycin-C
Lot N°	:	SLBP4042V and SLBR5145V
CAS Number	:	50-07-7
Route of Administration	:	Intraperitoneal
Dose	:	1 mg/kg body weight (formulated at 0.1 mg/mL using distilled water as vehicle)
Appearance	:	Light grey powder and Blue-purple powder
Manufactured by	:	Sigma
Storage	:	2 - 8 °C (Amber vial)
Expiry date	:	April 2020 and February 2021

JAI RESEARCH FOUNDATION

2.3 Solvent and Chemicals

Methanol	:	Qualigens (Lot # 1655050117)
Foetal Bovine Serum	:	Himedia (Lot # 0000296898)
Giemsa Powder	:	Merck (Lot # DC6D660652)
Potassium Dihydrogen Orthophosphate	:	Qualigens (Lot #2301790714)
Sodium Hydroxide	:	Qualigens (Lot # 27287109-1)
Glycerol	:	Qualigens (Lot # 14687201-2)
NaH_2PO_4	:	Merck (Lot # QH3Q631840)
Na_2HPO_4	:	Sigma (Lot # BCBN1164V)
DPX Mountant	:	Qualigens (Lot # 1097020616)
Immersion Oil	:	Himedia (Lot # 0000248321)
Disinfectant	:	Dettol 2.5% v/v (Lot #D9354)
Heparin	:	Biological E. Ltd. (Lot #AK040)

2.4 Instruments and Equipment

Digital Balance	:	Adventurer/AR 2140, OHAUS<br>(Capable of measuring 10 mg to 210 g)
Electronic Balance	:	Electronic Weighing Scale - SMART<br>(Capable of measuring 5 g to 3 kg)
Metal Cannula	:	CW12 ILA, England, size: 16 G x 5 cm
Syringe	:	BD 1 mL and 3 mL disposable syringe
Needles	:	1. 26 G ½ (0.45 x 13 mm), BD Precision Glide<br>2. 24 G x 1" (0.6 x 25 mm), BD Precision Glide
Vacuum Desiccator	:	Tarsons (CO_2 Chamber)
Centrifuge	:	Thermoscientific
Binocular Microscopes	:	1. Eclipse E600, Nikon<br>2. Eclipse 80i, Nikon<br>3. Eclipse Ni-U (Fluorescence), Nikon<br>4. Eclipse Ci, Nikon
Multiportable Meter	:	Hach, USA
Tattoo Machine	:	AIMS^{TM} Tattoo Machine
Microprobe Thermometer	:	Physitemp Instruments Inc.
Refrigerator	:	LG Electronics Inc.
Bench Top Autoclave	:	Kumar, India
Deep-Freezer (-20 °C)	:	Vestfrost Solutions
Micropipettes	:	Eppendorf AG (100 – 1000 µL, 20-200 µL)
Millipore Water Purification System	:	Merck Millipore
Deep-Freezer (-80 °C)	:	SANYO
Ultrasonic Cleaning Bath	:	Cole Parmer, USA
Vortex Mixer	:	Remi Elecrotechnik
RO Systems	:	Hitech Water Technology Pvt. Ltd. and Kent RO System

2.5 Principle

The mammalian micronucleus test is used to detect cytogenetic damage (which results in a chromosomal break, fragment or lagging whole chromosome) caused by the test item. The damaged chromosomal fragments remain in the anucleated cytoplasm of the erythrocyte and are visible, when stained, as a small round or oblong structure called micronuclei. An increase in the frequency of micronucleated polychromatic erythrocytes in treated animals is an indication of induced chromosome damage.

2.6 Animal Welfare

The study was undertaken in compliance with the 'Guidelines for Laboratory Animals Facility' issued by the Committee for the Purpose of Control and Supervision of Experiments on Animals (CPCSEA), India. These guidelines promote the humane care of animals used in research by providing specifications that will enhance animal well-being and experimental quality for the advancement of biological knowledge that is relevant to humans and animals.

Project proposal for the experimentation was approved by Institutional Animal Ethics Committee (IAEC), Jai Research Foundation.

JRF is also accredited with Association for Assessment and Accreditation of Laboratory Animal Care (AAALAC) that promotes the humane treatment of animals in science.

2.7 Test Animals

For the main study, Rcc:Han:Wistar rats (*Rattus norvegicus*) were received from the Animal Breeding Facility, Jai Research Foundation. The animals were 9-10 weeks old on day 1 of dosing. The females used were nulliparous and non-pregnant. The male rats weighed between 239 and 282 g and the female rats weighed between 170 and 202 g on day 1 of the experiment (main study).

2.8 Acclimatisation

The animals were received into the experimental room and acclimatised for a period of seven days (maximum 3 animals/cage). The animals were randomised into 5 groups using validated in-house developed software. The method of randomisation used was censored randomisation method (Gad S.C. and Weil, C.S., 1994).

2.9 Identification

Before randomisation, animals were marked with nontoxic marker pen. After randomisation, each rat was assigned a number, which was tattooed on its tail using a tattoo machine and appropriate labels were attached to the cages indicating the study number, test item code, group number and sex, dose, type of study, cage number and animal number.

2.10 Environmental Conditions

Animal Room	:	BMR Facility Room No. 26, Department of Toxicology
Temperature Range	:	20- 24 °C
Relative Humidity Range	:	64 - 67%
Photoperiod	:	The photoperiod was 12 h artificial light and 12 h darkness, light hours being 06:00 h - 18:00 h.
Air Changes	:	Minimum 15 volumes/hour.

2.11 Husbandry Practices

Caging	:	Polypropylene rat cages (size: 41 x 28.2 x 15 cm) with stainless steel grid top. Autoclaved clean rice husk was used as the bedding material.
Water Bottle	:	Each cage was supplied with a polypropylene water bottle (capacity 300 mL) with a stainless steel nozzle.
Housing	:	3 animals per cage.
Room Sanitation	:	Each day the floor and all work tops were mopped with a disinfectant solution (Dettol 2.5% v/v).

2.12 Feed and Water

The quality of feed and water is regularly monitored at Jai Research Foundation. There were no known contaminants in the feed or water at levels that would have interfered with the experimental results obtained.

Feed : Rat pellet feed (Teklad, Certified Global 16% Protein Rodent Diet Sterilizable, USA) was provided *ad libitum* (except fasting for overnight before day 1 of dosing and 3 h after dosing) (APPENDIX 10).

Water : UV sterilized drinking water filtered through Hi-Tech reverse osmosis water filtration system was provided *ad libitum* (APPENDIX 9).

2.13 Selection of Vehicle

Acetamide was found soluble in distilled water (stock A, 200 mg/mL). Hence distilled water was selected as the vehicle for oral gavage for the animals in the main study.

2.14 Rationale for Selection of Route of Administration

A potential route of human exposure is via the oral route. Therefore, the oral route of administration was selected for this study.

JAI RESEARCH FOUNDATION

2.15 Main Study

Based on sponsor's suggestions and the published data from earlier studies (Michael R. *et al.,* 2014, Chieli *et al.,* 1987, Mirkova, 1996 and Dybing *et al*., 1987), the main study was conducted with dose levels of 250, 1000 and 2000 mg/kg body weight. Five groups (comprising 6 aniamls/sex) were used for this study. Group I was served as the vehicle (distilled water) control, Group II, III and IV were low, mid and high dose groups, respectively. Group V was the positive control group and received Mitomycin-C (1.0 mg/kg body weight on day 2 of treatment) in distilled water by the intraperitoneal route on a single occasion.

A quantity of 1250, 5000 and 10000 mg of acetamide were weighed and dissolved in distilled water on day 1 and day 2 of dosing (Gad and Cassidy, 2006). The volume was made up to 50 mL to attain a concentration of 25, 100 and 200 mg/mL for male and female animals for groups II, III, and IV, respectively. The dose volume was 10 mL/kg body weight for all the treatment groups including vehicle and positive control group. The acetamide was administered orally to rat using a metal cannula attached to a BD 1 mL disposable syringe. Rat from the vehicle control group (Group I) received only distilled water orally on both the days.

The rat from the positive control group (Group V) received a single injection of Mitomycin-C intraperitoneally at the dose level of 1.0 mg/kg body weight on day 2 of treatment. Each day the dose solutions were freshly prepared prior to dosing.

Body weight was recorded before dosing on day 1, day 2 and before sacrifice. The clinical signs of toxicity were recorded before dosing, post dosing (up to four hours) and before sacrifice. The body temperatures of all the animals were measured before dosing and then approximately 2 and 5 hours after each dosing and before sacrifice using microprobe thermometer (Asanami and Shimono, 1997; Asanami et al., 1998).

2.16 Dose Formulation Preparation, Sampling and Analysis

For active ingredient concentration analysis, samples were collected from each prepared dose formulations along with vehicle (distilled water) during the main study following the detailed procedures below.

Two sets of three replicates of 2 mL each concentration (25, 100 and 200 mg/mL for male and female animals) were taken from middle portion along with vehicle (distilled water). First set of replicates (three replicates of 2 mL each) were sent to Department of Chemistry (JRF) for analysis and second set of replicates were stored in deep freezer (-70 ± 10 ºC) as backup. The unused aliquots will be discarded after receiving approval for finalisation of the report from the sponsor.

Samples were analysed using following analytical parameters: (JRF Study N° 228-2-14-17729)

Instrumental Parameters

Instrument	:	GC-MS
Column	:	Agilent VF-5MS, 0.25 mm (i.d.), 30m length, 0.25 µm film thickness
Carrier Gas	:	Helium
Injection Volume	:	2.0 µL
Injection Temperature	:	250 °C
Flow Rate	:	1.2 mL/minute
Split Ratio	:	1:8
Oven Temperature	:	40 °C (Hold 2.0 minutes) to 20.0 °C to 300 °C, (Hold for 10 minutes) – Total of 25 minutes
Mass Spectrometry	:	Electron Ionization mode with 70 eV SIM Mode
Solvent Delay Time	:	4.0 minutes
Quadruple Temperature	:	150 °C
Data Acquisition	:	Selected Ion Monitoring (SIM) for masses 239 (Xanthyl-acetamide) and 253 (Xanthyl- Propionamide)

2.16.1 Analytical Acceptance Criteria

The following criteria for acceptable specification for the concentration of the test item in the vehicle were used to determine a valid assay:

90 to 110% of nominal concentration with < 10% coefficient of variance (% CV) of each concentration (Whitmire et al., 2010).

2.17 Evidence of Tissue Exposure

Blood samples were withdrawn from each animal in each treatment group and vehicle control group at the time of sacrifice before bone marrow collection. Blood samples were collected in heparinised (20 IU/mL) micro-centrifuge tubes. Blood samples were collected from orbital plexus under very light isoflurane anesthesia. To separate out the plasma, blood samples were centrifuged at 3000 rpm for 15 minutes at 4 °C. The plasma samples were stored at -70 ± 10 °C until analysis. The plasma samples were analysed for determination of test item concentration at Department of Chemistry, JRF.

Samples were analysed using following analytical parameters: (JRF Study N° 228-2-14-18476)

Instrumental Parameters

Instrument	:	GC-MS
Column	:	Agilent VF-5MS, 0.25 mm (i.d.), 30m length, 0.25 µm film thickness
Carrier Gas	:	Helium
Injection Volume	:	2.0 µL
Injection Temperature	:	250 °C
Flow Rate	:	1.2 mL/minute
Split Ratio	:	1:8

Oven Temperature	:	40 °C (Hold 2.0 minutes) to 20.0 °C to 300 °C, (hold for 10 minutes) – Total of 25 minutes
Mass Spectrometry	:	Electron Ionization mode with 70 eV
Data Acquisition	:	Selected Ion Monitoring (SIM) for masses 239 (Xanthyl-acetamide), 242 (Xanthyl-3d- acetamide) and 253 (Xanthyl- Propionamide)

2.18 Slide Preparation

Within 18 - 24 h following the last treatment, rat from the vehicle control and the treatment groups (group I - group IV) were sacrificed by CO_2 asphyxiation (MacGregor *et al.* 1987) and the positive control group (group V) was sacrificed 24 hour after the last treatment by CO_2 asphyxiation (Krishna and Hayashi, 2000). Femur bones from the sacrificed animals were excised and the epicondyle tips were removed. The bone marrow content was expelled by flushing and aspirating approximately 5 mL of foetal bovine serum using a 3 mL syringe and 24 gauge needle into centrifuge tubes. The aspirated bone marrow content was mixed using the syringe to dissociate the cells in order to avoid cell clump formation.

The tubes were centrifuged at around 1500 rpm for 10 minutes and the supernatant was discarded leaving about 0.2 - 0.3 mL of medium with the cell pellet. The cell pellet was dissociated thoroughly using a Pasteur pipette and a drop of suspension was placed on a clean slide. A smear was prepared and allowed to air dry.

The slides were marked with study number, animal number and slide number. Two slides were prepared per animal and the cells were fixed with methanol and allowed to air dry for 20 minutes. Slides were stained using 5% Giemsa in phosphate buffer for 25 minutes. Subsequently the slides were rinsed in distilled water, air-dried and mounted. In order to prevent bias in the scoring, the slide numbers were masked with code numbers provided by the Department of Bio-statistics and Systems Information, Jai Research Foundation.

2.19 Scoring of Bone Marrow Micronucleus

One out of two slides from each animal was used for screening of micronucleated erythrocytes whereas the other slide was kept as back up, to be used for scoring when required. The slides were examined for the presence of micronuclei in polychromatic and normochromatic erythrocytes under microscope [Nikon Eclipse E600, Nikon Eclipse 80i, Nikon Eclipse Ni-U (Fluorescence) and Nikon Eclipse Ci]. A minimum of 4000 polychromatic erythrocytes were screened per animal to evaluate the incidence of micronuclei. A minimum of 500 normochromatic erythrocytes to its corresponding polychromatic erythrocytes were recorded to determine the P/E ratio. The masked labels were removed and all the slides were decoded after scoring.

2.20 Calculation

The P/E ratios were calculated from polychromatic to total (polychromatic + normochromatic) erythrocytes. The percentage of micronucleated polychromatic erythrocytes was also calculated.

2.21 Statistical Evaluation of Results

The data of percent micronucleated polychromatic erythrocytes (% MNPCE), P/E ratio and body weight of both the sexes were statistically analysed for normality using Shapiro-Wilk's test. Where results of normality test were significant, non-parametric test (Kruskal-Walis test) was performed. Where results of normality test were non-significant then Bartlett test was performed to meet the homogeneity of variance before conducting ANOVA test followed by Dunnett's t-test. T-test was also performed to determine the level of significant difference between the vehicle control and the treated groups and positive control group.

2.22 Historical Control Data

Jai Research Foundation (JRF) has conducted GLP studies between 2012 and 2016 for regulatory submission as per OECD TG 474, and has established a historical control data base from 8 experiments for historical negative controls with male rats, 5 experiments for historical negative controls with female rats, and 4 experiments for historical positive controls with male rats. JRF uses quality control methods, such as control charts to identify data variability and to show that the methodology is 'under control'. Quality control charts (QC charts) have been added in the APPENDIX 8 demonstrating the JRF's established historical positive control ranges and distribution, and a historical negative control ranges and distribution.

Since TG 474 studies in rats are much less frequent than those using mice, the JRF historical data base, which spans the period from 2012 to 2016, contains less than the recommended number of historical experimental controls. The QC charts in APPENDIX 8 indicate that all historical control data lie within the 95% confidence intervals, illustrating the consistency and robustness of the JRF experimental procedures.

2.23 Assay Acceptance and Evaluation Criteria

Before assay data were evaluated, criteria for a valid assay had to be met. The following criteria were used to determine a valid assay:

2.23.1 Acceptance Criteria

i. The vehicle (or negative) controls values were in the range of historical control data.

ii. The positive controls has produced responses that were compatible with that of the historical data and has produce statistically significant responses compared with the concurrent negative control.

iii. Mortality was not observed in control or treatment group and six animals per sex per groups (group I to V) and six animals/sex were evaluated for micronucleus induction potential of the test item in all the groups.

iv. The highest dose was a limit dose, maximum tolerable dose (MTD) which did not cause distress or death to the animal or produce toxicity to bone marrow.

v. PCE to erythrocyte ratio was more than the 20% of the vehicle control.

2.23.2 Evaluation and Interpretation Criteria

Once criteria for a valid assay had been met, responses observed in the assay were evaluated. The conditions necessary for determining a positive result were,

i. At least one of the treatment groups exhibits statistically significant increase in the frequency of micronucleated polychromatic erythrocytes compared to concurrent negative control
ii. A positive result was defined as a dose-dependent, significant increase in the incidence of micronuclei when evaluated with an appropriate trend test e.g. Chi-square trend analysis.
iii. Statistical and biological relevance was considered in data interpretation.
iv. Any of the results falling outside the distribution of the historical negative control data i.e. Poisson based 95% control limits.

The test item was considered clearly negative, if, in all experimental conditions examined:

i. None of the treatment groups exhibits a statistically significant increase in the frequency of micronucleated immature erythrocytes compared with the concurrent negative control.
ii. There was no dose-related increase at any sampling time when evaluated by an appropriate trend test.
iii. All results were inside the distribution of the historical negative control data (e.g. Poisson-based 95% control limits), and
iv. Bone marrow exposure to the test item(s) occurred
v. There is no requirement for verification of a clear positive or clear negative response.

JAI RESEARCH FOUNDATION

3. RESULTS

3.1 Main Study

3.1.1 Clinical Observations, Body Temperature and Body Weight

All animals were normal in the vehicle control group (Group I) and treatment groups II, III and IV (250, 1000, 2000 mg/kg body weight, respectively) and positive control group (Group V), both post-treatment and pre-sacrifice.

Significant decrease or increase in body temperature was not observed after day 1 and day 2 of dosing both in male animals and female animals from treatment groups, when compared with the concurrent vehicle control group.

No statistically significant effect on mean body weight were observed in any of the animals from positive control or treatment groups, when compared with the concurrent vehicle control group. No mortalities were observed.

Individual clinical observations are provided in APPENDIX 1. The summary of mean body temperature and individual body temperature are provided in TABLE 1 and APPENDIX 2, respectively. The summary of mean body weight and individual body weight are provided in TABLE 2 and APPENDIX 3, respectively.

3.1.2 Micronucleated Polychromatic Erythrocytes

Value of P/E ratio and % MNPCE for vehicle and positive controls were within the 95% confidence-interval range of historical control data limits (APPENDIX 8).

No toxicity to bone marrow [decrease in polychromatic to total erythrocytes ratio (P/E)] was observed in both male and female animals treated at the dose levels of 250, 1000 and 2000 mg/kg body weight, when compared with the concurrent vehicle control group. Percent reduction in P/E ratio observed was 3.44, -0.81 and -0.61 in male animals treated at dose levels of 250, 1000, 2000 mg/kg bodyweight, respectively. Percent reduction in P/E ratio observed was -0.40, -0.81 and -2.63 in female animals treated at dose levels of 250, 1000, 2000 mg/kg bodyweight, respectively.

The ratio of polychromatic erythrocytes (PCE) to total erythrocytes (P/E ratio) in acetamide treated groups of male and female animals, treated at the dose levels of 250, 1000 and 2000 mg/kg body weight was comparable to the vehicle control group.

The mean P/E ratios observed in the male animals were 0.494, 0.477, 0.498 and 0.497 at the dose levels of 0.0 (vehicle control group), 250, 1000 and 2000 mg of acetamide/kg body weight, respectively. The mean P/E ratios observed in the female animals were 0.495, 0.497, 0.491 and 0.508 at the dose levels of 0.0 (vehicle control group), 250, 1000 and 2000 mg of acetamide/kg body weight, respectively. The mean polychromatic to total erythrocytes ratios (P/E) observed in the male and female animals treated with Mitomycin-C (1.0 mg/kg body weight) were 0.514 and 0.502, respectively.

The mean percent micronucleated polychromatic erythrocytes (% MNPCE) observed in male animals was 0.013, 0.013, 0.020 and 0.017 at the dose levels of 0.0 (vehicle control group), 250, 1000 and 2000 mg of acetamide/kg body weight, respectively. The mean percent micronucleated polychromatic erythrocytes (% MNPCE) observed in female animals was 0.017, 0.020, 0.020 and 0.013 at the dose levels of 0.0 (vehicle control group), 250, 1000 and 2000 mg of acetamide/kg body weight, respectively. The mean percent micronucleated polychromatic erythrocytes (% MNPCE) observed in male and female animals treated with Mitomycin-C (1.0 mg/kg body weight) were 0.927 and 0.957, respectively.

Statistical analysis of the results did not reveal any significant difference in percent micronucleated polychromatic erythrocytes (% MNPCE) in animals belonging to any treatment groups, when compared with the vehicle control group.

A statistically significant increase in mean % MNPCE observed in the male and female animals treated with Mitomycin-C (1.0 mg/kg body weight) demonstrated the sensitivity of the test system, suitability of the procedures and efficiency of the test conditions employed in the test (TABLE 3, APPENDIX 4 and APPENDIX 5).

Group-wise total polychromatic erythrocytes (PCE), micronucleated polychromatic erythrocytes (MNPCE), percent MNPCE and mean P/E ratio in bone marrow cells are given in TABLE 3 with individual data presented in APPENDIX 4 and APPENDIX 5.

3.1.3 Dose Formulation Analysis

The dose formulations complied with the presence of test item for its nominal concentration of (±10) active ingredient (% CV < 10%). Mean recoveries were 107.33, 106.28 and 106.03 at the prepared concentrations of 25, 100 and 200 mg/mL, respectively for both male and female animals (APPENDIX 7).

3.1.4 Evidence of Exposure

The plasma samples were analysed to demonstrate the target organ exposure, i.e., for test item concentration in blood. Dose dependent increase was observed in test item concentration in plasma samples (APPENDIX 7). Mean concentration observed at 250, 1000 and 2000 mg/kg body weight has been presented in below table:

Sex	Group and Dose (mg/kg body weight)	Mean Concentration in Plasma Samples (ppm)	Sex	Group and Dose (mg/kg body weight)	Mean Concentration in Plasma Samples (ppm)
Male	GI and 0.0	1.452	Female	GI and 0.0	1.527
	GII and 250	187.033		GII and 250	127.516
	GIII and 1000	405.517		GIII and 1000	263.448
	GIV and 2000	827.556		GIV and 2000	572.701

4. CONCLUSION

From the results of the present study, it is concluded that acetamide does not have micronucleus induction potential in male and female rats up to the dose level of 2000 mg/kg body weight, following oral administration for two consecutive days.

JAI RESEARCH FOUNDATION

5. REFERENCES

Asanami, S., and Shimono, K., 1997a: Hypothermia induces micronuclei in mouse bone marrow cells. Mutation Research. 393, pp. 91–98.

Asanami, S., and Shimono, K., 1997b: High body temperature induces micronuclei in mouse bone marrow. Mutation Research. 390, pp. 79–83.

Asanami, S., Shimono, K., and Kaneda, S., 1998: Transient hypothermia induces micronuclei in mice. Mutation Research. 413, 7–14.

Chieli, E., F. Aliboni, M. Saviozzi and G. Malvaldi 1987: Induction of micronucleated erythrocytes by primary thioamides and their metabolites in the mouse, Mutation Research 192, pp. 141-143.

Dybing et al., 1987: Studies on the Mechanism of Acetamide Hepatocarcinogenicity, Pharmacology & Toxicology, 60, pp. 9-16.

G. Krishna and M. Hayashi, 2000: In vivo Rodent Micronucleus Assay: Study plan Conduct and Data Interpretation, Mut.res. 455, pp. 155-166.

Gad, S.C. and Cassidy, C.D., 2006: Non-clinical Vehicles Use in Studies by Multiple Routes in Multiple Species, International Journal of Toxicology, 25, pp. 499 – 521

Gad, S.C. and Weil, C.S., 1994: "Statistics for Toxicologists". In: Principles and Methods of Toxicology, 3rd edition, Hayes A.W. (Ed), Raven press Ltd., New York, pp. 221-274.

Heddle, J.A., Stuart, E. and Salamone, M.F., 1984: The Bone Marrow Micronucleus Test, In: Hand Book of Mutagenicity Test Procedures. Second Edn., Kilbey B.J., M. Legator, W. Nichols, C. Ramel (Eds.) Elsevier, New York, pp. 441-457.

Khanvilkar T., 2017: "Validation of Analytical Method for Determination of Acetamide Concentration, Homogeneity and Stability in Vehicle", JRF Study Number: 228-2-14-17729, November 08, 2017, Study Report of Michigan State University, USA.

Khanvilkar T., 2017: "Validation of Analytical Method for Determination of Acetamide Concentration, Homogeneity and Stability in Plasma Samples", JRF Study Number: 228-2-14-18476, November 13, 2017, Study Report of Michigan State University, USA.

MacGregor, J.T., Heddle, J.A., Margolin, B.H., Ramel, C., Salamone, M.F., Tice, R.R. and Wild D., 1987: Guidelines for the Conduct of Micronucleus Assays in Mammalian Bone Marrow Erythrocytes, Mut. Res., 189, pp. 103-112.

Michael, R., Johannes, E., Ute, M., Markus, F. T., Simon, P., Heidrun Ellinger-Ziegelbauer, Andreas Z., 2014: Cross-Platform Toxicogenomics for the Prediction of Non-Genotoxic Hepatocarcinogenesis in Rat, Plos One, volume 9, Issue 5, May 2014.

JAI RESEARCH FOUNDATION

Mirkova, E.T., 1996: Activities of the rodent carcinogens thioacetamide and acetamide in the mouse bone marrow micronucleus assay, Mutation Research 352, pp. 23-30.

OECD, 1998 : OECD Series on Principles of Good Laboratory Practice and Compliance Monitoring, Number 1, "OECD Principles on Good Laboratory Practice", ENV/MC/CHEM(98)17 (as revised in 1997).

OECD, 2016: The Organisation for Economic Co-operation and Development (OECD) Guidelines for the Testing of Chemicals, Volume II, OECD 474, Mammalian Erythrocyte Micronucleus Test, adopted by the Council on July 29, 2016.

Putcha, L et al., 1984: Disposition of 14C-Acetohydroxamic acid and 14C- Acetamide in the Rat, Drug Metabolism and Disposition, The American Society for Pharmacology and Experimental Therapeutics, Volume 12, No. 4, pp. 438-443.

Whitmire, M.L., Bryan, P., Henry, T.R., Holbrook, J., Lehman, P., Mollitor, T., Ohorodnik, S., Reed, D., and Wietgrefe, H.D., 2010: "Nonclinical dose formulation analysis method validation and sample analysis". The AAPS Journal, 12(4), pp. 628-34.

Zhao, D et al., 2007: Comparative transport efficiencies of urea analogues through urea transporter UT-B, Biochimica et Biophysica Acta 1768 (2007), ScienceDirect, pp. 1815-1821.

JAI RESEARCH FOUNDATION

Micronucleus Test of Acetamide in Rat

TABLE 1: Summary of Mean Body Temperature- Main Study

Number of Animals = 6 Animals/Sex/Group **Refer: APPENDIX 2**

Group and Dose of Acetamide		Body Temperature (°C) After Dosing – Male						
		Before Dosing (Day 1)	After Dosing - Day 1 (hours)		Before Dosing (Day 2) (24 h after initial dose)	After Dosing - Day 2 (hours)		Before Sacrifice
			2 h	5 h		2 h	5 h	
G I Vehicle control (Distilled water)	**Mean**	37.0	36.9	37.1	36.9	36.9	37.0	37.0
	**SD**	0.3	0.3	0.3	0.2	0.2	0.3	0.2
G II (250 mg/kg body Weight)	**Mean**	37.0	37.0	37.1	37.0	37.0	37.0	37.2
	**SD**	0.3	0.3	0.2	0.4	0.1	0.2	0.2
G III (1000 mg/kg body weight)	**Mean**	37.1	36.9	37.0	36.9	36.9	37.1	37.2
	**SD**	0.3	0.3	0.2	0.3	0.1	0.3	0.2
G IV (2000 mg/kg body weight)	**Mean**	37.1	37.0	37.2	37.0	37.0	37.0	36.9
	**SD**	0.3	0.1	0.2	0.2	0.2	0.2	0.3
G V Positive control Mitomycin C (1.0 mg/kg body weight)	**Mean**	NA	NA	NA	36.9	36.9	37.0	37.1
	**SD**	NA	NA	NA	0.3	0.2	0.3	0.2

Keys : SD = Standard Deviation, °C = Degree centigrade, h = Hour, NA = Not applicable.

Note : Temperature of positive control animals was not recorded on day one since positive control animals were not treated on day one.

JAI RESEARCH FOUNDATION

TABLE 1 (Continued)

Group and Dose of Acetamide		Body Temperature (°C) After Dosing – Female						
		Before Dosing (Day 1)	After Dosing - Day 1 (hours)		Before Dosing (Day 2) (24 h after initial dose)	After Dosing - Day 2 (hours)		Before Sacrifice
			2 h	5 h		2 h	5 h	
G I Vehicle control (Distilled water)	**Mean**	36.4	36.5	36.4	36.5	36.6	36.4	37.0
	**SD**	0.1	0.1	0.2	0.2	0.3	0.2	0.2
G II (250 mg/kg body Weight)	**Mean**	37.1	37.1	37.0	37.0	37.1	37.0	37.1
	**SD**	0.3	0.2	0.2	0.3	0.2	0.3	0.2
G III (1000 mg/kg body weight)	**Mean**	37.0	37.0	37.1	36.8	36.9	36.9	37.0
	**SD**	0.2	0.2	0.2	0.1	0.3	0.3	0.2
G IV (2000 mg/kg body weight)	**Mean**	37.1	37.0	36.9	37.0	37.0	37.0	36.9
	**SD**	0.2	0.3	0.1	0.3	0.4	0.1	0.3
G V Positive control Mitomycin C (1.0 mg/kg body weight)	**Mean**	NA	NA	NA	36.9	36.9	36.9	37.1
	**SD**	NA	NA	NA	0.2	0.2	0.3	0.3

Keys : SD = Standard deviation, °C = Degree centigrade, h = Hour, NA = Not applicable.

Note : Temperature of positive control animals was not recorded on day one since positive control animals were not treated on day one.

JAI RESEARCH FOUNDATION

Micronucleus Test of Acetamide in Rat

TABLE 2: Summary of Mean Body Weight

Number of Animals = 6 Animals/Sex/Group **Refer: APPENDIX 3**

Group and Dose of Acetamide		Body Weight (g)					
		Male			Female		
		Day 1	Day 2	Before Sacrifice	Day 1	Day 2	Before Sacrifice
G I Vehicle control (Distilled water)	Mean	259.00	275.33	279.67	187.00	198.50	199.50
	SD	12.65	11.76	12.55	9.23	12.37	11.73
G II (250 mg/kg body Weight)	Mean	258.50	274.33	278.33	186.17	201.17	198.00
	SD	10.60	10.52	11.69	10.68	11.63	10.43
G III (1000 mg/kg body weight)	Mean	256.17	270.83	273.50	185.67	196.50	195.67
	SD	10.26	9.11	6.66	7.50	7.53	11.04
G IV (2000 mg/kg body weight)	Mean	255.83	274.00	271.50	186.17	199.33	196.17
	SD	10.72	11.54	11.69	10.50	10.95	8.33
G V (Mitomycin-C,1.0 mg/kg body weight)	Mean	NA	277.50	280.33	NA	202.00	202.33
	SD	NA	9.85	11.94	NA	10.64	9.00

Keys: SD = Standard deviation, NA = Not Applicable

Note: Body weight of positive control animals was not recorded on day one since positive control animals were not treated on day one.

JAI RESEARCH FOUNDATION

Micronucleus Test of Acetamide in Rat

TABLE 3: Summary of Micronucleated Polychromatic Erythrocytes in Bone Marrow Cells

Number of Animals = 6 Animals/Sex/Group **Refer: APPENDIX 4 and APPENDIX 5**

Group and Dose of Acetamide	Male					Female				
	Total PCE	MNPCE			P/E Ratio (Mean ± SD)	Total PCE	MNPCE			P/E Ratio (Mean ± SD)
		Total	Mean ± SD	%MNPCE (Mean ± SD)			Total	Mean ± SD	%MNPCE (Mean ± SD)	
G I Vehicle control (Distilled water)	27072	4	0.667 ± 0.816	0.013 ± 0.016	0.494 ± 0.031	27223	5	0.833 ± 0.753	0.017 ± 0.015	0.495 ± 0.009
G II (250 mg/kg body weight)	27060	4	0.667 ± 0.516	0.013 ± 0.010	0.477 ± 0.018	27079	6	1.000 ± 0.894	0.020 ± 0.018	0.497 ± 0.028
G III (1000 mg/kg body weight)	27100	6	1.000 ± 0.894	0.020 ± 0.018	0.498 ± 0.016	27055	6	1.000 ± 0.894	0.020 ± 0.018	0.491 ± 0.020
G IV (2000 mg/kg body weight)	27097	5	0.833 ± 0.753	0.017 ± 0.015	0.497 ± 0.027	27115	4	0.667 ± 0.516	0.013 ± 0.010	0.508 ± 0.025
G V (Mitomycin-C,1.0 mg/kg body weight)	27157	252	42.000↑↑ ± 5.865	0.927↑↑ ± 0.129	0.514 ± 0.026	27054	259	43.167↑↑ ± 12.703	0.957↑↑ ± 0.283	0.502 ± 0.028

Note:

$$\% \text{ MNPCE} = \frac{\text{MNPCE x 100}}{\text{Total PCE}}$$

Keys:
- PCE = Polychromatic Erythrocytes
- MNPCE = Micronucleated Polychromatic Erythrocytes
- P/E = Polychromatic Erythrocytes corresponding to Normochromatic Erythrocytes/Total Erythrocyte
- ↑↑ = Significantly higher than the control at 1% level ($p \leq 0.01$)

JAI RESEARCH FOUNDATION

Micronucleus Test of Acetamide in Rat

APPENDIX 1: Individual Clinical Observations – Main Study

Group and Dose of Acetamide	Sex	Animal N°	Individual Animal Observations on Experimental Days										Before Sacrifice
			Before Dosing (Day 1)	After Dosing Day 1 (hours)				Before Dosing (Day 2)	After Dosing Day 2 (hours)				
				1	2	3	4		1	2	3	4	
G I Vehicle control (Distilled water)	M	L1	1	1	1	1	1	1	1	1	1	1	1
		L2	1	1	1	1	1	1	1	1	1	1	1
		L3	1	1	1	1	1	1	1	1	1	1	1
		L4	1	1	1	1	1	1	1	1	1	1	1
		L5	1	1	1	1	1	1	1	1	1	1	1
		L6	1	1	1	1	1	1	1	1	1	1	1
	F	L7	1	1	1	1	1	1	1	1	1	1	1
		L8	1	1	1	1	1	1	1	1	1	1	1
		L9	1	1	1	1	1	1	1	1	1	1	1
		L10	1	1	1	1	1	1	1	1	1	1	1
		L11	1	1	1	1	1	1	1	1	1	1	1
		L12	1	1	1	1	1	1	1	1	1	1	1
G II (250 mg/kg body weight)	M	L13	1	1	1	1	1	1	1	1	1	1	1
		L14	1	1	1	1	1	1	1	1	1	1	1
		L15	1	1	1	1	1	1	1	1	1	1	1
		L16	1	1	1	1	1	1	1	1	1	1	1
		L17	1	1	1	1	1	1	1	1	1	1	1
		L18	1	1	1	1	1	1	1	1	1	1	1
	F	L19	1	1	1	1	1	1	1	1	1	1	1
		L20	1	1	1	1	1	1	1	1	1	1	1
		L21	1	1	1	1	1	1	1	1	1	1	1
		L22	1	1	1	1	1	1	1	1	1	1	1
		L23	1	1	1	1	1	1	1	1	1	1	1
		L24	1	1	1	1	1	1	1	1	1	1	1

Keys: M = Male, F = Female, 1 = Normal.

JAI RESEARCH FOUNDATION

APPENDIX 1 (Continued)

Group and Dose of Acetamide	Sex	Animal N°	Individual Animal Observations on Experimental Days										Before Sacrifice
			Before Dosing (Day 1)	After Dosing Day 1 (hours)				Before Dosing (Day 2)	After Dosing Day 2 (hours)				
				1	2	3	4		1	2	3	4	
G II (1000 mg/kg body weight)	M	L25	1	1	1	1	1	1	1	1	1	1	1
		L26	1	1	1	1	1	1	1	1	1	1	1
		L27	1	1	1	1	1	1	1	1	1	1	1
		L28	1	1	1	1	1	1	1	1	1	1	1
		L29	1	1	1	1	1	1	1	1	1	1	1
		L30	1	1	1	1	1	1	1	1	1	1	1
	F	L31	1	1	1	1	1	1	1	1	1	1	1
		L32	1	1	1	1	1	1	1	1	1	1	1
		L33	1	1	1	1	1	1	1	1	1	1	1
		L34	1	1	1	1	1	1	1	1	1	1	1
		L35	1	1	1	1	1	1	1	1	1	1	1
		L36	1	1	1	1	1	1	1	1	1	1	1
G III (2000 mg/kg body weight)	M	L37	1	1	1	1	1	1	1	1	1	1	1
		L38	1	1	1	1	1	1	1	1	1	1	1
		L39	1	1	1	1	1	1	1	1	1	1	1
		L40	1	1	1	1	1	1	1	1	1	1	1
		L41	1	1	1	1	1	1	1	1	1	1	1
		L42	1	1	1	1	1	1	1	1	1	1	1
	F	L43	1	1	1	1	1	1	1	1	1	1	1
		L44	1	1	1	1	1	1	1	1	1	1	1
		L45	1	1	1	1	1	1	1	1	1	1	1
		L46	1	1	1	1	1	1	1	1	1	1	1
		L47	1	1	1	1	1	1	1	1	1	1	1
		L48	1	1	1	1	1	1	1	1	1	1	1
G V Positive control Mitomycin-C (1.0 mg/kg body weight)	M	L49	1	-	-	-	-	1	1	1	1	1	1
		L50	1	-	-	-	-	1	1	1	1	1	1
		L51	1	-	-	-	-	1	1	1	1	1	1
		L52	1	-	-	-	-	1	1	1	1	1	1
		L53	1	-	-	-	-	1	1	1	1	1	1
		L54	1	-	-	-	-	1	1	1	1	1	1

Keys: M = Male, F = Female, 1 = Normal, - = Not applicable (Animals of positive control group were not treated on day one).

SAI RESEARCH FOUNDATION

APPENDIX 1 (Continued)

Group and Dose of Acetamide	Sex	Animal N°	Clinical Signs Observed after Dosing on										Before Sacrifice
			Before Dosing (Day 1)	After Dosing Day 1 (hours)				Before Dosing (Day 2)	After Dosing Day 2 (hours)				
				1	2	3	4		1	2	3	4	
G V (Mitomycin-C, 1.0 mg/kg body weight)	F	L55	1	-	-	-	-	1	1	1	1	1	1
		L56	1	-	-	-	-	1	1	1	1	1	1
		L57	1	-	-	-	-	1	1	1	1	1	1
		L58	1	-	-	-	-	1	1	1	1	1	1
		L59	1	-	-	-	-	1	1	1	1	1	1
		L60	1	-	-	-	-	1	1	1	1	1	1

Keys: F = Female, 1 = Normal, - = Not applicable (Animals of positive control group were not treated on day one)

JAI RESEARCH FOUNDATION

Micronucleus Test of Acetamide in Rat

APPENDIX 2: Individual Body Temperature - Main Study

Temperature Data – Male								
Group and Dose of Acetamide	Animal N°	Day of Dosing						
		Before Dosing (Day 1)	After Dosing Day - 1 (hours)		Before Dosing (Day 2)	After Dosing Day - 2 (hours)		Before Sacrifice
			2 h	5 h		2 h	5 h	
		°C	°C	°C	°C	°C	°C	°C
G I Vehicle control (Distilled water)	L1	36.7	36.7	37.0	36.9	36.7	37.1	36.9
	L2	37.1	37.0	37.3	37.0	36.9	37.2	37.1
	L3	36.9	36.5	36.8	37.0	36.6	36.7	37.0
	L4	37.3	37.2	37.0	36.9	36.8	37.4	36.8
	L5	37.2	37.0	37.5	36.9	37.2	37.1	37.2
	L6	36.7	36.8	36.7	36.6	37.0	36.6	37.1
G II (250 mg/kg body weight)	L13	36.6	36.7	36.9	36.8	37.0	36.8	37.1
	L14	37.1	37.0	37.3	37.2	37.1	36.9	36.9
	L15	37.4	37.5	37.0	37.6	36.9	37.2	37.4
	L16	37.2	37.0	37.1	37.2	36.8	36.9	37.5
	L17	36.9	36.7	37.0	36.6	37.1	37.2	37.0
	L18	37.0	37.3	37.2	36.7	36.9	37.1	37.3
G III (1000 mg/kg body weight)	L25	37.0	36.4	37.1	37.2	36.9	37.0	37.1
	L26	36.9	36.5	36.8	36.6	37.0	36.7	36.9
	L27	36.6	36.9	36.9	36.7	37.1	36.8	37.2
	L28	37.2	37.0	37.3	36.9	36.8	37.1	37.3
	L29	37.3	37.1	37.0	37.2	36.9	37.4	37.1
	L30	37.4	37.2	36.9	37.0	36.8	37.3	37.4
G IV (2000 mg/kg body weight)	L37	37.2	36.9	37.0	36.7	37.1	36.8	37.2
	L38	37.3	36.8	37.2	36.9	36.8	37.1	36.8
	L39	37.4	37.0	37.3	37.0	37.2	36.9	36.7
	L40	36.7	36.9	36.9	37.0	37.1	37.2	37.4
	L41	36.9	36.9	37.2	37.2	37.0	36.8	36.8
	L42	37.0	37.2	37.4	36.9	36.8	37.1	36.7
G V Positive control Mitomycin-C (1.0 mg/kg body weight)	L49	-	-	-	36.5	36.7	37.1	36.9
	L50	-	-	-	37.0	37.3	36.9	37.3
	L51	-	-	-	36.9	37.0	37.2	37.4
	L52	-	-	-	37.2	36.8	37.3	36.8
	L53	-	-	-	37.3	37.0	37.1	37.0
	L54	-	-	-	36.6	36.7	36.5	36.9

Note: Range of microprobe thermometer is -100 °C to +200 °C.

Keys: °C = Degree Centigrade, h = Hour, - = Not applicable (Positive control animals were not treated on day one).

JAI RESEARCH FOUNDATION

APPENDIX 2 (Continued)

Temperature Data –Female								
Group and Dose of Acetamide	Animal N°	Day of Dosing						
		Before Dosing (Day 1)	After Dosing Day – 1 (hours)		Before Dosing (Day 2)	After Dosing Day – 2 (hours)		Before Sacrifice
			2 h	5 h		2 h	5 h	
		°C	°C	°C	°C	°C	°C	°C
G I Vehicle control (Distilled water)	L7	36.3	36.4	36.3	36.2	36.6	36.4	37.1
	L8	36.5	36.6	36.4	36.6	37.1	36.5	36.8
	L9	36.4	36.4	36.6	36.3	36.2	36.5	37.2
	L10	36.4	36.5	36.3	36.8	36.6	36.7	37.1
	L11	36.2	36.4	36.7	36.5	36.3	36.3	36.8
	L12	36.5	36.7	36.3	36.4	36.6	36.2	37.0
G II (250 mg/kg body weight)	L19	37.2	37.1	37.3	37.0	37.4	37.1	37.4
	L20	36.7	36.9	36.8	37.1	36.9	37.0	36.8
	L21	36.6	37.0	36.9	36.5	36.7	36.4	36.9
	L22	37.2	37.1	36.9	36.7	37.2	37.3	37.1
	L23	37.4	37.2	37.0	37.5	37.1	37.3	37.2
	L24	37.2	37.4	36.9	36.9	37.1	37.0	36.9
G III (1000 mg/kg body weight)	L31	37.2	37.0	37.3	36.9	37.2	36.8	36.9
	L32	36.8	37.1	37.2	37.0	36.4	36.7	37.0
	L33	36.8	37.0	36.9	36.7	36.8	36.6	36.9
	L34	37.0	36.9	37.2	36.8	36.8	37.4	37.2
	L35	37.3	37.4	37.0	36.9	37.2	37.1	37.0
	L36	37.1	36.8	37.1	36.7	36.9	36.9	36.7
G IV (2000 mg/kg body weight)	L43	37.2	37.0	36.9	37.1	37.3	37.2	37.1
	L44	37.2	37.3	36.8	36.9	36.6	37.1	36.8
	L45	36.8	37.0	36.7	36.8	36.5	37.1	37.2
	L46	37.1	36.6	36.8	37.3	37.2	37.0	36.5
	L47	37.2	36.7	36.9	37.4	37.3	37.0	36.9
	L48	36.9	37.4	37.1	36.6	37.3	36.8	36.8
G V Positive control Mitomycin-C (1.0 mg/kg body weight)	L55	-	-	-	36.7	37.1	36.9	37.2
	L56	-	-	-	36.7	36.5	36.4	36.8
	L57	-	-	-	37.2	36.9	37.1	37.0
	L58	-	-	-	37.0	36.8	37.2	37.5
	L59	-	-	-	36.7	36.9	37.0	37.4
	L60	-	-	-	36.9	37.2	36.8	36.9

Note: Range of microprobe thermometer is -100 °C to +200 °C.

Keys: °C = Degree Centigrade, h = Hour

JAI RESEARCH FOUNDATION

Micronucleus Test of Acetamide in Rat

APPENDIX 3: Individual Body Weight (g) - Main Study

Group and Dose of Acetamide	Sex	Animal N°	Body Weight (g)		
			Day 1	Day 2	Before Sacrifice
G I Vehicle control (Distilled water)	Male	L1	282	296	302
		L2	262	274	279
		L3	260	280	278
		L4	253	265	277
		L5	250	273	279
		L6	247	264	263
	Female	L7	202	220	222
		L8	189	199	202
		L9	189	198	193
		L10	187	199	196
		L11	180	193	194
		L12	175	182	190
G II (250 mg/kg body weight)	Male	L13	277	293	301
		L14	264	280	279
		L15	254	270	275
		L16	257	270	275
		L17	251	269	272
		L18	248	264	268
	Female	L19	197	214	204
		L20	196	212	213
		L21	188	204	203
		L22	189	202	193
		L23	177	190	190
		L24	170	185	185
G III (1000 mg/kg body Weight)	Male	L25	267	281	280
		L26	264	275	279
		L27	264	280	278
		L28	253	267	271
		L29	246	262	270
		L30	243	260	263

JAI RESEARCH FOUNDATION

APPENDIX 3 (Continued)

Group and Dose of Acetamide	Sex	Animal N°	Body Weight (g)		
			Day 1	Day 2	Before Sacrifice
G III (1000 mg/kg body Weight)	Female	L31	193	204	205
		L32	190	204	206
		L33	192	198	201
		L34	185	196	198
		L35	180	193	184
		L36	174	184	180
G IV (2000 mg/kg body Weight)	Male	L37	270	288	280
		L38	261	280	279
		L39	260	280	281
		L40	256	273	273
		L41	249	268	265
		L42	239	255	251
	Female	L43	197	211	201
		L44	194	208	204
		L45	191	204	197
		L46	188	201	203
		L47	177	188	188
		L48	170	184	184
G V (Mitomycin-C,1.0 mg/kg body weight)	Male	L49	-	287	290
		L50	-	285	292
		L51	-	285	288
		L52	-	272	274
		L53	-	274	277
		L54	-	262	261
	Female	L55	-	213	209
		L56	-	207	206
		L57	-	209	211
		L58	-	201	202
		L59	-	199	200
		L60	-	183	186

Note: Body weight of positive control animals was not recorded on day one since positive control animals were not treated on day one.

JAI RESEARCH FOUNDATION

Micronucleus Test of Acetamide in Rat

APPENDIX 4: Total Erythrocytes and P/E Ratio

Group and Dose of Acetamide	Sex	Animal N°	Total PCE Scored	PCE Corr. to NCE	NCE Scored	Total Erythrocytes	P/E Ratio
G I Vehicle control (Distilled water)	Male	L1	4508	256	265	521	0.491
		L2	4510	284	249	533	0.533
		L3	4514	244	312	556	0.439
		L4	4520	268	262	530	0.506
		L5	4510	254	265	519	0.489
		L6	4510	257	253	510	0.504
	Female	L7	4503	259	271	530	0.489
		L8	4513	266	263	529	0.503
		L9	4516	251	257	508	0.494
		L10	4507	249	262	511	0.487
		L11	4678	255	266	521	0.489
		L12	4506	263	253	516	0.510
G II (250 mg/kg body weight)	Male	L13	4504	252	297	549	0.459
		L14	4513	251	263	514	0.488
		L15	4511	255	277	532	0.479
		L16	4501	259	266	525	0.493
		L17	4515	254	263	517	0.491
		L18	4516	250	304	554	0.451
	Female	L19	4509	267	244	511	0.523
		L20	4506	257	269	526	0.489
		L21	4529	248	304	552	0.449
		L22	4511	259	256	515	0.503
		L23	4513	254	263	517	0.491
		L24	4511	282	253	535	0.527
G III (1000 mg/kg body weight)	Male	L25	4514	247	270	517	0.478
		L26	4532	291	272	563	0.517
		L27	4509	260	251	511	0.509
		L28	4512	260	258	518	0.502
		L29	4517	259	259	518	0.500
		L30	4516	246	266	512	0.480

Note: Polychromatic erythrocytes corresponding to normochromatic erythrocytes were recorded (minimum 500 erythrocytes) for calculating the (P/E) ratio.

Keys: PCE = Polychromatic Erythrocytes, NCE = Normochromatic Erythrocytes.

P/E = Polychromatic Erythrocytes corresponding to Normochromatic Erythrocytes/Total Erythrocytes.

JAI RESEARCH FOUNDATION

APPENDIX 4 (Continued)

Group and Dose of Acetamide	Sex	Animal N°	Total PCE Scored	PCE Corr. to NCE	NCE Scored	Total Erythrocytes	P/E Ratio
G III (1000 mg/kg body weight)	Female	L31	4509	253	270	523	0.484
		L32	4505	243	262	505	0.481
		L33	4516	283	260	543	0.521
		L34	4504	257	301	558	0.461
		L35	4507	251	254	505	0.497
		L36	4514	257	256	513	0.501
G IV (2000 mg/kg body weight)	Male	L37	4514	260	264	524	0.496
		L38	4525	292	241	533	0.548
		L39	4514	262	263	525	0.499
		L40	4507	255	272	527	0.484
		L41	4513	257	277	534	0.481
		L42	4524	266	295	561	0.474
	Female	L43	4519	289	250	539	0.536
		L44	4509	297	254	551	0.539
		L45	4513	246	258	504	0.488
		L46	4559	253	264	517	0.489
		L47	4510	276	267	543	0.508
		L48	4505	261	277	538	0.485
G V (Mitomycin-C,1.0 mg/kg body weight)	Male	L49	4507	247	275	522	0.473
		L50	4544	275	255	530	0.519
		L51	4510	284	254	538	0.528
		L52	4526	272	254	526	0.517
		L53	4520	285	234	519	0.549
		L54	4550	252	253	505	0.499
	Female	L55	4508	254	258	512	0.496
		L56	4513	253	276	529	0.478
		L57	4507	253	288	541	0.468
		L58	4515	283	243	526	0.538
		L59	4505	253	253	506	0.500
		L60	4506	289	254	543	0.532

Note: Polychromatic erythrocytes corresponding to normochromatic erythrocytes were recorded (minimum 500 erythrocytes) for calculating the (P/E) ratio.

Keys: PCE = Polychromatic Erythrocytes, NCE = Normochromatic Erythrocytes.

P/E = Polychromatic Erythrocytes corresponding to Normochromatic Erythrocytes/Total Erythrocyte.

JAI RESEARCH FOUNDATION

Micronucleus Test of Acetamide in Rat

APPENDIX 5: Frequency of Micronucleated Polychromatic Erythrocytes

Group and Dose of Acetamide	Sex	Animal N°	Total Number of PCE Scored	Number of MNPCE	Percent MNPCE
G I Vehicle control (Distilled water)	Male	L1	4508	0	0.00
		L2	4510	0	0.00
		L3	4514	2	0.04
		L4	4520	0	0.00
		L5	4510	1	0.02
		L6	4510	1	0.02
	Female	L7	4503	1	0.02
		L8	4513	2	0.04
		L9	4516	0	0.00
		L10	4507	1	0.02
		L11	4678	1	0.02
		L12	4506	0	0.00
G II (250 mg/kg body weight)	Male	L13	4504	1	0.02
		L14	4513	1	0.02
		L15	4511	1	0.02
		L16	4501	1	0.02
		L17	4515	0	0.00
		L18	4516	0	0.00
	Female	L19	4509	1	0.02
		L20	4506	2	0.04
		L21	4529	1	0.02
		L22	4511	2	0.04
		L23	4513	0	0.00
		L24	4511	0	0.00
G III (1000 mg/kg body Weight)	Male	L25	4514	2	0.04
		L26	4532	1	0.02
		L27	4509	1	0.02
		L28	4512	2	0.04
		L29	4517	0	0.00
		L30	4516	0	0.00

Keys: PCE = Polychromatic Erythrocytes, MNPCE = Micronucleated Polychromatic Erythrocytes, Percent MNPCE = MNPCE x 100/Total PCE.

JAI RESEARCH FOUNDATION

APPENDIX 5 (Continued)

Group and Dose of Acetamide	Sex	Animal N°	Total Number of PCE Scored	Number of MNPCE	Percent MNPCE
G III (1000 mg/kg body weight)	Female	L31	4509	2	0.04
		L32	4505	0	0.00
		L33	4516	1	0.02
		L34	4504	2	0.04
		L35	4507	1	0.02
		L36	4514	0	0.00
G IV (2000 mg/kg body weight)	Male	L37	4514	1	0.02
		L38	4525	1	0.02
		L39	4514	0	0.00
		L40	4507	1	0.02
		L41	4513	2	0.04
		L42	4524	0	0.00
	Female	L43	4519	0	0.00
		L44	4509	1	0.02
		L45	4513	1	0.02
		L46	4559	1	0.02
		L47	4510	0	0.00
		L48	4505	1	0.02
G V (Mitomycin-C,1.0 mg/kg body weight)	Male	L49	4507	43	0.95
		L50	4544	51	1.12
		L51	4510	46	1.02
		L52	4526	39	0.86
		L53	4520	35	0.77
		L54	4550	38	0.84
	Female	L55	4508	37	0.82
		L56	4513	59	1.31
		L57	4507	60	1.33
		L58	4515	35	0.78
		L59	4505	34	0.75
		L60	4506	34	0.75

Keys: PCE = Polychromatic Erythrocytes, MNPCE = Micronucleated Polychromatic Erythrocytes and Percent MNPCE = MNPCE x 100/Total PCE

JAI RESEARCH FOUNDATION

APPENDIX 6: Signed Study Plan and Study Plan Amendments

STUDY PLAN

MICRONUCLEUS TEST OF ACETAMIDE IN RAT

GUIDELINES: OECD 474

SPONSOR

**MICHIGAN STATE UNIVERSITY,
220 TROWBRIGE RD, EAST LANSING MI,
48824, UNITED STATES**

STUDY DIRECTOR: AVANI K. SOLANKI

TEST FACILITY

**JAI RESEARCH FOUNDATION
DEPARTMENT OF TOXICOLOGY
VALVADA - 396 105
DIST. VALSAD
GUJARAT
INDIA**

AUGUST - 2017

JAI RESEARCH FOUNDATION

APPENDIX 6 (Continued)

CONTENTS

PAGE N°

TITLE PAGE..1
CONTENTS..2
1. GENERAL INFORMATION..4
1.1 Study Director..4
1.2 Test Facility Management..4
1.3 Study Schedule..4
1.4 Study Plan and Amendment (if any) Distribution..4
2. INTRODUCTION..4
2.1 Objective..4
2.2 Regulatory Guidelines..4
2.3 Principle of the Test Method..5
2.4 Test Item..5
3. GOOD LABORATORY PRACTICE (GLP)..6
3.1 GLP Compliance..6
3.2 Standard Operating Procedures (SOPs)..6
3.3 Amendment to Study Plan..6
3.4 Deviation(s)..6
3.5 Quality Assurance..7
4. ANIMAL WELFARE..7
4.1 Humane Endpoint..7
5. EXPERIMENTAL PROCEDURE..7
5.1 Initial Considerations..7
5.2 Reason for Selection of the Test System..8
5.3 Animals 8
5.4 Acclimatisation..8
5.5 Housing and Animal Identification..8
5.6 Animal Room Sanitation..8
5.7 Feed and Water..8
5.8 Environmental Conditions..9
5.9 Selection of Vehicle..9

JAI RESEARCH FOUNDATION

APPENDIX 6 (Continued)

5.10 Dose Formulation Preparation, Sampling and Analysis 9

5.10.1 Analytical Acceptance Criteria 10

5.11 Main Study 10

5.12 Evidence of Tissue Exposure 11

5.13 Study Performance 12

5.14 Historical control data 12

5.15 Microscopic Observation 12

5.16 Statistical Analysis 13

5.17 Assay Acceptance and Evaluation Criteria 13

5.17.1 Acceptance Criteria 13

5.17.2 Evaluation and Interpretation Criteria 13

6. REPORT 14

7. ARCHIVES 15

8. REFERENCES 16

9. STUDY PLAN APPROVAL 18

JAI RESEARCH FOUNDATION

APPENDIX 6 (Continued)

1. GENERAL INFORMATION

1.1 Study Director

Avani K. Solanki, M.Sc.

Deputy Study Director

Dr. Rajendra M. Nagane, M.V.Sc.

1.2 Test Facility Management

Dr. Manish V. Patel

1.3 Study Schedule

Study Initiation Date	:	August 30, 2017
Experiment Start Date	:	September 04, 2017
Experiment Completion	:	Latest by November 2017
Draft Report Submission	:	Latest by November 2017
Study Completion	:	Within two weeks from the date of receipt of comments on the final draft report from the Sponsor.

1.4 Study Plan and Amendment (if any) Distribution

a. Original copy in Archive and study Sponsor; b. Photocopy to Study Director, QAU and Residue Chemistry.

2. INTRODUCTION

2.1 Objective

The objective of this study is to evaluate the micronucleus induction potential of acetamide in rat.

2.2 Regulatory Guidelines

This study is intended for regulatory submission and will be conducted in accordance with the known requirement of international guidelines:

OECD, 2016: The Organisation for Economic Co-operation and Development (OECD) Guidelines for the Testing of Chemicals, Volume II, OECD 474, Mammalian Erythrocyte Micronucleus Test, adopted by the Council on July 29, 2016.

JAI RESEARCH FOUNDATION

APPENDIX 6 (Continued)

2.3 Principle of the Test Method

The mammalian micronucleus test is used to detect cytogenetic damage (which results in a chromosomal break, fragment or lagging whole chromosome) caused by test item. The damaged chromosomal fragments remain in the anucleated cytoplasm of the erythrocyte and are visible, when stained, as a small round or oblong structure called a micronucleus during the maturation of erythrocytes. An increase in the frequency of micronucleated polychromatic erythrocytes in treated animals is an indication of induced chromosome damage.

2.4 Test Item

The Test Item Data Sheet has been completed by the Sponsor. The representative sample of acetamide will be retained for Archiving. Any residual test item will be disposed of at JRF after the expiry date unless otherwise instructed by the Sponsor. The test item procured from Tokyo Chemical Industry Co. Ltd on behalf of study sponsor. The details provided by the supplier are as below:

Test Item Name	Acetamide
IUPAC Name	Acetamide
CAS Number	60-35-5
Molecular Formula	C_2H_5NO
Molecular Weight	59.07 g/mol
Molecular Structure	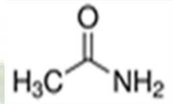
Batch/Lot Number	QYD4G
Analyzed Purity/ Concentration	99.2% (Information provided by the Supplier, Tokyo Chemical Industry Co., Ltd. (TCI) via Certificate of Analysis)
Manufactured by	Tokyo Chemical Industry Co. Ltd
Supplied to JRF by	Procured by JRF from Tokyo Chemical Industry Co. Ltd in favour of sponsor
Date of Receipt	July 29, 2017
Date of Expiry	July 28, 2019*
Appearance	White solid
Test Item Characterization under GLP	Yes, by Jai Research Foundation

APPENDIX 6 (Continued)

**Storage Condition (at JRF)**	As per the instruction received from the Supplier, TCI on storage of the test item, the test item will be stored :<br>Storage Temperature : Room temperature<br>Storage Container : In original container as supplied by the Supplier<br>Storage condition : Store in its original container in isolated, dry, cool and well-ventilated area.<br>Storage Location : Test Item Control Office, JRF
**JRF Test Item Code**	**ATM 700**

***Note**: Test item expiry date was not provided by the supplier. Hence expiry date was mentioned as per the JRF SOP No JRF/ARC/SOP-853, Issue No. Q.

Source of Molecular Weight, Molecular Formula and Molecular Structure: www.sigmaaldrich.com.

3. GOOD LABORATORY PRACTICE (GLP)

3.1 GLP Compliance

This study will be conducted in compliance with the OECD Principles of Good Laboratory Practice (as revised in 1997), ENV/MC/CHEM(98)17, N° 1, Environment Directorate, the Organisation for Economic Co-operation and Development, Paris (1998) and all subsequent OECD consensus documents.

3.2 Standard Operating Procedures (SOPs)

Unless otherwise specified all procedures mentioned in the study plan are subject to detailed Standard Operating Procedures of Jai Research Foundation.

3.3 Amendment to Study Plan

This study plan may be subjected to amendment. Amendment to study plan, whether initiated by the Sponsor or the Study Director will be generated, authorized by the Study Director and will be sent to the Sponsor for approval.

In the event that circumstances dictate immediate action, the nature of these circumstances will be communicated to the Sponsor as soon as practicable (by telephone, facsimile transmission or e-mail) and will be confirmed as soon as possible by way of formal study plan amendment.

3.4 Deviation(s)

Any deviation(s) will be documented in the study file and reported in the study report.

APPENDIX 6 (Continued)

3.5 Quality Assurance

This study plan has been verified by JRF Quality Assurance Unit (QAU) and documented (Number 94463). The QAU JRF will inspect the critical phase(s) of the study by study based inspection and/or process based inspection. The raw data, draft and final reports will be audited to ensure that the final report accurately reflects the raw data. The audit/inspection reports will be provided to the Study Director and the Test Facility Management. The date of audits/inspections and reporting of findings to the Study Director and the Test Facility Management will be incorporated in the study report.

4. ANIMAL WELFARE

The study will be undertaken in compliance with the 'Guidelines for Laboratory Animals Facility' issued by the Committee for the Purpose of Control and Supervision of Experiments on Animals (CPCSEA), India. These guidelines promote the humane care of animals used in research by providing specifications that will enhance animal well-being and experimental quality for the advancement of biological knowledge that is relevant to humans and animals.

Jai Research Foundation is committed to enhancing animal welfare and ensures that studies are designed and conducted to cause the minimum suffering or distress to animals, consistent with the scientific objectives and in accordance with Jai Research Foundation's policy on animal welfare.

Project proposal for the experimentation is subject to the approval by the Institutional Animal Ethics Committee (IAEC), Jai Research Foundation.

JRF is accredited with Association for Assessment and Accreditation of Laboratory Animal Care (AAALAC) that promotes the human treatment of animals in science.

4.1 Humane Endpoint

Moribund animals or animals obviously in pain or showing signs of severe and enduring distress shall be humanely killed. Depending on the time, since dose administration, and the circumstances of death, the bone marrow may be removed and used as part of the interpretation of the results, (at the discretion of the study director).

5. EXPERIMENTAL PROCEDURE

5.1 Initial Considerations

Test item, at doses, that causes marked pain and distress due to corrosive or severely irritant actions, will not be administered. If required, study will be terminated.

JAI RESEARCH FOUNDATION

APPENDIX 6 (Continued)

5.2 Reason for Selection of the Test System

Rat is selected as a test system because it is a readily available laboratory rodent species. It has been historically shown to be a suitable model for toxicokinetic and MNT studies. The OECD and other regulatory authorities also recommend it. The results of the study may be of value in predicting the toxicity of test item to human being.

5.3 Animals

Healthy, young adult rats (*Rattus norvegicus*) of Wistar (RccHan:WIST) strain obtained from Animal Breeding Facility (ABF), JRF will be used for the study. Female animals selected for the study will be nulliparous and non-pregnant. At the initiation of dosing, animals will be 6-10 weeks old and the body weight variation among the animals will be within ± 20% of the mean body weight for each sex. The animals will be identified with unique numbers by tattooing.

5.4 Acclimatisation

The animals, after veterinary examination for good health, will be acclimatised to the laboratory conditions for a minimum period of 5 days prior to commencement of treatment and they will be observed for clinical symptoms daily. After acclimatisation, the animals will be randomized using Censored Randomization Method (Gad S.C. and Weil C.S., 1994) using validated in-house developed software.

5.5 Housing and Animal Identification

The rat will be housed (no more than three per cage) in polypropylene rat cages provided with rice husk as the bedding material. Each day cages will be supplied with a polypropylene water bottle fitted with a stainless steel nozzle.

Individual rat will be identified with a unique number tattooed on the tail using a tattoo machine. The cages will be labeled with details of the study number, test item code, group number, sex, dose, type of study, cage number, and animal numbers. The labels used will be of different colours for different dose groups.

5.6 Animal Room Sanitation

Each day, the floor of the experimental procedure room will be swept and all worktops and the floor will be mopped with disinfectant solution.

5.7 Feed and Water

Animals will be fed *ad libitum* with standard rodent pellet feed (Teklad Certified Global 16% Protein Rodent Diet) and an unlimited supply of clean and filtered drinking water (Reverse Osmosis water filter system) in polypropylene bottles.

JAI RESEARCH FOUNDATION

APPENDIX 6 (Continued)

5.8 Environmental Conditions

The temperature of the experimental procedure room will be maintained at 22 ± 3 °C and the relative humidity between 40 and 70%. The photoperiod will be 12 h light and 12 h darkness, light hours being 06:00 – 18:00 h approximately, and air exchanges will be a minimum of 15 volumes /hour.

5.9 Selection of Vehicle

Acetamide is highly water soluble, so solubility will be first tested with distilled water first. In case of insolubility, test item will be suspended in vegetable oil or 0.5% carboxymethyl cellulose (CMC), unless otherwise recommended by sponsor. Fresh dose formulations will be prepared daily and administered within 2 hours of preparation. The concentration of the test item will be adjusted so as to permit constant dosing volume. All animals will receive a single standard volume of 10 mL/kg body weight by oral gavage administration. Vehicle control animals will receive the vehicle alone.

5.10 Dose Formulation Preparation, Sampling and Analysis

Since test item will be prepared freshly and will be used within 2 hours of preparation, stability of acetamide in the selected vehicle will not be tested separately.

Dose formulation will be prepared as per JRF/TOX/SOP-260 and JRF/TOX/SOP-266. For active ingredient concentration and homogeneity (in case of suspension) analysis, required samples will be collected from the prepared dose formulations (high, mid, and low dose) along with vehicle during the main study following the detailed procedures below. The size of samples will be determined by the study director/study person for feasibility considerations and to allow sufficient amount for analysis.

If dose formulations are solutions, required aliquots of the vehicle and all dose formulations will be collected from the middle portion. If dosing formulations are suspensions, aliquots from the top (T), middle (M), and bottom (B) of required dose formulation will be collected for homogeneity and concentration verification immediately following the preparation of the dose formulation during main study. The vehicle control will be sampled from the middle portion only.

In all cases, 2 sets of samples per dose formulation will be collected: the 1st set of aliquots of selected dose formulations will be analyzed for homogeneity (in case of suspension) and active ingredient concentration. The 2nd set of aliquots of selected dose formulation will be stored in the deep freezer (-70 ± 10 °C) at JRF as backup and will be analyzed only if needed.

JAI RESEARCH FOUNDATION

APPENDIX 6 (Continued)

Unless otherwise requested by the Sponsor, required samples will be collected from any partial retest of main study. These samples will be held at JRF as backup and only analyzed as would be required in the amendment. Any repetition of the affected portion of the study will be specified by study plan amendment. In all cases, any unused aliquots will be discarded after receiving approval for finalization of the report from the sponsor.

All analytical work will be conducted by the Department of Chemistry, JRF, under GLP compliance. The detailed method together with the sample preparation procedure will be fully documented in the study records and described in the final report. Analytical parameters used for analysis of prepared dose formulations for the active ingredient will be added through study plan amendment. All unused samples will be handled as per the relevant Standard Operating Procedures.

5.10.1 Analytical Acceptance Criteria

The acceptable specification for the concentration of acetamide in the dose formulation will be as described/mentioned below:

Solutions: 90 to 110% of nominal with <10% coefficient of variance (% CV) of each concentration.

Suspensions: 85 to 115% of nominal with <10% coefficient of variance (% CV) of each concentration.

In the event of a sample being outside the acceptable specification range, the study director will:

a) justify the acceptability of the results,
b) suggest re-analysis of the backup samples, or
c) retest the affected portion of the study.

5.11 Main Study

Five groups (comprising 6 animals/sex/group) will be used for this study. Group I will serve as the vehicle control, Group II (250 mg/kg), III (1000 mg/kg) and IV (2000 mg/kg) will be low, mid and high dose groups, respectively. Group V will be the positive control and will receive mitomycin-C (1.0 mg/kg body weight on Day 2 of treatment) in distilled water by the intraperitoneal route on a single occasion. The rectal temperature of the treated animals will be monitored during the main study using digital laboratory thermometer. The temperatures will generally be measured before dosing (Day 1), approximately 2, 5 and 24 hours after each dosing. Dose levels are selected based on sponsor's suggestions and the published data from earlier studies (Michael R. et al., 2014, Chieli et al., 1987, Mirkova, 1996 and Dybing et al., 1987).

JAI RESEARCH

APPENDIX 6 (Continued)

Mortality, severity of clinical symptoms, change in body temperature for up to 48 h after the initial dose and reduction in the immature to total (immature + mature) erythrocyte ratio will be considered for the evaluation of toxicity to bone marrow. For changes in thermal regulation, the body temperature rise by, at least, 1 °C or fall by, at least, 3 °C for five or more hours will be declared as having exceeded MTD. Body temperature changes, outside this range, have been previously reported to cause an increase in micronucleus formation in absence of chemical treatment (Asanami and Shimono, 1997; Asanami et al., 1998).

5.12 Evidence of Tissue Exposure

To demonstrate target organ exposure, plasma analysis will be performed along with main study to demonstrate the absorption of test item after oral dosing i.e. test item concentrations in the blood samples.

It may be inferred from the published literature (Zhao *et al.,* 2007; Putcha, Griffith and Feldman (1984)), that (i) acetamide fed orally to rats and mice is likely to rapidly reach the bloodstream and be transported throughout the body, (ii) at the proposed regimen of two daily back-to-back doses followed by bone marrow harvest around 24 hours after last dose, significant exposure of acetamide is expected to occur, and (iii) determination of acetamide levels in blood plasma should be a suitable method to provide evidence of exposure for the micronucleus assays.

Therefore in this study, blood samples will be withdrawn from each animal in each treatment group and vehicle control group at the time of sacrifice before bone marrow collection. Blood samples will be collected in heparinised (20 IU/mL) micro-centrifuge tubes. Blood samples will be collected from orbital plexus under very light isoflurane anesthesia. To separate out the plasma, blood samples will be centrifuged at 3000 rpm for 15 minutes at 4 °C. The plasma samples will be stored at -70 ± 10 °C until analysis. The plasma samples will be analysed for determination of test item concentration at Department of Chemistry, JRF. The details of bioanalytical method and results will be presented in the report. Bioanalytical parameters used for analysis of plasma samples will be added through study plan amendment.

JAI RESEARCH FOUNDATION

APPENDIX 6 (Continued)

5.13 Study Performance

Test item will be dissolved or suspended in a selected vehicle (Gad and Cassidy, 2006). Fresh dose formulation will be prepared, on the day of dose administration. Animals will be fasted overnight prior to dosing (feed, but not water). Animals will be dosed (10 mL/kg body weight) by oral intubation for 2 consecutive days, approximately 24 hours (± 1 hour) apart. The body weight will be recorded prior to the dosing on each day and also before sacrifice. Clinical signs will be recorded after dosing, each day, and before sacrifice. The animals will be sacrificed by CO_2 asphyxiation between 18 and 24 h following the last treatment of test item (MacGregor *et al*., 1987). Animals in the positive control group will be sacrificed by CO_2 asphyxiation approximately 24 hours after the last treatment (Krishna and Hayashi, 2000). Femur bones from the sacrificed animals will be excised and the epicondyle tips will be removed. The bone marrow content will be expelled by flushing with foetal bovine serum. The aspirated bone marrow content will be mixed using a syringe to dissociate the cells. Cell clump formation will be avoided, and the content will be centrifuged. The supernatant will be discarded. A minimum number of two slides will be prepared, per animal, with the cell pellet, fixed with methanol. Slides will be stained using 5% Giemsa (Heddle et al., 1984). In order to prevent bias in the scoring procedure, the slide numbers were masked with code numbers.

5.14 Historical control data

Jai Research Foundation (JRF) has conducted around 10 GLP studies for regulatory submission as per OECD TG 474 and established a historical control data base. JRF uses quality control methods, such as control charts to identify data variability and to show that the methodology is 'under control'. Quality control charts (QC charts) will be added in the report demonstrating the JRFs established historical positive control ranges and distribution, and a historical negative control ranges and distribution.

5.15 Microscopic Observation

Slides will be observed under a light microscope. The proportion of immature erythrocytes among the total (immature + mature), i.e., P/E ratio will be determined for each animal by counting a minimum of 500 erythrocytes. A minimum of 4500 polychromatic (immature) erythrocytes, per animal, will be scored for the incidence of micronuclei. Additional information may be obtained by scoring mature erythrocytes for micronuclei.

JAI RESEARCH FOUNDATION

5.16 Statistical Analysis

The data of percent micronucleated polychromatic erythrocytes (% MNPCE), P/E ratio and body weight for both the sexes will be subjected to normality test using Shapiro-Wilk's test and Bartlett's test to assess homogeneity of variance. The data will be analyzed by Chi-square and Fisher's exact test or Student's t-test depending on the nature of the data (Richardson, C. et al, 1989). If the data do show suitable homogeneity of variance, the data will be subjected to Analysis of Variance (ANOVA) followed by Dunnett's t-test (Gad and Weil, 1994). Depending upon the nature of data non-parametric tests will be performed if applicable. If increase in % micronucleated cells are statistically significant, then dose response will be evaluated with an appropriate trend test i.e. Chi-square trend analysis.

5.17 Assay Acceptance and Evaluation Criteria

5.17.1 Acceptance Criteria

The study will be considered valid as the following criteria are met:

i. The vehicle (or negative) controls should be in the range of historical control data.

ii. The positive controls should produce responses that are compatible with that of the historical data and should produce statistically significant responses compared with the concurrent negative control.

iii. Appropriate number of animals, doses and cells has been analysed.

iv. A minimum of three treatment groups including controls are analysed if the test item produces toxicity.

v. The highest dose should be a limit dose, maximum tolerable dose (MTD) without causing distress or death to the animal or produce toxicity to bone marrow.

vi. The PCE to total erythrocyte ratio should not be less than 20 % of the negative control.

5.17.2 Evaluation and Interpretation Criteria

After fulfilling the acceptability criteria, the test item will be considered clearly positive if:

i. At least one of the treatment groups exhibits statistically significant increase in the frequency of micronucleated polychromatic erythrocytes compared to concurrent negative control.

ii. A positive result is defined as a dose-dependent, significant increase in the incidence of micronuclei when evaluated with an appropriate trend test e.g. Chi-square trend analysis.

iii. Statistical and biological relevance will be considered in data interpretation.

iv. Any of the results falling outside the distribution of the historical negative control data i.e. Poisson based 95% control limits.

APPENDIX 6 (Continued)

The test item will be considered clearly negative, if, in all experimental conditions examined:

i. None of the treatment groups exhibits a statistically significant increase in the frequency of micronucleated immature erythrocytes compared with the concurrent negative control.
ii. There is no dose-related increase at any sampling time when evaluated by an appropriate trend test.
iii. All results are inside the distribution of the historical negative control data (e.g. Poisson-based 95% control limits), and
iv. Bone marrow exposure to the test item(s) occurred
v. There is no requirement for verification of a clear positive or clear negative response.

6. REPORT

Unless otherwise instructed by the Sponsor, one copy of the final report will be issued along with one soft copy in PDF. The report will include the following information:
Summary

Test item:

- Identification and CAS number, if known
- Physical nature and purity
- Phys-chem. properties relevant to the conduct of the study
- Stability of the test item, if known
- Source, lot number, limit date for use, if known

Vehicle:

- Justification for choice of vehicle
- Solubility of the test item in vehicle

Test Animals:

- Species and strain of animals used
- Number, age and sex of animals
- Source and housing conditions, diet, etc.
- Method for uniquely identifying animals
- Individual weight of the animals at the start of the experiment, including body weight range, mean and standard deviation for each group

Test conditions:

- Positive and negative (vehicle/solvent) control data
- Data from range-finding study, if conducted
- Rationale for dose level selection
- Details of dose preparation

JAI RESEARCH FOUNDATION

APPENDIX 6 (Continued)

- Details of the administration of the test item
- Rationale for route and duration of administration
- Methods for verifying that the test substance(s) reached the general circulation or target tissue;
- Detailed description of treatment and sampling schedules
- Method of euthanasia
- Methods of slide preparation
- Methods for measurement of toxicity
- Criteria for scoring micronucleated immature erythrocytes
- Number of cells analysed per animal
- Criteria for acceptability of the study
- Criteria for considering studies as positive, negative or equivocal

Results

- Animal conditions, prior to and throughout test period
- Signs of toxicity
- Proportion of immature erythrocytes among total erythrocytes
- Number of micronucleated immature erythrocytes, given separately for each animal
- Mean ± standard deviation of micronucleated immature erythrocytes per group
- Dose-response relationship, where possible
- Statistical analyses and method applied
- Concurrent negative and positive control data
- Historical control data ranges of P/E ratio and % MNPCE for male and female, with ranges, means, standard deviations, and 95% control limits for the distribution, as well as the time period covered and the number of data points
- Data supporting exposure of the bone marrow occurred
- Criteria for positive or negative responses that are met

Discussion of the results
Conclusion
Dose formulation analysis report
Signed study plan and study plan amendment(s) (if any)
Record of deviation(s) (if any)
References

7. ARCHIVES

On completion of the study all original raw data including any storage medium for electronically recorded data, documentation, the signed study plan, the study plan amendment, slides, the draft report, one original final report, and the representative sample of the test item will be retained in the GLP Archives at Jai Research Foundation for a period of ten years. At the end of this period, the Sponsor's instructions will be sought to either extend the archiving period or return the archived material to the Sponsor or dispose of the material.

JAI RESEARCH FOUNDATION

8. REFERENCES

Asanami, S., and Shimono, K., 1997a: Hypothermia induces micronuclei in mouse bone marrow cells. Mutation Research. 393, pp. 91–98.

Asanami, S., and Shimono, K., 1997b: High body temperature induces micronuclei in mouse bone marrow. Mutation Research. 390, pp. 79–83.

Asanami, S., Shimono, K., and Kaneda, S., 1998: Transient hypothermia induces micronuclei in mice. Mutation Research. 413, 7–14. G. Krishna and M. Hayashi, 2000: *In vivo* Rodent Micronucleus Assay: Study plan Conduct and Data Interpretation, Mut.res. 455, pp. 155-166.

Chieli, E., F. Aliboni, M. Saviozzi and G. Malvaldi 1987: Induction of micronucleated erythrocytes by primary thioamides and their metabolites in the mouse, Mutation Research 192, pp. 141-143.

Dybing et al., 1987: Studies on the Mechanism of Acetamide Hepatocarcinogenicity, Pharmacology & Toxicology, 60, pp. 9-16.

Gad, S.C. and Cassidy, C.D., 2006: Non-clinical Vehicles Use in Studies by Multiple Routes in Multiple Species, International Journal of Toxicology, 25, pp. 499 – 521

Gad, S.C. and Weil, C.S., 1994: "Statistics for Toxicologists". In: Principles and Methods of Toxicology, 3rd edition, Hayes A.W. (Ed), Raven press Ltd., New York, pp. 221-274.

Heddle, J.A., Stuart, E. and Salamone, M.F., 1984: The Bone Marrow Micronucleus Test, In: Hand Book of Mutagenicity Test Procedures. Second Edn., Kilbey B.J., M. Legator, W. Nichols, C. Ramel (Eds.) Elsevier, New York, pp. 441-457.

MacGregor, J.T., Heddle, J.A., Margolin, B.H., Ramel, C., Salamone, M.F., Tice, R.R. and Wild D., 1987: Guidelines for the Conduct of Micronucleus Assays in Mammalian Bone Marrow Erythrocytes, Mut. Res., 189, pp. 103-112.

Michael, R., Johannes, E., Ute, M., Markus, F. T., Simon, P., Heidrun Ellinger-Ziegelbauer, Andreas Z., 2014: Cross-Platform Toxicogenomics for the Prediction of Non-Genotoxic Hepatocarcinogenesis in Rat, Plos One, volume 9, Issue 5, May 2014.

Mirkova, E.T., 1996: Activities of the rodent carcinogens thioacetamide and acetamide in the mouse bone marrow micronucleus assay, Mutation Research 352, pp. 23-30.

OECD, 1998 : OECD Series on Principles of Good Laboratory Practice and Compliance Monitoring, Number 1, "OECD Principles on Good Laboratory Practice", ENV/MC/CHEM(98)17 (as revised in 1997).

JAI RESEARCH FOUNDATION

APPENDIX 6 (Continued)

OECD, 2016: The Organisation for Economic Co-operation and Development (OECD) Guidelines for the Testing of Chemicals, Volume II, OECD 474, Mammalian Erythrocyte Micronucleus Test, adopted by the Council on July 29, 2016.

Putcha, L et al., 1984: Disposition of ^{14}C-Acetohydroxamic acid and ^{14}C- Acetamide in the Rat, Drug Metabolism and Disposition, The American Society for Pharmacology and Experimental Therapeutics, Volume 12, No. 4, pp. 438-443.

Whitmire, M.L., Bryan, P., Henry, T.R., Holbrook, J., Lehman, P., Mollitor, T., Ohorodnik, S., Reed, D., and Wietgrefe, H.D., 2010: "Nonclinical dose formulation analysis method validation and sample analysis". The AAPS Journal, 12(4), pp. 628-34.

Zhao, D et al., 2007: Comparative transport efficiencies of urea analogues through urea transporter UT-B, Biochimica et Biophysica Acta 1768 (2007), ScienceDirect, pp. 1815-1821.

JAI RESEARCH FOUNDATION

APPENDIX 6 (Continued)

9. STUDY PLAN APPROVAL

We, the undersigned have read the whole study plan for, **"Micronucleus Test of Acetamide in Rat"** and confirm that the study will be performed as per this study plan.

Study Director	:	**AVANI K. SOLANKI**	AKSolanki August 30, 2017<br>Signature and Date
Test Facility Management	:	**MANISH V. PATEL**	August 30, 2017<br>Signature and Date
For Study Sponsor	:	**MICHIGAN STATE UNIVERSITY, UNITED STATES**	
**Name of the Sponsor's Representative**	:	DR. V. BRINGI	
**Signature and Date**	:	August 30, 2017	

JAI RESEARCH FOUNDATION

APPENDIX 6 (Continued)

STUDY PLAN / PROTOCOL AMENDMENT RECORD

STUDY N°	485-1-06-17728	AMENDMENT N°	1	EFFECTIVE DATE	September 22, 2017
STUDY TITLE	Micronucleus Test of Acetamide in Rat				

ORIGINAL DETAILS*	DETAILS AMENDED	REASON FOR AMENDMENT
Study Plan Page 9 of 18<br>**5.10 Dose Formulation Preparation, Sampling and Analysis**	**Addition**<br>Analytical method parameters (JRF Study N°: 228-2-14-17729)<br>Instrument : GC-MS<br>Column : Agilent VF-5MS, 0.25 mm (i.d.), 30m length, 0.25 µm film thickness<br>Carrier Gas : Helium<br>Injection Volume : 2.0 µL<br>Injection Temperature : 250 °C<br>Flow Rate : 1.2 mL/minute<br>Split Ratio : 1:8<br>Oven Temperature : 40 °C (Hold 2.0 min.) to 20.0 °C to 300 °C, (hold for 10 minutes) – Total of 25 minutes<br>Mass Spectrometry : Electron Ionization mode with 70 eV SIM Mode<br>Solvent Delay Time : 4.0 minutes<br>Quadruple Temperature : 150 °C<br>Data Acquisition : Selected Ion Monitoring (SIM) for masses 239 (Xanthyl-acetamide) and 253 (Xanthyl-Propionamide)	Addition of analytical method parameters for dose formulation analysis.
Study Plan Page 11 of 18<br>**5.12 Evidence of Tissue Exposure**	**Addition**<br>Analytical method parameters (JRF Study N°: 228-2-14-18476)<br>Instrument : GC-MS<br>Column : Agilent VF-5MS, 0.25 mm (i.d.), 30m length, 0.25 µm film thickness<br>Carrier Gas : Helium<br>Injection Volume : 2.0 µL<br>Injection Temperature : 250 °C<br>Flow Rate : 1.2 mL/minute<br>Split Ratio : 1:8<br>Oven Temperature : 40 °C (Hold 2.0 minute) to 20.0 °C to 300 °C, (hold for 10 minutes) – Total of 25 minutes<br>Mass Spectrometry : Electron Ionization mode with 70 eV<br>Data Acquisition : Selected Ion Monitoring (SIM) for masses 239 (Xanthyl-acetamide), 242 (Xanthyl-3d-acetamide) and 253 (Xanthyl-Propionamide)	Addition of analytical method parameters for plasma sample analysis.
Study Plan Page 12 of 18<br>**5.13 Study Performance**<br>Animals will be fasted overnight prior to dosing (feed, but not water).	Animals will be fasted overnight prior to day 1 of dosing (feed, but not water).	Missed to correct during study plan finalisation.

* Reference of page N°, paragraph number etc.

APPENDIX 6 (Continued)

STUDY PLAN / PROTOCOL AMENDMENT RECORD (Continued)

STUDY N°	485-1-06-17728	AMENDMENT N°	1	EFFECTIVE DATE	September 22, 2017
STUDY TITLE	Micronucleus Test of Acetamide in Rat				

REVIEWED BY (QAU)	Hemangini Patel<br>Name	Patel September 22, 2017<br>Signature & Date

AUTHORISED BY

For JRF		For SPONSOR (S)
Study Director	Asolanki September 22, 2017<br>Signature & Date:<br>Name: **Avani K. Solanki**	**MICHIGAN STATE UNIVERSITY, 220 TROWBRIGE RD, EAST LANSING MI, 48824, UNITED STATES**<br><br>Signature & Date: September 25, 2017
Facility Management	September 22, 2017<br>Signature & Date:<br>Name: **Dr. Manish V. Patel**	

The sponsor is requested to send one original, signed copy of the amendment to JRF.
Amendment Distribution: Archives (original) and photocopy to all the copy holders of study plan/protocol
JRF/GEN/F 37/6

Page 2 of 2

JAI RESEARCH FOUNDATION

APPENDIX 6 (Continued)

STUDY PLAN / PROTOCOL AMENDMENT RECORD

STUDY N°	485-1-06-17728	AMENDMENT N°	2	EFFECTIVE DATE	November 09, 2017
STUDY TITLE	Micronucleus Test of Acetamide in Rat				

ORIGINAL DETAILS*	DETAILS AMENDED	REASON FOR AMENDMENT
Study Plan Page 5 of 18<br>**2.4 Test Item**<br>Date of Expiry: July 28, 2019	Retest Date: December 03, 2017<br>**Addition**<br>Analysed Purity (Generated at JRF): 99.198% w/w	Retest date was assigned and analysed purity have been added based on results of Test item characterisation at JRF (JRF study N° 228-2-14-17729).

* Reference of page N°, paragraph number etc.

REVIEWED BY (QAU)	Smit J. Patel<br>Name	[signature] November 09, 2017<br>Signature & Date

AUTHORISED BY

For JRF		For SPONSOR (S)
Study Director	[signature] November 09, 2017<br>Signature & Date:<br>Name: **Avani K. Solanki**	**MICHIGAN STATE UNIVERSITY, 220 TROWBRIGE RD, EAST LANSING MI, 48824, UNITED STATES**<br>[signature] November 10, 2017<br>Signature & Date:
Facility Management	[signature] Nov-09, 2017<br>Signature & Date:<br>Name: **Dr. Nadeem Ahmad Khan**	

The sponsor is requested to send one original, signed copy of the amendment to JRF.
Amendment Distribution: Archives (original) and photocopy to all the copy holders of study plan/protocol
JRF/GEN/F 37/6

JAI RESEARCH FOUNDATION

APPENDIX 7: Dose Formulation Analysis and Plasma Sample Analysis Report

JAI RESEARCH FOUNDATION

November 15, 2017

Abhishek Tater
Analyst

APPENDIX 7 (Continued)

CONTENTS

PAGE N°

TITLE PAGE......60
CONTENTS......61
ABBREVIATIONS......63
SUMMARY......64
1. INTRODUCTION......67
  1.1 Dose Formulation Analysis......67
  1.2 Plasma Sample Analysis......67
2. ANALYTICAL METHOD......68
  2.1 Instrumental Parameters......68
    2.1.1 Instrument Parameters for Dose Concentration Analysis of Acetamide on GC-MS......68
    2.1.2 Instrument Parameters for Plasma Concentration Analysis of Acetamide on GC-MS......69
3. EXPERIMENTAL PROCEDURE......70
  3.1 Instruments and Equipment......70
  3.2 Solvents and Chemicals......70
  3.3 Preparation of Solutions for Dose Formulation Analysis......70
    3.3.1 Preparation of Stock Solutions......70
    3.3.2 Preparation of Internal Standard Working Solution......70
    3.3.3 Preparation of Working Solutions of Linearity......71
    3.3.4 Preparation of Solutions of Quality Controls......71
    3.3.5 Preparation of Sample Dilution......72
  3.4 Preparation of Solutions for Plasma Concentration Analysis......72
    3.4.1 Preparation of Stock Solutions......72
    3.4.2 Preparation of Internal Standard Working Solution......72
    3.4.3 Preparation of Calibration Curve Spiking Solutions......73
    3.4.4 Preparation of Spiked Matrix CC Standards......73
    3.4.5 Preparation of Quality Control Spiking Solutions......73
    3.4.6 Preparation of Spiked Matrix Quality Control Samples......74
    3.4.7 Dilution of Rat Plasma Sample......74
  3.5 Preparation of Reagent and Solution......74
    3.5.1 0.5 M Hydrochloric Acid Solution......74
    3.5.2 0.7 M KOH Solution......74
    3.5.3 5% Xanthydrol Solution......74
    3.5.4 Diluent Solution [Methanol: Milli-Q water (50:50), % v/v]......75
    3.5.5 Saturated Sodium Chloride Solution......75

JAI RESEARCH FOUNDATION

APPENDIX 7 (Continued)

CONTENTS (Continued)

PAGE N°

3.6 Sample Processing Procedure........75
3.6.1 Derivatization Procedure for Dose Concentration Analysis........75
3.6.2 Derivatization Procedure for Plasma Concentration Analysis........75
3.7 Calculation........76
3.7.1 % RSD........76
3.7.2 % Accuracy........76
3.8 Samples Run Details........77
4. RESULTS........78
4.1 System Suitability........78
4.2 Linearity........79
4.3 Dose Formulation Analysis........81
4.4 Concentration of Acetamide in Rat Plasma........82
4.5 Diluted Quality Control Samples........84
4.6 Chromatograms........85

FIGURE 1: Linearity of Acetamide for Dose Concentration Analysis........80
FIGURE 2: Linearity of Acetamide-2,2,2-D3 Reference Standard for Plasma Concentration Analysis........80

APPENDIX 1: Animal Plasma Concentration of Acetamide........94

JAI RESEARCH FOUNDATION

APPENDIX 7 (Continued)

ABBREVIATIONS

%	-	Percent
°C	-	Degree Centigrade
CC	-	Calibration Curve
µL	-	Microlitre(s)
AR	-	Analytical Reagent
C_{max}	-	Maximum observed/peak plasma concentration
Conc.	-	Concentration
DQC	-	Dilution Quality Control
g	-	Gram(s)
GC-MS	-	Gas Chromatography-Mass Spectrometer
HQC	-	High Quality Control
i.d.	-	Internal diameter
ID	-	Identification
IS	-	Internal Standard
kg	-	Kilogram
L	-	Litre(s)
LLOQ	-	Lower Limit of Quantification
LQC	-	Low Quality Control
Mg	-	Milligram(s)
min.	-	Minute
mL	-	Milliliter(s)
mm	-	Millimeter
MQC	-	Mid Quality Control
N°	-	Number
RE	-	Relative Error
RSD	-	Relative Standard Deviation
SD	-	Standard Deviation
SS	-	Spiking Solution
T_{max}	-	Time of maximum observed peak plasma concentration
ULOQ	-	Upper Limit of Quantification

JAI RESEARCH FOUNDATION

APPENDIX 7 (Continued)

SUMMARY

A. Objective

The objective of the analysis was to estimate the concentration of acetamide in dose formulation and plasma of different groups of healthy, young adult rats (*Rattus norvegicus*) of Wistar (RccHan:WIST) strain treated with acetamide by GC-MS. To demonstrate target organ exposure, plasma analysis were performed to demonstrate the absorption of test item after oral dosing as well as to demonstrate the target organ exposure i.e. test item concentration in the blood samples.

B. Dose Formulation Analysis

Dose formulation was prepared as per JRF/TOX/SOP-260 and JRF/TOX/SOP-266. For active ingredient concentration and homogeneity analysis, samples were collected from the prepared dose formulations (high, mid, and low dose) along with vehicle. Dose formulations were aliquoted from the upper (T), middle (M), and bottom (B) portion for homogeneity and concentration verification immediately following the preparation of the dose formulation during the study. The vehicle control was sampled from the middle portion only. Mean recovery (%) obtained was as per below table:

Dose level and concentration (mg/mL)	Replication	Fortification level (mg/mL)	Mean Recovery (%)	%CV
Vehicle Control G1 (0.0)	Middle	0.0	-	-
Low dose G2 (25)	MR1	25.00	107.33	0.92
	MR2			
	MR3			
Middle dose G3 (100)	MR1	100.00	106.28	1.47
	MR2			
	MR3			
High dose G4 (200)	MR1	200.00	106.03	1.12
	MR2			
	MR3			

A calibration curve of acetamide considered as reference standard concentration ranging from 1.01 to 50.59 ppm was prepared for dose formulation analysis. The coefficient of determination (r^2) was 0.99952030 (acceptance criteria: $r^2 \geq 0.98$).

JAI RESEARCH FOUNDATION

APPENDIX 7 (Continued)

C. Plasma Sample Analysis

Five groups (comprising 6 animals/sex/group) were used for this study. Group I was served as the vehicle control, Group II (250 mg/kg), III (1000 mg/kg) and IV (2000 mg/kg) were low, mid and high dose groups, respectively. Group V was of positive control and was received mitomycin-C (1.0 mg/kg body weight on Day 2 of treatment) in distilled water by the intraperitoneal route on a single occasion. Blood samples were withdrawn from each animal in each treatment group and vehicle control group at the time of sacrifice before bone marrow collection. Blood samples were collected in heparinised (20 IU/mL) micro-centrifuge tubes. Blood samples were collected from orbital plexus under very light isoflurane anesthesia. To separate out the plasma, blood samples were centrifuged at 3000 rpm for 15 minutes at 4 °C. The plasma samples were stored at -70 ± 10 °C until analysis.The experimental outline and sample details are as below:

Group N°	Dose (mg/kg b.wt.)	Animal N°		Group N°	Dose (mg/kg b.wt.)	Animal N°	
		Male	Female			Male	Female
GI	Vehicle control	1	7	GIII	1000.0	25	31
		2	8			26	32
		3	9			27	33
		4	10			28	34
		5	11			29	35
		6	12			30	36
GII	250.0	13	19	GIV	2000.0	37	43
		14	20			38	44
		15	21			39	45
		16	22			40	46
		17	23			41	47
		18	24			42	48

Calibration curve of acetamide-2-2-2-D3 reference standard concentration ranging from 0.101 to 50.633 ppm was prepared for plasma sample analysis. The coefficient of determination (r^2) was 0.99840408 (acceptance criteria: $r^2 \geq 0.98$).

JAI RESEARCH FOUNDATION

APPENDIX 7 (Continued)

The dose formulation analysis was performed following the validated method (JRF Study N° 228-2-14-17729; "Validation of Analytical Method for Determination of Acetamide Concentration, Homogeneity and Stability in Vehicle").

The plasma samples analysis was performed following the validated method (JRF Study N° 228-2-14-18476; "Validation of Bioanalytical Method for Determination of Acetamide Concentration using Acetamide-D3 as Reference Standard in Mice and Rat Plasma"). Concentrations were obtained as per below table:

**Acetamide Concentration in Rat plasma-Group I (Dose - 0.0 mg/kg)**			**Acetamide Concentration in Rat plasma-Group II (Dose - 250.0 mg/kg)**		
**Animal N°**	**Gender**	**Concentration (ppm)**	**Animal N°**	**Gender**	**Concentration (ppm)**
L1	M	1.152	L13	M	178.625
L2		1.248	L14		243.465
L3		1.376	L15		253.470
L4		1.264	L16		167.299
L5		1.960	L17		180.908
L6		1.712	L18		98.430
L7	F	1.474	L19	F	117.209
L8		1.430	L20		198.477
L9		1.792	L21		127.572
L10		1.577	L22		117.209
L11		1.452	L23		102.951
L12		1.436	L24		101.676
**Acetamide Concentration in Rat plasma-Group III (Dose - 1000.0 mg/kg)**			**Acetamide Concentration in Rat plasma-Group IV (Dose - 2000.0 mg/kg)**		
**Animal N°**	**Gender**	**Concentration (ppm)**	**Animal N°**	**Gender**	**Concentration (ppm)**
L25	M	355.688	L37	M	597.545
L26		363.074	L38		1189.993
L27		497.947	L39		820.692
L28		471.133	L40		743.475
L29		427.780	L41		534.428
L30		317.482	L42		1079.203
L31	F	358.553	L43	F	487.202
L32		253.380	L44		605.155
L33		283.260	L45		376.188
L34		257.252	L46		389.617
L35		151.475	L47		505.108
L36		276.769	L48		1072.936

APPENDIX 7 (Continued)

1. INTRODUCTION

1.1 Dose Formulation Analysis

The objective of the analysis was to estimate the concentration of acetamide in dose formulation by GC-MS for JRF Study Number: 485-1-06-17728.

The samples analysis details are provided below:

Sample analysis	Date of samples analyses
Dose formulation analysis	September 23, 2017

1.2 Plasma Sample Analysis

The objective of the analysis was to estimate the concentration of acetamide in the plasma of different groups of healthy, young adult rats (*Rattus norvegicus*) of Wistar (RccHan:WIST) strain treated with acetamide by GC-MS for JRF Study Number: 485-1-06-17728.

The samples analysis details are provided below:

Sample analysis	Date of samples analyses
Plasma sample analysis	September 29, 2017

JAI RESEARCH FOUNDATION

APPENDIX 7 (Continued)

2. ANALYTICAL METHOD

All samples were analysed by following the validated methods (“Validation of Analytical Method for Determination of Acetamide Concentration, Homogeneity and Stability in Vehicle”, JRF Study N° 228-2-14-17729, Khanvilkar T., 2017 and “Validation of Bioanalytical Method for Determination of Acetamide Concentration using Acetamide-D3 as Reference Standard in Mice and Rat Plasma”, JRF Study N° 228-2-14-18476, Khanvilkar T., 2017).

2.1 Instrumental Parameters

2.1.1 Instrument Parameters for Dose Concentration Analysis of Acetamide on GC-MS

Column	:	Agilent VF-5MS, 0.25 mm (i.d.), 30m length, 0.25 µm film thickness
Carrier Gas	:	Helium
Injection Volume (µL)	:	2.0
Injector Temperature (°C)	:	250
Flow Rate (mL/minute)	:	1.2
Oven Temperature	:	40 °C (Hold 2.0 minutes) to 20.0 °C to 300 °C, (hold for 10 minutes) - Total of 25 minutes
Mass Spectrometry	:	Electron Ionization mode with 70 eV<br>SIM Mode<br>Solvent Delay Time : 4.0 minutes<br>Quadruple Temperature : 150 °C
Data Acquisition	:	Selected Ion Monitoring (SIM) for masses<br>Xanthyl-acetamide : 239<br>Xanthyl-propionamide : 253

JAI RESEARCH FOUNDATION

APPENDIX 7 (Continued)

2.1.2 Instrument Parameters for Plasma Concentration Analysis of Acetamide on GC-MS

Column	Agilent VF-5MS, 0.25 mm (i.d.), 30m length, 0.25 µm film thickness
Carrier Gas	Helium
Injection Volume (µL)	2.0
Injector Temperature (°C)	250
Flow Rate (mL/minute)	1.2
Oven Temperature	40 °C (Hold 2.0 minutes) to 20.0 °C to 300 °C, (hold for 10 minutes) - Total of 25 minutes
Mass Spectrometry	Electron Ionization mode with 70 eV
Data Acquisition	Selected Ion Monitoring (SIM) for masses<br>Xanthyl-acetamide : 239<br>Xanthyl-3d-acetamide : 242<br>Xanthyl-propionamide : 253

Acetamide was quantified based on the response factor of xanthyl-acetamide (area of xanthyl-acetamide over the area of xanthyl-propionamide) against a calibration plot of response factor of xanthyl-3D-acetamide (area of xanthyl-3D-acetamide over the area of xanthyl-propionamide).

JAI RESEARCH FOUNDATION

APPENDIX 7 (Continued)

3. EXPERIMENTAL PROCEDURE

3.1 Instruments and Equipment

S. N°	Instrument	Model	Make/Supplier
1	GC-MS	7890B/5977AMSD	Agilent
2	Analytical balance	GR-202	Adair & dutt
3	Laboratory oven	MSI-5	Metalab
		-	Laboratory Instruments
4	Refrigerated centrifuge	Eltek MP 400R	Electrocraft (I) Pvt. Ltd.
5	Nitrogen gas evaporator	Caterpillar	Takahe analytical Instrument
6	Freezer (-80 ± 10 °C)	Forma 900 series	Thermo Scientific
7	Vortex mixer	Vibramax 110	Heidolph
8	Refrigerator	Enerji	Siemens
9	Micropipette	Physiocare concept	Eppendorf
10	Sonicator	UCB-70	Spectralab

3.2 Solvents and Chemicals

S. N°	Name	Grade	Source
1	Methanol	HPLC	J. T. Baker
2	Hydrochloric acid	AR	SDFCL
3	Xanthydrol	-	Sigma Aldrich
4	Sodium chloride	AR	SDFCL
5	Potassium hydroxide	AR	Fisher Scientific
6	Milli-Q Water	Milli Q	Millipore
7	RO water	Elix10	Millipore
8	Ethyl acetate	HPLC	Qualigens

3.3 Preparation of Solutions for Dose Formulation Analysis

3.3.1 Preparation of Stock Solutions

Preparation of Stock Solutions					
Weight (mg) of Standard	Purity (%)	Final Volume (mL)	Volume made up with	Obtained Concentration (ppm)	Identification of Standard Stock Solution
10.20	99.2	10	Methanol	1011.84	A

3.3.2 Preparation of Internal Standard Working Solution (Propionamide)

Preparation of Stock Solutions					
Weight (mg) of Standard	Purity (%)	Final Volume (mL)	Volume made up with	Obtained Concentration (ppm)	Identification of Standard Stock Solution
10.10	100	10	Methanol	1010.00	IS

JAI RESEARCH FOUNDATION

APPENDIX 7 (Continued)

3.3.3 Preparation of Working Solutions for Linearity

The stock dilutions were prepared with diluent as per the table given below from the stock solution ‘A’. These solutions were stored at 2 - 8 ºC in refrigerator.

Identification of Standard Solution (ppm)	Solution Taken (mL)	Final Volume (mL)	Volume made up to the mark with	Obtained Concentration (ppm)	Identification of Standard Working Solutions
A – (1011.84)	0.250	1	RO water	252.96	WS6
	0.125	1		126.48	WS5
	0.050	1		50.59	WS4
	0.025	1		25.30	WS3
	0.0125	1		12.65	WS2
	0.005	1		5.06	WS1
WS6 - (252.96)	0.500	2.5		50.59	S6
WS5 - (126.48)	0.500	2.5		25.30	S5
WS4 - (50.59)	0.500	2.5		10.12	S4
WS3 - (25.30)	0.500	2.5		5.06	S3
WS2 - (12.65)	0.500	2.5		2.53	S2
WS1 - (5.06)	0.500	2.5		1.01	S1

The reference standard working solutions (S1, S2, S3, S4, S5 andS6) were injected onto the GC-MS. The area ratio was plotted against concentrations (ppm). The correlation coefficient (r), slope (b) and intercept (a) were calculated.

3.3.4 Preparation of Solutions of Quality Controls

Weight (mg) of Test item	Final volume (mL)	Purity % (w/w)	Volume made up with	Obtained concentration (ppm)
25.03	1	99.20	RO water	24829.76 (Low Dose)
200.18	1			198578.56 (High Dose)

Obtained concentration (ppm)	Solution taken (mL)	Final volume (mL)	Solution taken (mL)	Final volume (mL)	Volume made up to the mark with	Dilution factor (D)	Solution ID
24829.76 (Low Dose)	0.10	10	1	10	RO water	1000	LQC
198578.56 (High Dose)	0.10	10	0.1	10		10000	HQC

JAI RESEARCH FOUNDATION

APPENDIX 7 (Continued)

3.3.5 Preparation of Sample Dilution

Dose level and conc. (mg/mL)	Replication	Solution taken (mL)	Final volume (mL)	Solution taken (mL)	Final volume (mL)	Volume made up to the mark with	Dilution factor (D)
Vehicle Control G1 (0.0)	Middle	1	-	-	-	RO water	1
Low dose G2 (25)	MR1	0.1	10	1	10		1000
	MR2						
	MR3						
Middle dose G3 (100)	MR1	0.1	10	0.1	10		10000
	MR2						
	MR3						
High dose G4 (200)	MR1	0.1	10	0.1	10		10000
	MR2						
	MR3						

3.4 Preparation of Solutions for Plasma Sample Analysis

3.4.1 Preparation of Stock Solutions

Name of standards	Purity %	Weight of standard (mg)	Capacity of volumetric flask (mL)	Volume made up with	Obtained concentration (ppm)	Reference standard stock solution identification
Acetamide-2,2,2-D_3	99.77	10.15	5	Methanol	2025.331	AD-01
		10.20			2035.308	AD-02
Acetamide	99.20	10.20	10		1011.840	A-01
		10.25			1016.800	A-02
Propionamide	100.00	10.25	10		1025.000	IS-01

3.4.2 Preparation of Internal Standard Working Solution

Identification of reference standard solution used	Solution taken (mL)	Final volume (mL)	Obtained concentration (ppm)	Identification
IS-01	0.050	10	5.125	WI-01

JAI RESEARCH FOUNDATION

APPENDIX 7 (Continued)

3.4.3 Preparation of Calibration Curve Spiking Solutions

Stock dilution with diluents (50:50, Methanol:Milli-Q water, v/v) were prepared as per below table from Acetamide-2,2,2-D3 stock solution. These solutions were stored at 2-8 ºC in refrigerator.

Stock/SS ID	Stock/SS concentration (ppm)	Stock/SS volume (mL)	Final volume made up to (mL)	SS concentration (ppm)	SS ID
AD-01	2025.331	0.005	10	1.013	STD1 SS-01
		0.010	10	2.025	STD2 SS-01
		0.020	10	4.051	STD3 SS-01
		0.070	10	14.177	STD4 SS-01
		0.245	10	49.621	STD5 SS-01
		0.850	10	172.153	STD6 SS-01
		0.250	1	506.333	STD7 SS-01

3.4.4 Preparation of Spiked Matrix CC Standards

The above prepared CC spiking solutions were spiked in the interference free blank rat plasma in order to ranged the matrix standards concentrations as per below table.

SS ID	SS concentration (ppm)	SS volume (mL)	Plasma volume (mL)	Matrix concentration (ppm)	Sample ID
STD1 SS-01	1.013	0.010	0.090	0.101	STD1
STD2 SS-01	2.025	0.010	0.090	0.203	STD2
STD3 SS-01	4.051	0.010	0.090	0.405	STD3
STD4 SS-01	14.177	0.010	0.090	1.418	STD4
STD5 SS-01	49.621	0.010	0.090	4.962	STD5
STD6 SS-01	172.153	0.010	0.090	17.215	STD6
STD7 SS-01	506.333	0.010	0.090	50.633	STD7

3.4.5 Preparation of Quality Control Spiking Solutions

Stock dilution with diluents (50:50, Methanol:Milli-Q water, v/v) were prepared as per below table from Acetamide-2,2,2-D3 stock solution for quality control samples. These solutions were stored at 2-8 ºC in refrigerator.

Stock/SS ID	Stock/SS concentration (ppm)	Stock/SS Volume (mL)	Final volume made up to (mL)	SS concentration (ppm)	SS ID
AD-02	2035.308	0.015	10	3.053	LQC SS-01
		0.750	10	152.648	MQC SS-01
		0.212	1	431.485	HQC SS-01
		0.424	1	862.971	DQC100F SS-01

JAI RESEARCH FOUNDATION

APPENDIX 7 (Continued)

3.4.6 Preparation of Spiked Matrix Quality Control Samples

The above prepared QC spiking solutions were spiked in the interference free blank rat plasma in order to ranged the concentrations as per below table.

Preparation of spiking solutions					
Stock/SS ID	SS concentration (ppm)	SS Volume (mL)	Plasma volume (mL)	Final matrix concentration (ppm)	QC ID
LQC SS-01	3.053	0.010	0.090	0.305	LQC
MQC SS-01	152.648	0.010	0.090	15.265	MQC
HQC SS-01	431.485	0.010	0.090	43.149	HQC
DQC100F SS-01	862.971	0.010	0.090	86.297	DQC100F
DQC100F	86.297	0.001	0.099	0.863	DQC_100F

3.4.7 Dilution of Rat Plasma Sample

GI-L1_M to L6_M and GI-L7_F to L12_Fsamples were derivatized undilutedly. GII, GIII and GIV were diluted as per below table:

Sample ID	No of samples	Sample volume (µL)	Plasma volume (µL)	Total volume (µL)	Dilution Factor
GII-L13_M to L18_M	6	10	90	100	10
GII-L19_F to L24_F	6	10	90	100	10
GIII-L25_M to L30_M	6	10	90	100	10
GIII-L31_F to L36_F	6	10	90	100	10
GIV-L37_M to L42_M	6	1	99	100	100
GIV-L43_F to L48_F	6	1	99	100	100

3.5 Preparation of Reagent and Solution

3.5.1 0.5 M Hydrochloric Acid Solution

50 mL of methanol was transferred in to 100 mL volumetric flask. 4.125 mL of 37 % HCl solution was added in the same volumetric flask. Volume was made equal to mark with methanol. Solution was mixed well.

3.5.2 0.7 M KOH Solution

3.9 g of KOH was transferred to 100 mL reagent bottle and 50 mL of Milli-Q water was added to it. Solution was mixed well and volume was made equal to markwith Milli-Q water. Solution was stored in refrigerator until use.

3.5.3 5% Xanthydrol Solution

5 g of Xanthydrolwas transferred to 100 mL volumetric flask. 50 mL of methanol was added to it and solution was mixed well. Volume was made equal to markwith methanol. Solution was stored in refrigerator until use.

APPENDIX 7 (Continued)

3.5.4 Diluent Solution [Methanol: Milli-Q water (50:50), % v/v]

100 mL of Methanol and 100 mL of Milli-Q water were mixed in 200 mL of reagent bottle using measuring cylinder and mixed well.

3.5.5 Saturated Sodium Chloride Solution

71g of sodium chloride in 200 mL of Milli-Q water were mixed in 200 mL of reagent bottle using measuring cylinder and mixed well.

3.6 Sample Processing Procedure

3.6.1 Derivatization Procedure for Dose Concentration Analysis

1. 2.45 mL of samples was transferred to a 15 mL polypropylene centrifuge tube.
2. 50 µL of internal standard (50 ppm propionamide in methanol) solution was added.
3. 2.50 mL of 0.5 M HCl in methanol was added to each tube and sample was vortexed for 15 min.
4. All samples were centrifuged at 14000 rpm for 10 min.
5. Then 200 µl of xanthydrol (5%) solution were added and incubated in darkness at 40^0C for 1.5 h.
6. After 1.5 h, 3.0 g of sodium chloride was added to each tube.
7. All samples were neutralized by adding 2.0 mL of 0.7 M KOH.
8. 3.0 mL of ethyl acetate was added to each tube. Vortexed, sonicated and centrifuged all samples at 10,000 rpm for 5 min.
9. 1.3 mL of supernatant was transferredfrom each samples to a new ria vial.
10. Samples were placed on speedo-vap nitrogen evaporator until all of the ethyl acetate had been removed.
11. 130 µL of ethyl acetate was added to each ria vial. Sonicated, vortexed and centrifuged at 10000 rpm for 10 min.
12. 100 µL of the supernatant was carefully removed and placed in a GC-MS vial for analysis on GCMS instrument.

3.6.2 Derivatization Procedure for Plasma Concentration Analysis

1. 100 µL of plasma was transferred to eppendorf tube.
2. 10 µL of internal standard solution (5 ppm propionamide in methanol) was added to the tube (final concentration of 0.5 ppm for the internal standard).
3. Volume was made upto to 150 µL with water (this step is important specially for preparing standards).
4. 300 µL of 0.5 M HCl in methanol was added to each tube.
5. Samples were vortexed followed by storage in -80 ± 10 °C deep freezer for 1h.

JAI RESEARCH FOUNDATION

APPENDIX 7 (Continued)

6. Samples were centrifuged at 14000 rpm for 10 minutes at set temperature of 4 °C.
7. 250 µL of supernatant was transferred to RIA tube.
8. 200µL of xanthydrol (5%) solution was added and incubated in darkness at 40^{0}C for 2 h.
9. Samples were removed after 2 h from incubator and dried at 40 °C under nitrogen.
10. 800 µL of saturated solution of sodium chloride was added to dried sample and vortexed.
11. 60 µL of 1 M KOH solution was added to all RIA tubes and vortexed.
12. 1.6mL of ethyl acetate was added to each tube.
13. Samples were vortexed at 2000 rpm for 5 minutes followed by sonication for 1 minute.
14. Samples were centrifuged the samples at 14,000 rpm for 10 minutes at set temperature of 4 °C.
15. 1.3 mL of supernatant was transferred to pre-labeled RIA vial.
16. Samples were dried at 40°C under nitrogen gas until dryness.
17. Samples were reconstituted with 0.5 mL of ethyl acetate sonicated and vortexed.
18. Samples were centrifuged at 14000 rpm for 10 minutes at set temperature of 4°C.
19. Supernatant was carefully transferred in GC-MS vials for analysis on GC-MS instrument.

3.7 Calculation

The acetamide concentration in rat plasma was calculated using the following formula by analyst software version 1.6.3:

$$\text{Acetamide concentration (ppm)} = \frac{Y - a}{b} \times D$$

Where,

Y = Peak area ratio of sample

a = Intercept

b = Slope of the line

D = Dilution factor

3.7.1 % RSD

$$\% \text{ RSD} = \frac{\text{Standard deviation}}{\text{Mean content}} \times 100$$

3.7.2 % Accuracy

$$\text{Mean \% Accuracy} = \frac{\text{Mean recovered concentration (ppm)}}{\text{Nominal concentration (ppm)}} \times 100$$

APPENDIX 7 (Continued)

3.8 Samples Run Details

Sample analysis	Date of Samples Analyses	Accepted / Not Accepted	Reason for Not Accepted
Dose formulation analysis	September 23, 2017	Accepted	-
Plasma sample analysis	September 29, 2017	Accepted	-

JAI RESEARCH FOUNDATION

APPENDIX 7 (Continued)

4. RESULTS

4.1 System Suitability

TABLE-01			
**System suitability in RO water for dose formulation analysis**			
**Date**	**23/09/17**	**23/09/17**	
**Replicates**	**Area Ratio**		
1	52.6766	**Replicates**	**Bracketed System suitability**
2	51.0216		
3	50.4328	6	44.3659
4	51.0287	7	48.3041
5	53.5837	8	48.1594
**Mean**	**51.7487**	**Mean**	**46.9431**
**% SD**	**1.32**	**% SD**	**2.23**
**% RSD**	**2.55**	**% RSD**	**4.75**
**System suitability in rat plasma for plasma concentration analysis**			
**Date**	**29/09/17**		
**Replicates**	**Area Ratio**		
1	2.5735		
2	2.5498		
3	2.5485		
4	2.5776		
5	2.5822		
**Mean**	**2.5663**		
**% SD**	**0.02**		
**% RSD**	**0.78**		
**S/N Ratio**	**5.0**		

APPENDIX 7 (Continued)

4.2 Linearity

TABLE-02

Linearity in RO water for dose formulation analysis on 23/09/17

Linearity standards	STD1	STD2	STD3	STD4	STD5	STD6	Slope	Intercept	Coefficient of determination
Nominal conc. (ppm)	1.01	2.53	5.06	10.12	25.30	50.59	0.652245	- 0.130854	0.99952030
Back calculated conc. (ppm)	1.16	2.93	5.26	9.44	24.95	50.86			
% Accuracy	114.85	115.81	103.95	93.28	98.62	100.53			

Linearity in rat plasma for plasma sample analysis on 25/09/17

Linearity standards	STD1	STD2	STD3	STD4	STD5	STD6	STD7	Slope	Intercept	Coefficient of determination
Nominal conc. (ppm)	0.101	0.203	0.405	1.418	4.962	17.215	50.633	0.044679	- 0.002077	0.99840408
Back calculated conc. (ppm)	0.120	0.215	0.383	1.312	4.459	16.600	51.849			
% Accuracy	118.81	105.91	94.57	92.52	89.86	96.43	102.40			

JAI RESEARC

APPENDIX 7 (Continued)

FIGURE 1: Linearity of Acetamide for Dose Formulation Analysis

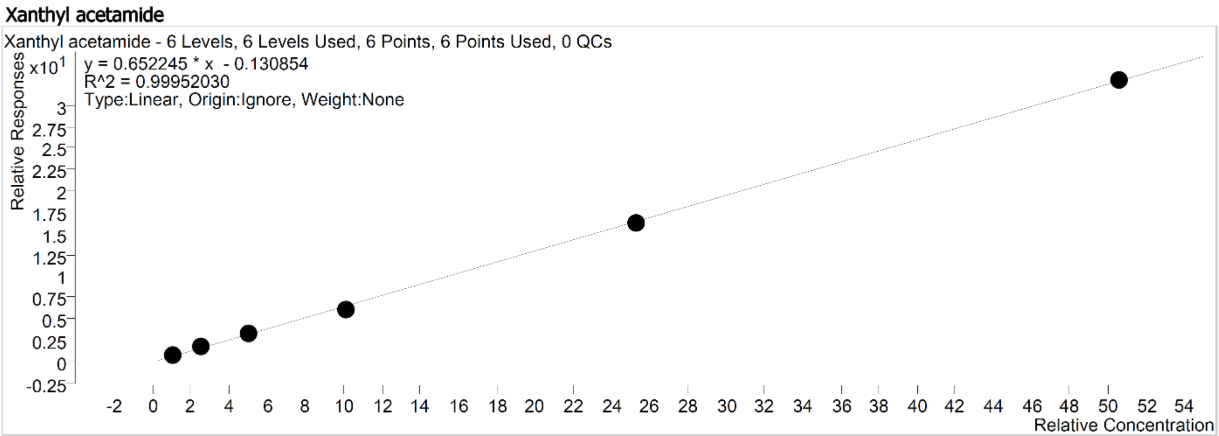

FIGURE 2: Linearity of Acetamide-2,2,2-D_3 Reference Standard for Plasma Sample Analysis

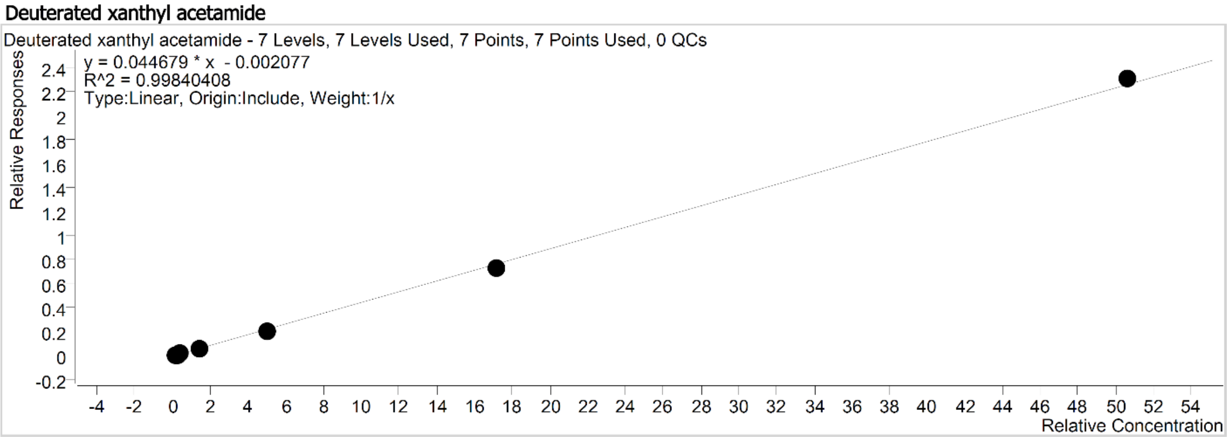

APPENDIX 7 (Continued)

4.3 Dose Formulation Analysis

TABLE-03										
Dose formulation analysis										
**Dose level and conc. (mg/mL)**	**Replication**	**Theoretical conc. based on purity of test item (ppm)**	**Dilution Factor (D)**	**Recovered conc. (ppm)**	**Analysed conc. (ppm)**	**Recovery (%)**	**Mean analysed conc. (ppm)**	**Mean Recovery (%)**	**SD**	**% CV**
Vehicle Control G1 (0.0)	Middle	0.00	-	ND	ND	-	-	-	-	-
Low dose G2 (25)	MR1	24800.00	1000	26.60	26600.00	107.26	26616.67	107.33	245.42	0.92
	MR2			26.38	26380.00	106.37				
	MR3			26.87	26870.00	108.35				
Middle dose G3 (100)	MR1	99200.00	10000	10.59	105900.00	106.75	105433.33	106.28	1553.49	1.47
	MR2			10.67	106700.00	107.56				
	MR3			10.37	103700.00	104.54				
High dose G4 (200)	MR1	198400.00	10000	20.80	208000.00	104.84	210366.67	106.03	2350.18	1.12
	MR2			21.04	210400.00	106.05				
	MR3			21.27	212700.00	107.21				
**Purity of Test Item (% w/w)**					**99.20**					

Key: ND = Not Detected, Conc. = Concentration

JAI RESEARCH FOUNDATION

APPENDIX 7 (Continued)

4.4 Concentration of Acetamide in Rat Plasma

TABLE-04							
Obtained Concentrations of Acetamide in rat plasma (ppm)							
Group	Animal N°	Gender	Dilution Factor (D)	Acetamide area response	IS area response	Response ratio	Analysed Conc. (ppm)
GI	L1	Male	1	3976	80522	0.0494	1.152
	L2		1	4422	82314	0.0537	1.248
	L3		1	5153	86693	0.0594	1.376
	L4		1	4705	86438	0.0544	1.264
	L5		1	5222	61097	0.0855	1.960
	L6		1	6141	82558	0.0744	1.712
	L7	Female	1	5499	86202	0.0638	1.474
	L8		1	5650	91348	0.0618	1.430
	L9		1	7097	91012	0.0780	1.792
	L10		1	5712	83565	0.0684	1.577
	L11		1	5931	94444	0.0628	1.452
	L12		1	6063	97647	0.0621	1.436
GII	L13	Male	10	71233	89493	0.7960	178.625
	L14		10	92085	84813	1.0857	243.465
	L15		10	101624	89900	1.1304	253.470
	L16		10	64249	86190	0.7454	167.299
	L17		10	69429	86114	0.8062	180.908
	L18		10	36095	82465	0.4377	98.430
	L19	Female	10	47152	90402	0.5216	117.209
	L20		10	72977	82485	0.8847	198.477
	L21		10	49898	87865	0.5679	127.572
	L22		10	40008	76709	0.5216	117.209
	L23		10	39643	86585	0.4579	102.951
	L24		10	40330	89178	0.4522	101.676

Key: Conc. = Concentration

JAI RESEARCH FOUNDATION

APPENDIX 7 (Continued)

TABLE-04 (Continued)

Obtained Concentrations of Acetamide in rat plasma (ppm)

Group	Animal N°	Gender	Dilution Factor (D)	Acetamide area response	IS area response	Response ratio	Analysed Conc. (ppm)
GIII	L25	Male	10	126503	79708	1.5871	355.688
	L26		10	138646	85576	1.6201	363.074
	L27		10	190750	85819	2.2227	497.947
	L28		10	178707	84982	2.1029	471.133
	L29		10	160632	84138	1.9092	427.780
	L30		10	116123	81987	1.4164	317.482
	L31	Female	10	137683	86059	1.5999	358.553
	L32		10	96875	85730	1.1300	253.380
	L33		10	110279	87279	1.2635	283.260
	L34		10	101832	88758	1.1473	257.252
	L35		10	61869	91695	0.6747	151.475
	L36		10	107346	86957	1.2345	276.769
GIV	L37	Male	100	21378	80713	0.2649	597.545
	L38		100	44805	84594	0.5296	1189.993
	L39		100	30622	83993	0.3646	820.692
	L40		100	26987	81752	0.3301	743.475
	L41		100	19926	84166	0.2367	534.428
	L42		100	40566	84501	0.4801	1079.203
	L43	Female	100	17936	83184	0.2156	487.202
	L44		100	22842	85134	0.2683	605.155
	L45		100	14948	90064	0.1660	376.188
	L46		100	14468	84110	0.1720	389.617
	L47		100	19155	85653	0.2236	505.108
	L48		100	41648	87261	0.4773	1072.936
**Intercept of Y-axis (a)**	**-0.002077**						
**Slope of the line (b)**	**0.044679**						

Key: Conc. = Concentration

JAI RESEARCH FOUNDATION

APPENDIX 7 (Continued)

4.5 Diluted Quality Control Samples

TABLE-05				
**Diluted quality control samples in rat plasma on 29/09/17**				
**Level**	**DQC100F**			
**Nominal conc. (ppm)**	86.297			
**Replicates**	**Recovered conc. (ppm)**		**% Accuracy**	**Dilution factor**
**R1**	0.7974	79.740	92.40	100.0
**R2**	0.7674	76.740	88.93	
**R3**	0.7559	75.590	87.59	
**R4**	0.7994	79.940	92.63	
**R5**	0.8083	80.830	93.66	
**R6**	0.8071	80.710	93.53	
**Mean**	**-**	**78.925**	**91.46**	**-**
**SD**	**-**	**2.21**	**-**	**-**
**% CV**	**-**	**2.80**	**-**	**-**

Key: Conc. = Concentration

JAI RESEAR

APPENDIX 7 (Continued)

4.6 Chromatograms

A. Blank for Dose Concentration Analysis

**Data File**	: 008.D
**Operator**	: PC204\abhishek.0552
**Acq Method Name**	: Acetamide
**Acquisition Date**	: 9/23/2017 7:24:49 PM
**Vial**	: 1
**Dilution**	: 1
**Sample Info**	:
**Tune File**	: ATUNE.U
**Tune Date**	:
**Quant Batch Version**	: Batch was analyzed in B.07.01, Report was generated in B.07.01
**Last Calib Update**	: 10/2/2017 10:36:55 AM

Cmpnd	Signal	RT	Limits	Response	QRatio	Limits	FinalConc
Xanthyl acetamide	239.0	13.260	12.597-13.923	0.0			0.000
Xanthyl propionamide	253.0	13.399	12.873-14.228	0.0			0.000

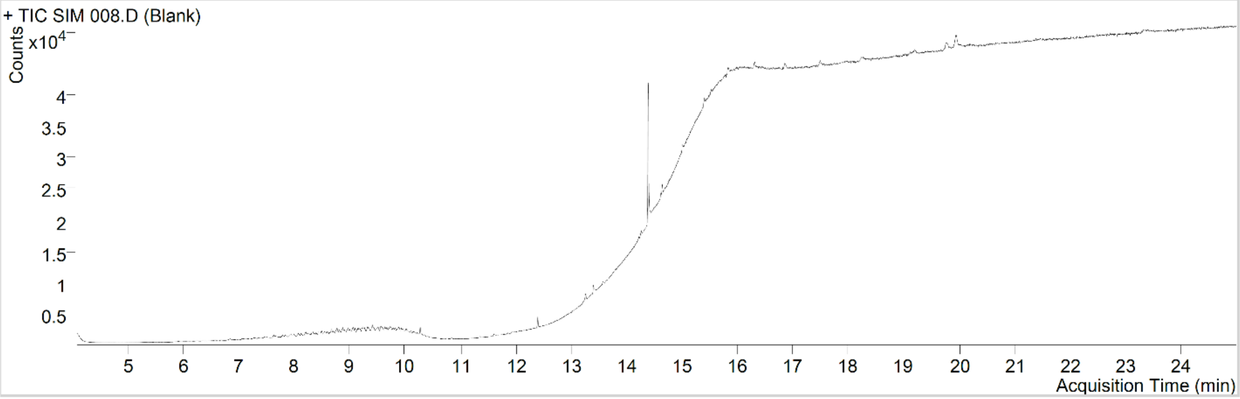

Xanthyl acetamide

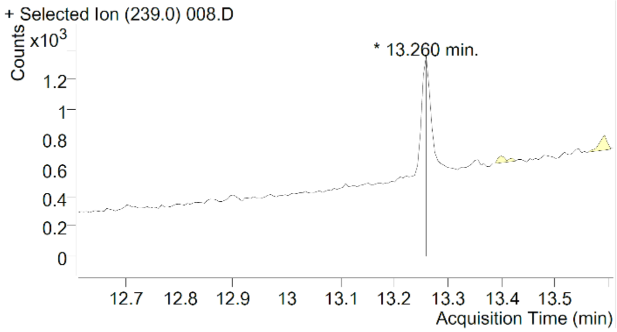

Xanthyl propionamide

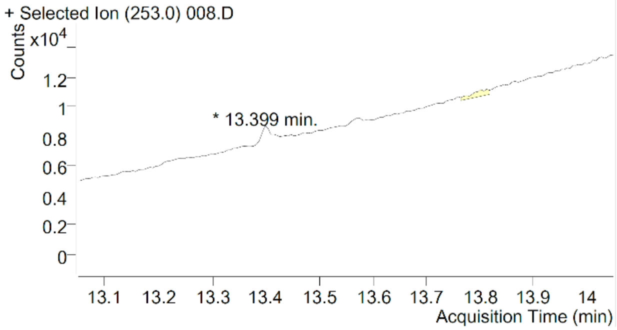

APPENDIX 7 (Continued)

4.6 Chromatograms (Continued)

B. Standard-6 for Dose Concentration Analysis

**Data File**	: 015.D
**Operator**	: PC204\abhishek.0552
**Acq Method Name**	: Acetamide
**Acquisition Date**	: 9/23/2017 11:20:56 PM
**Vial**	: 9
**Dilution**	: 1
**Sample Info**	:
**Tune File**	: ATUNE.U
**Tune Date**	:
**Quant Batch Version**	: Batch was analyzed in B.07.01, Report was generated in B.07.01
**Last Calib Update**	: 10/2/2017 10:36:55 AM

Cmpnd	Signal	RT	Limits	Response	QRatio	Limits	FinalConc
Xanthyl acetamide	239.0	13.260	12.597-13.923	1242442.6			50.856
Xanthyl propionamide	253.0	13.566	12.873-14.228	37604.3			

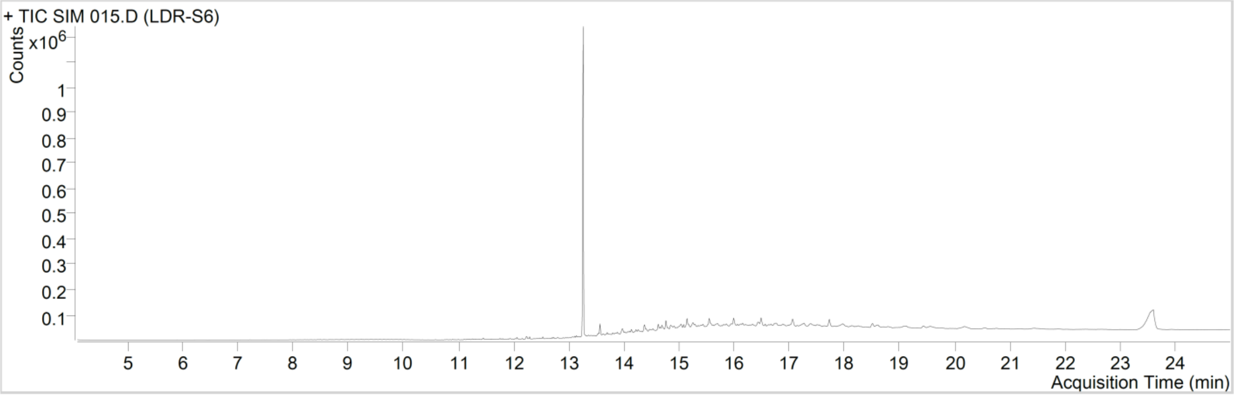

Xanthyl acetamide

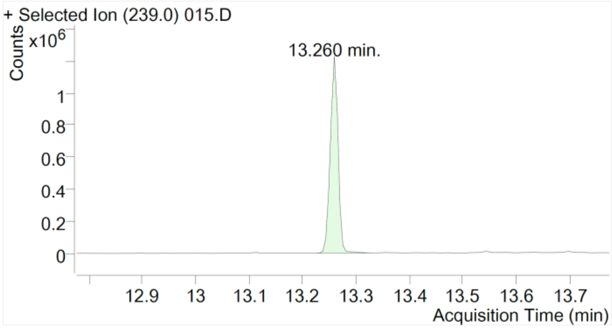

Xanthyl propionamide

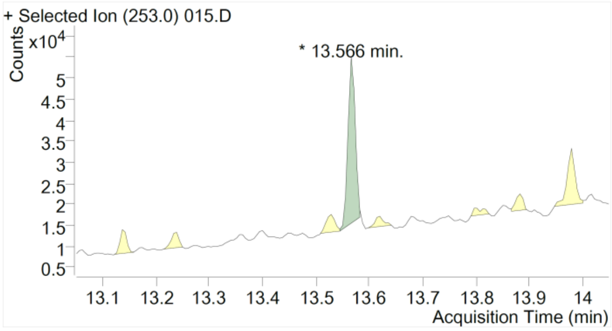

APPENDIX 7 (Continued)

4.6 Chromatograms (Continued)

C. Vehicle Control for Dose Concentration Analysis

**Data File**	: 019.D
**Operator**	: PC204\abhishek.0552
**Acq Method Name**	: Acetamide
**Acquisition Date**	: 9/24/2017 1:35:37 AM
**Vial**	: 12
**Dilution**	: 1
**Sample Info**	:
**Tune File**	: ATUNE.U
**Tune Date**	:
**Quant Batch Version**	: Batch was analyzed in B.07.01, Report was generated in B.07.01
**Last Calib Update**	: 10/2/2017 10:36:55 AM

Cmpnd	Signal	RT	Limits	Response	QRatio	Limits	FinalConc
Xanthyl acetamide	239.0		12.597-13.923				
Xanthyl propionamide	253.0		12.873-14.228				

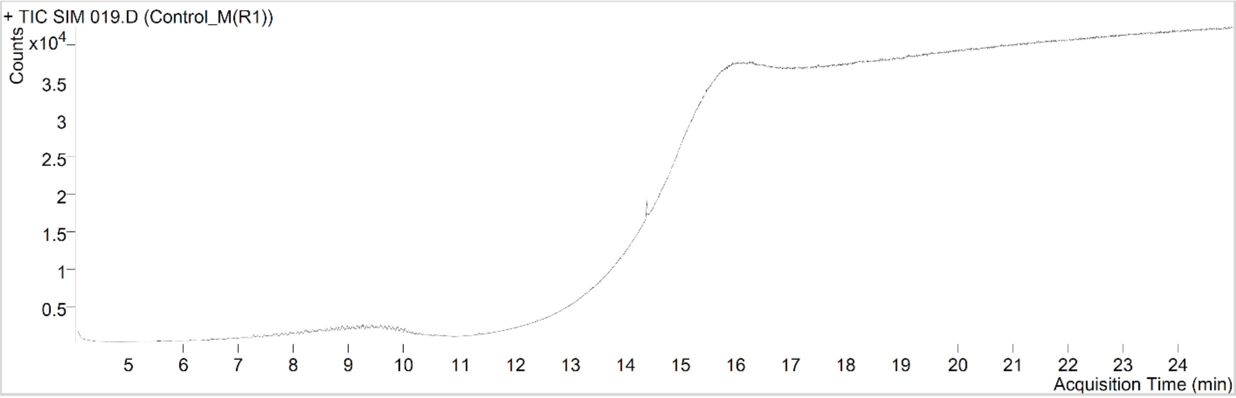

Xanthyl acetamide

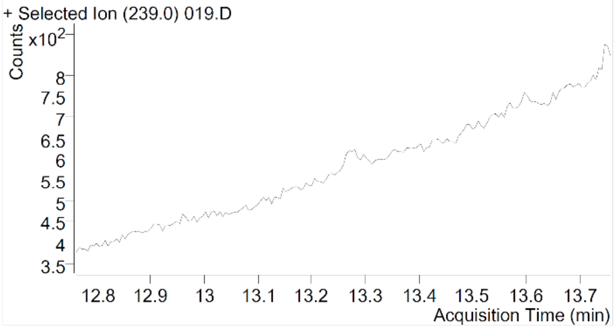

Xanthyl propionamide

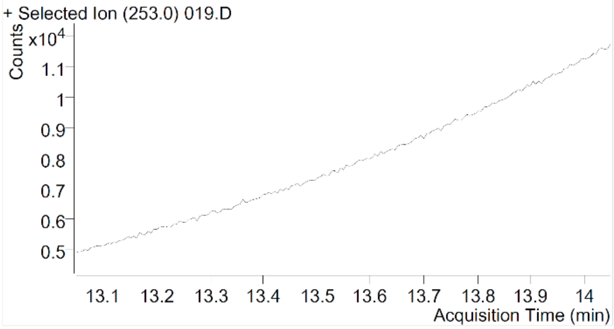

APPENDIX 7 (Continued)

4.6 Chromatograms (Continued)

D. High Dose for Dose Concentration Analysis

**Data File**	: 026.D
**Operator**	: PC204\abhishek.0552
**Acq Method Name**	: Acetamide
**Acquisition Date**	: 9/24/2017 5:31:25 AM
**Vial**	: 19
**Dilution**	: 1
**Sample Info**	:
**Tune File**	: ATUNE.U
**Tune Date**	:
**Quant Batch Version**	: Batch was analyzed in B.07.01, Report was generated in B.07.01
**Last Calib Update**	: 10/2/2017 10:36:55 AM

Cmpnd	Signal	RT	Limits	Response	QRatio	Limits	FinalConc
Xanthyl acetamide	239.0	13.254	12.597-13.923	544748.2			20.803
Xanthyl propionamide	253.0	13.565	12.873-14.228	40539.0			

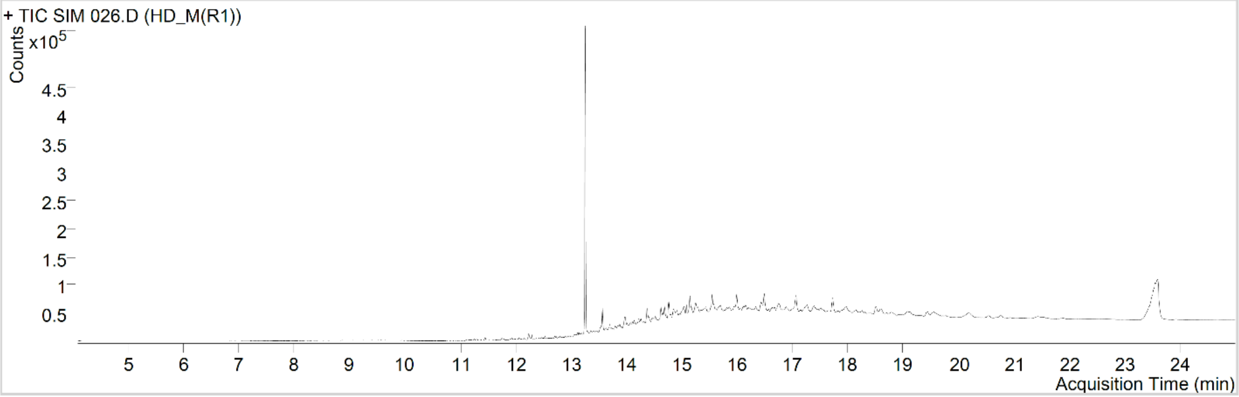

Xanthyl acetamide

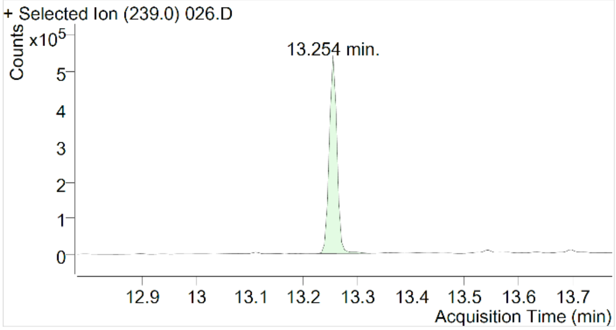

Xanthyl propionamide

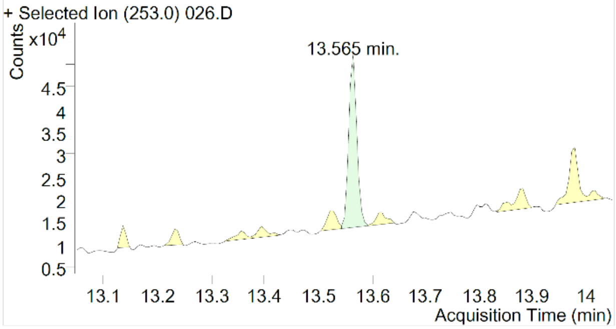

APPENDIX 7 (Continued)

4.6 Chromatograms (Continued)

E. Blank Sample of Acetamide-2,2,2-D_3 for Plasma Concentration Analysis

**Data File**	: 011.D
**Operator**	: PC204\abhishek.0552
**Acq Method Name**	: Acetamide
**Acquisition Date**	: 9/29/2017 10:46:20 PM
**Vial**	: 4
**Dilution**	: 1
**Sample Info**	:
**Tune File**	: ATUNE.U
**Tune Date**	:
**Quant Batch Version**	: Batch was analyzed in B.07.01, Report was generated in B.07.01
**Last Calib Update**	: 10/2/2017 9:48:44 AM

Cmpnd	Signal	RT	Limits	Response	QRatio	Limits	FinalConc
Deuterated xanthyl acetamide	242.0	13.163	12.581-13.905	0.0			0.000
Xanthyl propionamide	253.0	13.544	12.882-14.238	0.0			0.000

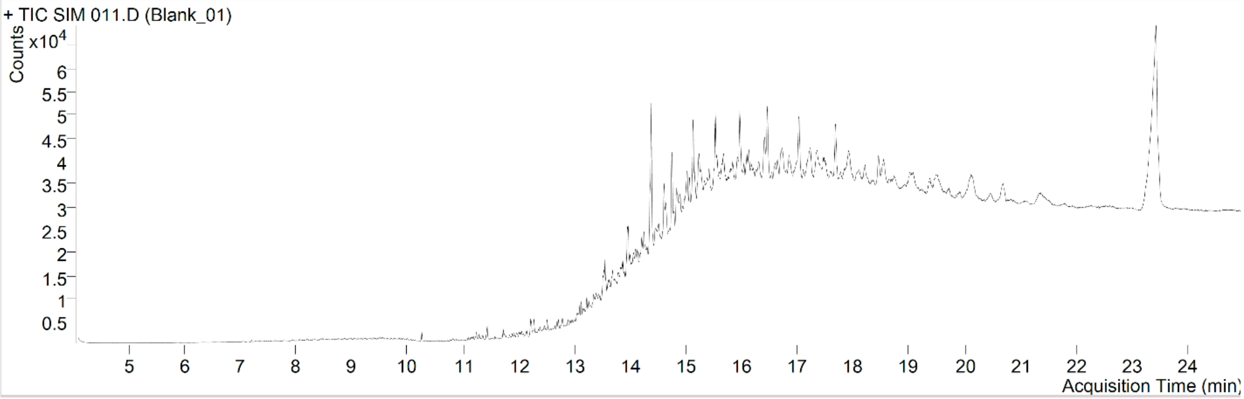

Deuterated xanthyl acetamide

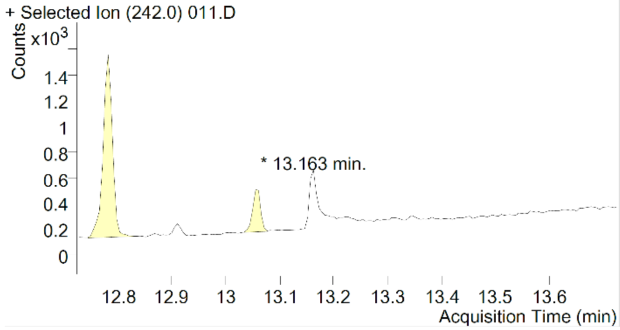

Xanthyl propionamide

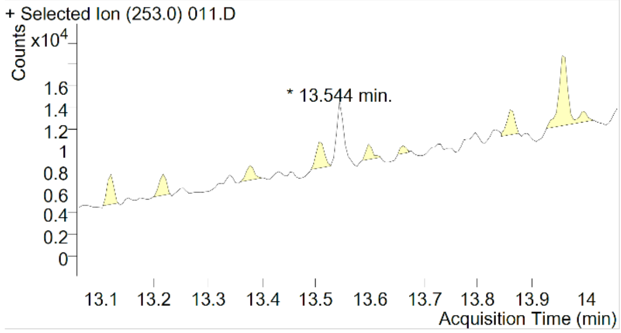

APPENDIX 7 (Continued)

4.6 Chromatograms (Continued)

F. Standard Zero Sample of Acetamide-2,2,2-D_3 for Plasma Concentration Analysis

**Data File**	: 013.D
**Operator**	: PC204\abhishek.0552
**Acq Method Name**	: Acetamide
**Acquisition Date**	: 9/29/2017 11:53:31 PM
**Vial**	: 6
**Dilution**	: 1
**Sample Info**	:
**Tune File**	: ATUNE.U
**Tune Date**	:
**Quant Batch Version**	: Batch was analyzed in B.07.01, Report was generated in B.07.01
**Last Calib Update**	: 10/2/2017 9:48:44 AM

Cmpnd	Signal	RT	Limits	Response	QRatio	Limits	FinalConc
Deuterated xanthyl acetamide	242.0	13.158	12.581-13.905	0.0			0.000
Xanthyl propionamide	253.0	13.544	12.882-14.238	91532.2			

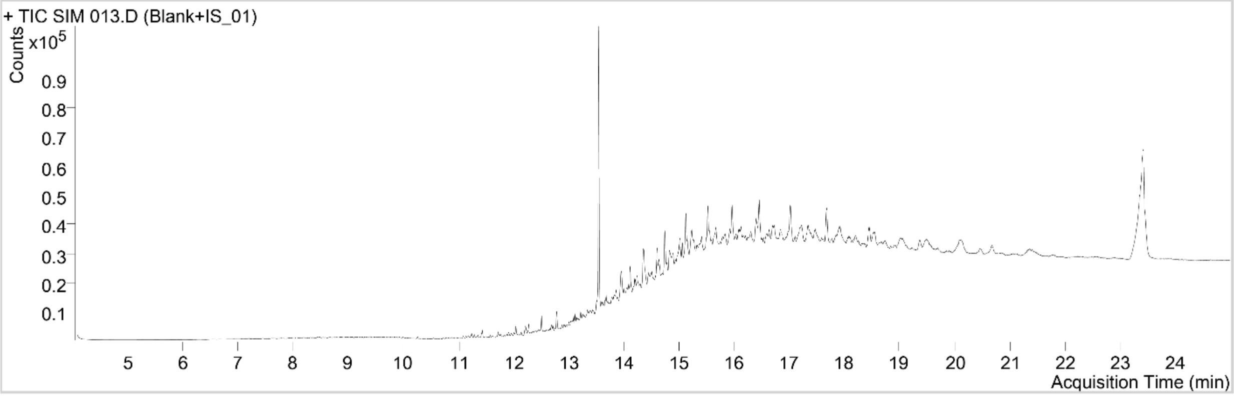

Deuterated xanthyl acetamide

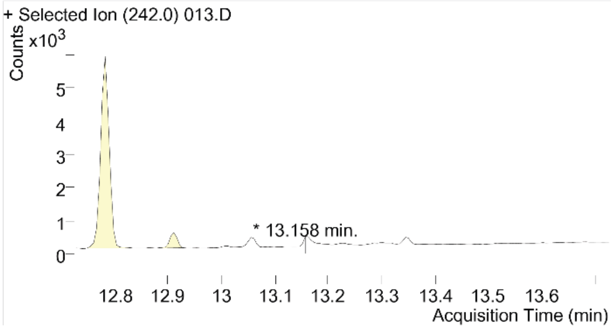

Xanthyl propionamide

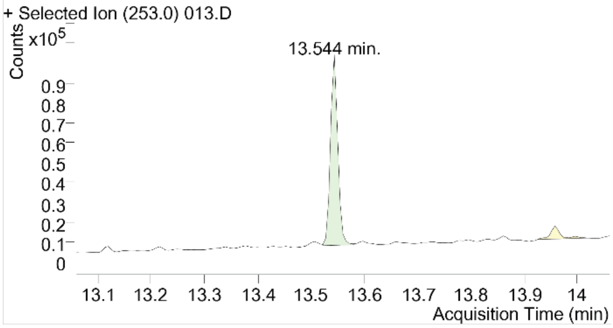

APPENDIX 7 (Continued)

4.6 Chromatograms (Continued)

G. Standard-7 sample of Acetamide-2,2,2-D_3 for Plasma Concentration Analysis

**Data File**	: 022.D
**Operator**	: PC204\abhishek.0552
**Acq Method Name**	: Acetamide
**Acquisition Date**	: 9/30/2017 4:56:26 AM
**Vial**	: 15
**Dilution**	: 1
**Sample Info**	:
**Tune File**	: ATUNE.U
**Tune Date**	:
**Quant Batch Version**	: Batch was analyzed in B.07.01, Report was generated in B.07.01
**Last Calib Update**	: 10/2/2017 9:48:44 AM

Cmpnd	Signal	RT	Limits	Response	QRatio	Limits	FinalConc
Deuterated xanthyl acetamide	242.0	13.222	12.581-13.905	239557.7			51.343
Xanthyl propionamide	253.0	13.544	12.882-14.238	103502.8			

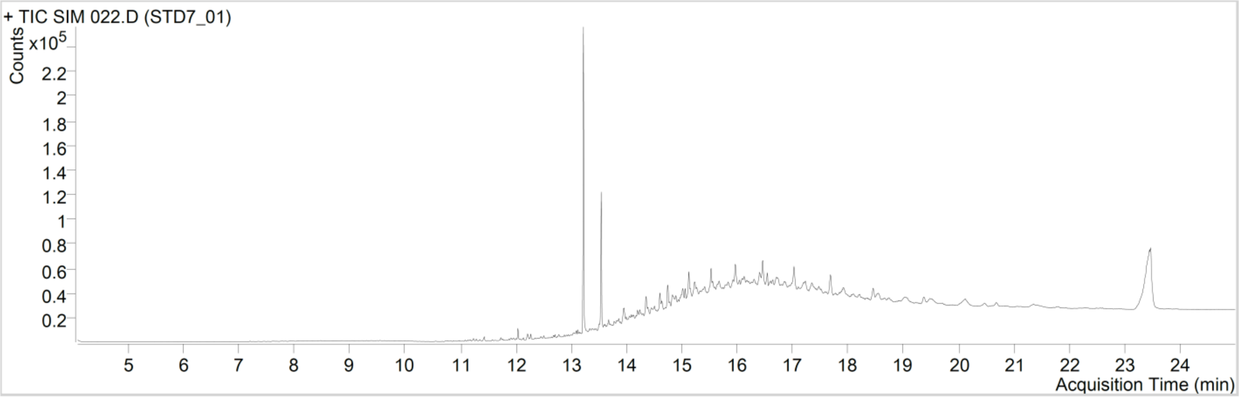

Deuterated xanthyl acetamide

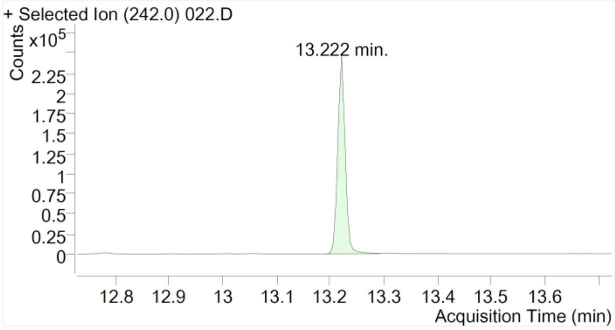

Xanthyl propionamide

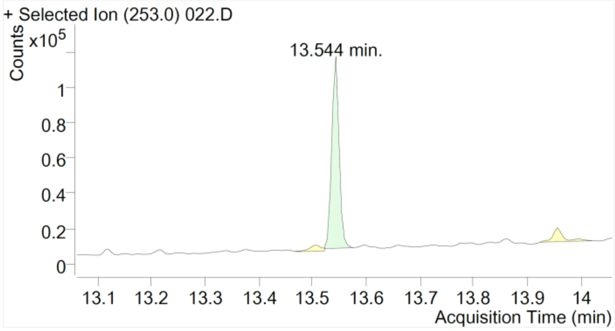

APPENDIX 7 (Continued)

4.6 Chromatograms (Continued)

H. Control sample (GI-L4_M) of Acetamide for Plasma Concentration Analysis

**Data File**	: 032.D
**Operator**	: PC204\abhishek.0552
**Acq Method Name**	: Acetamide
**Acquisition Date**	: 9/30/2017 10:32:59 AM
**Vial**	: 23
**Dilution**	: 1
**Sample Info**	:
**Tune File**	: ATUNE.U
**Tune Date**	:
**Quant Batch Version**	: Batch was analyzed in B.07.01, Report was generated in B.07.01
**Last Calib Update**	: 10/2/2017 9:48:44 AM

Cmpnd	Signal	RT	Limits	Response	QRatio	Limits	FinalConc
Xanthyl acetamide	239.0	13.233	12.581-13.905	4705.5			
Xanthyl propionamide	253.0	13.539	12.882-14.238	86438.2			

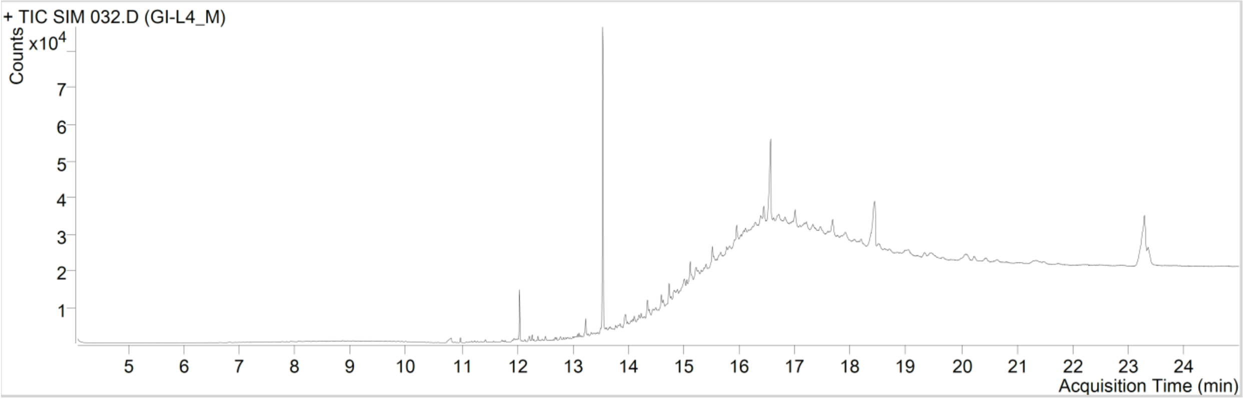

Xanthyl acetamide

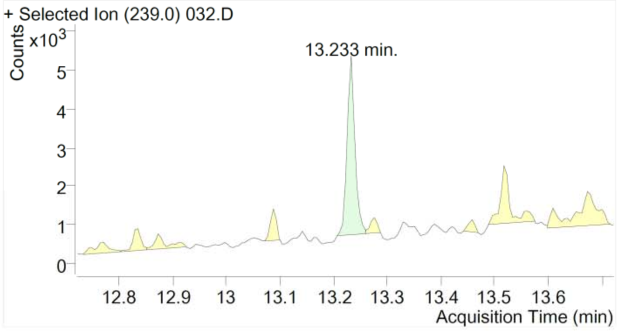

Xanthyl propionamide

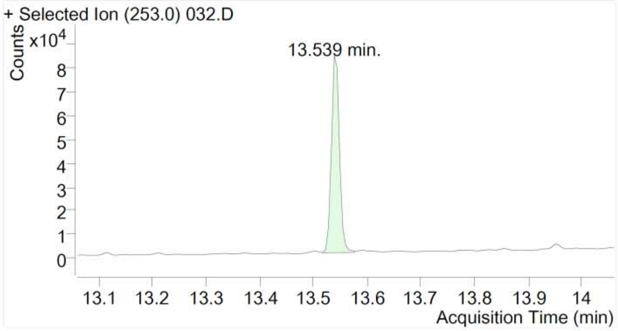

APPENDIX 7 (Continued)

4.6 Chromatograms (Continued)

I. GIV-L48_F sample of Acetamide for Plasma Concentration Analysis

Data File	: 076.D
Operator	: PC204\abhishek.0552
Acq Method Name	: Acetamide
Acquisition Date	: 10/1/2017 11:14:30 AM
Vial	: 18
Dilution	: 1
Sample Info	:
Tune File	: ATUNE.U
Tune Date	:
Quant Batch Version	: Batch was analyzed in B.07.01, Report was generated in B.07.01
Last Calib Update	: 10/2/2017 9:48:44 AM

Cmpnd	Signal	RT	Limits	Response	QRatio	Limits	FinalConc
Xanthyl acetamide	239.0	13.228	12.581-13.905	41648.1			
Xanthyl propionamide	253.0	13.539	12.882-14.238	87260.7			

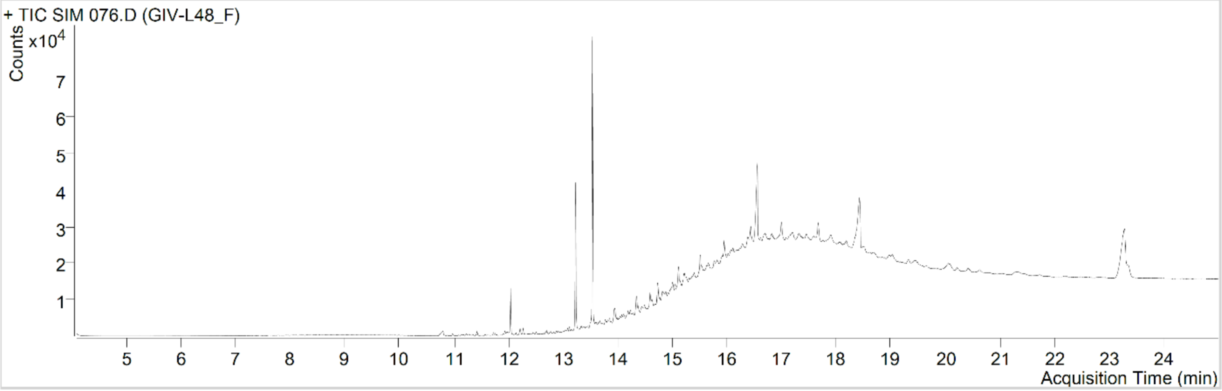

Xanthyl acetamide

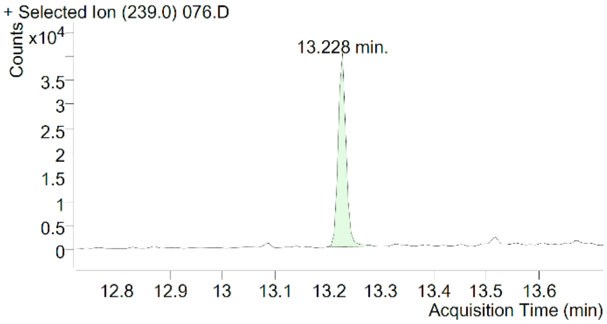

Xanthyl propionamide

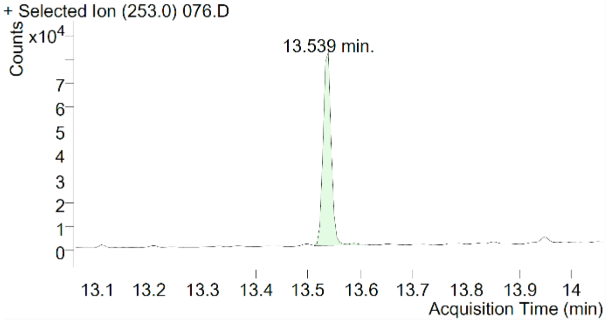

APPENDIX 7 (Continued)

APPENDIX 1: Animal Plasma Concentration of Acetamide

Acetamide Concentration in Rat plasma-Group I (Dose - 0.0 mg/kg)			Acetamide Concentration in Rat plasma-Group II (Dose - 250.0 mg/kg)		
Animal N°	Gender	Concentration (ppm)	Animal N°	Gender	Concentration (ppm)
L1	M	1.152	L13	M	178.625
L2		1.248	L14		243.465
L3		1.376	L15		253.470
L4		1.264	L16		167.299
L5		1.960	L17		180.908
L6		1.712	L18		98.430
L7	F	1.474	L19	F	117.209
L8		1.430	L20		198.477
L9		1.792	L21		127.572
L10		1.577	L22		117.209
L11		1.452	L23		102.951
L12		1.436	L24		101.676
Acetamide Concentration in Rat plasma-Group III (Dose - 1000.0 mg/kg)			Acetamide Concentration in Rat plasma-Group IV (Dose - 2000.0 mg/kg)		
Animal N°	Gender	Concentration (ppm)	Animal N°	Gender	Concentration (ppm)
L25	M	355.688	L37	M	597.545
L26		363.074	L38		1189.993
L27		497.947	L39		820.692
L28		471.133	L40		743.475
L29		427.780	L41		534.428
L30		317.482	L42		1079.203
L31	F	358.553	L43	F	487.202
L32		253.380	L44		605.155
L33		283.260	L45		376.188
L34		257.252	L46		389.617
L35		151.475	L47		505.108
L36		276.769	L48		1072.936

JAI RESEARCH FOUNDATION

Micronucleus Test of Acetamide in Rat

APPENDIX 8: Historical Control Data

Sex	Male		Female	
	P/E ratio	% MNPCE	P/E ratio	% MNPCE
**Vehicle: Aqueous Vehicle**				
Mean	0.526 (N = 8 studies)	0.028 (N = 8 studies)	0.548 (N = 5 studies)	0.035 (N = 5 studies)
Standard Deviation	0.042	0.015	0.044	0.012
**Positive control: Mitomycin-C @ 1 mg/kg body weight**				
Mean	0.529 (N = 4 studies)	1.221 (N = 4 studies)	-	-
Standard Deviation	0.037	0.198	-	-

Note: The data summarised in the table above were obtained from GLP studies conducted at JRF from January 2012 to August 2016.

Details of Positive Control used

Mitomycin-C = 1.0 mg/kg body weight

Details of Vehicle Control used

1 study with 1.0% carboxymethyl cellulose, 4 studies with distilled water and 3 studies with 0.5% carboxymethyl cellulose for Male. 1 study with 1.0% carboxymethyl cellulose, 3 studies with distilled water and 1 study with 0.5% carboxymethyl cellulose for Female.

Keys: % MNPCE = %Micronucleated Polychromatic Erythrocytes

P/E = Total Polychromatic Erythrocytes/Total Erythrocyte

JAI RESEARCH FOUNDATION

APPENDIX 8 (Continued)

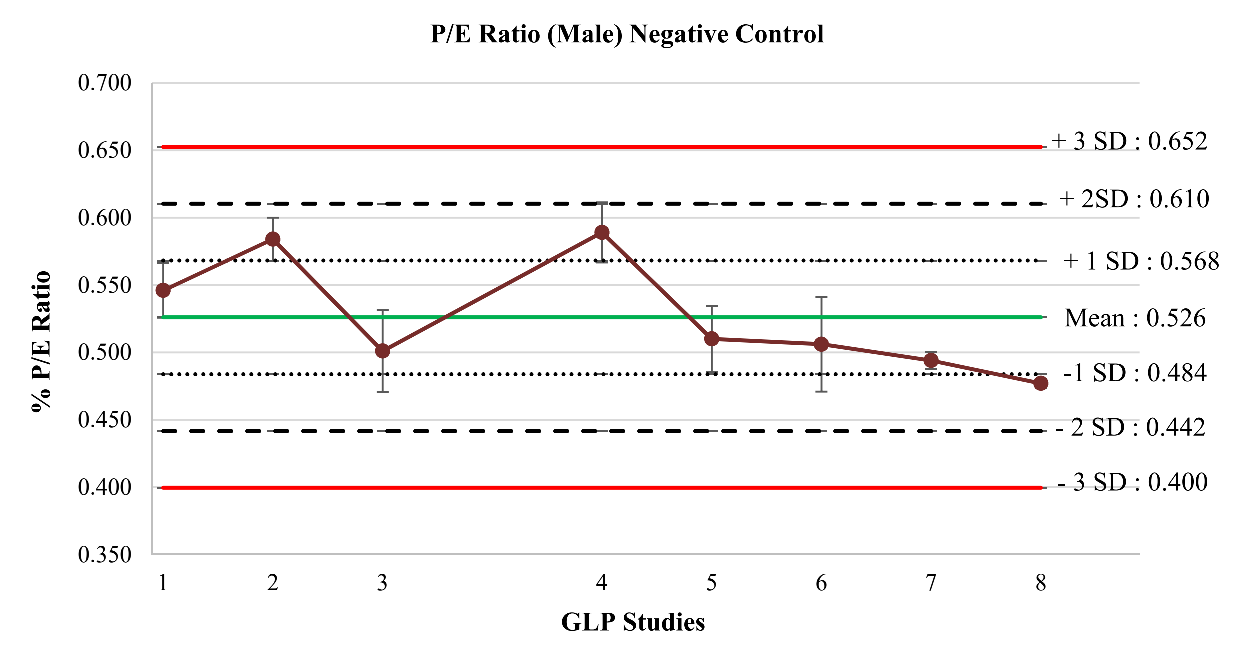

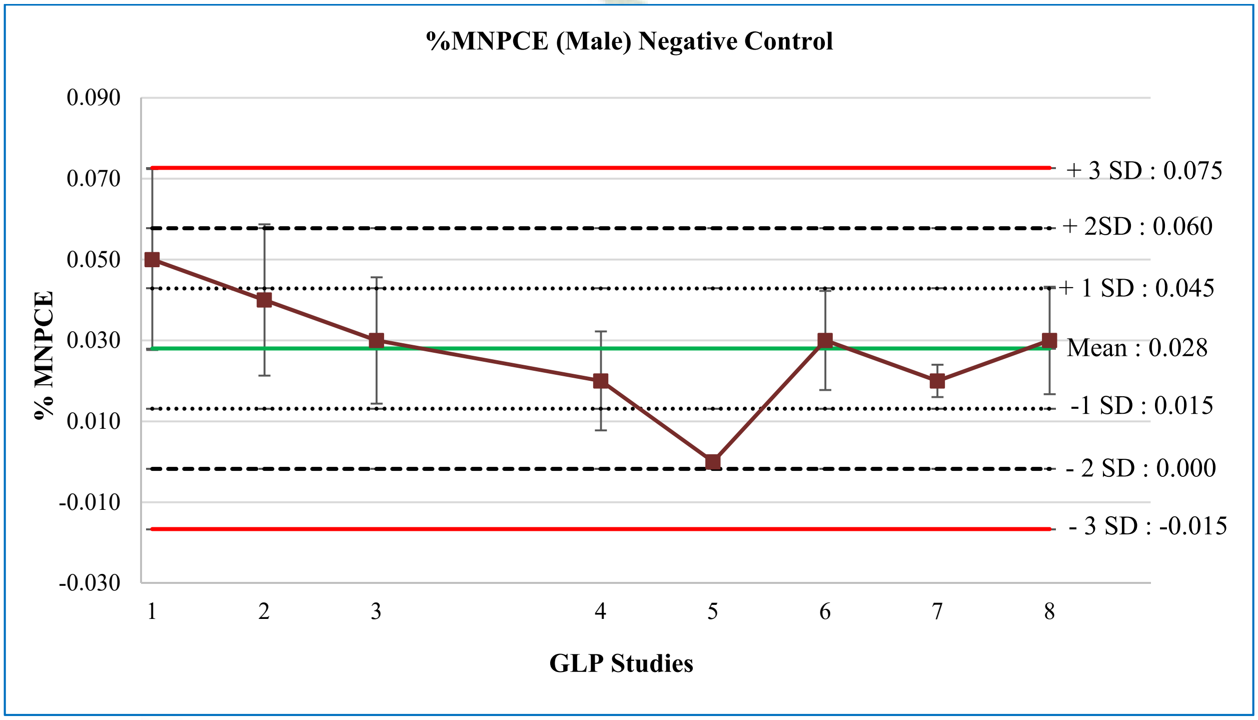

APPENDIX 8 (Continued)

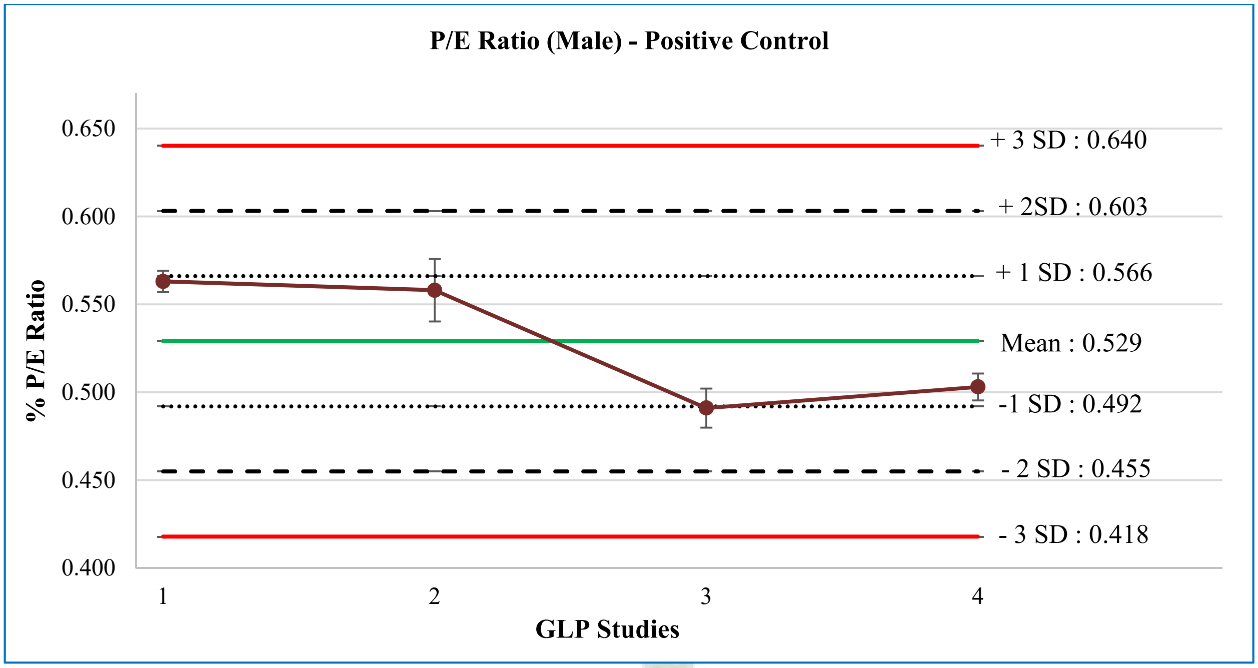

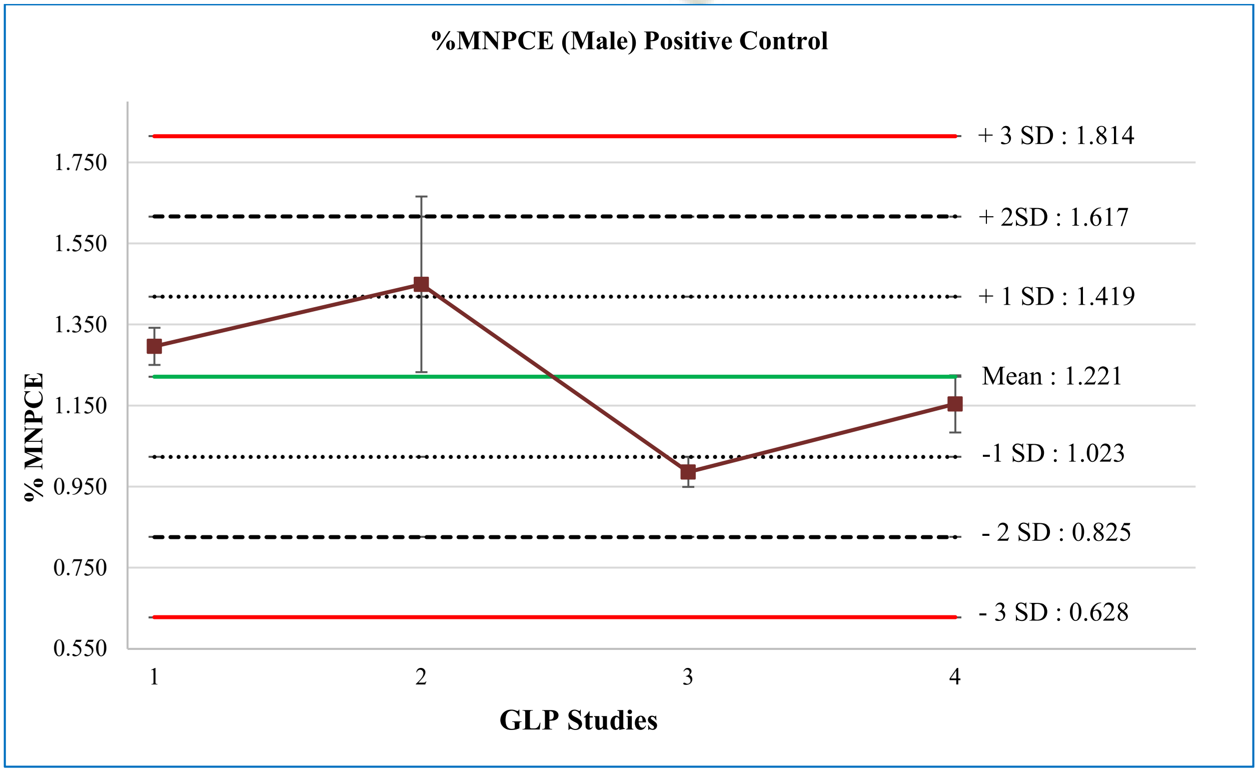

APPENDIX 8 (Continued)

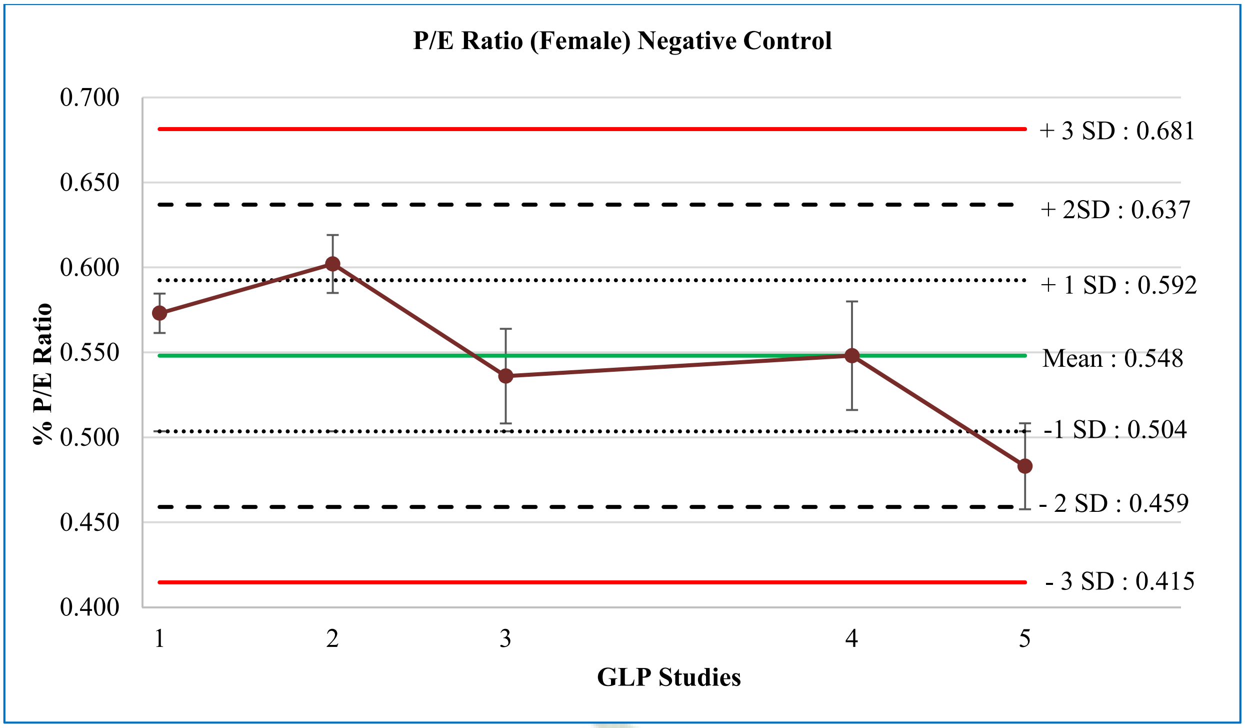

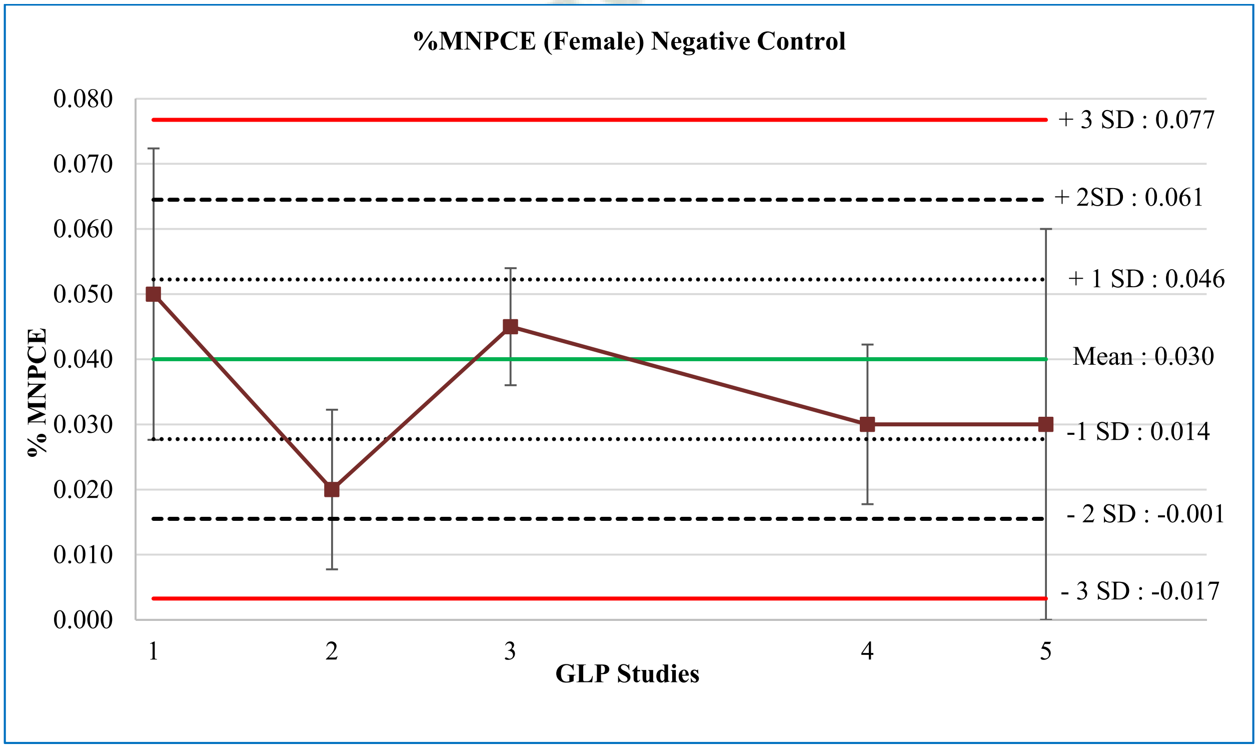

APPENDIX 8 (Continued)

Data from Historical Control Experiments					
Male – Negative Control			Female – Negative Control		
Vehicle	P/E Ratio	%MNPCE	Vehicle	P/E Ratio	%MNPCE
1% CMC	0.602	0	1% CMC	0.538	0
	0.512	0.05		0.574	0.05
	0.506	0.1		0.584	0
	0.521	0		0.608	0.1
	0.587	0.1		0.562	0.1
Distilled Water	0.58	0.05	Distilled Water	0.614	0
	0.528	0		0.557	0
	0.604	0.05		0.658	0.05
	0.623	0.1		0.579	0
	0.585	0		0.601	0.05
R.O. Water	0.437	0.1	R.O. Water	0.583	0.05
	0.519	0		0.584	0.1
	0.586	0		0.383	0
	0.465	0		0.600	0.05
	0.582	0.05		0.451	0.05
	0.451	0.05		0.577	0
	0.474	0.05		0.527	0.05
	0.565	0		0.578	0.05
	0.388	0		0.424	0.05
	0.544	0.05		0.657	0.05
Distilled Water	0.507	0	Distilled Water	0.515	0
	0.598	0.05		0.651	0
	0.585	0.05		0.541	0.05
	0.618	0		0.459	0.05
	0.637	0		0.575	0.05
Distilled Water	0.507	0	0.5% CMC	0.424	0
	0.459	0		0.46	0
	0.464	0		0.554	0
	0.532	0		0.531	0
	0.593	0		0.445	0.15
0.5% CMC	0.442	0.05			
	0.410	0.05			
	0.584	0			
	0.577	0			
	0.517	0.05			
0.5% CMC	0.488	0.02			
	0.498	0.02			
	0.517	0.02			
	0.489	0.04			
	0.480	0.02			
0.5% CMC	0.488	0.04			
	0.474	0.02			
	0.467	0.07			
	0.477	0			
	0.477	0			

Keys: P/E = Polychromatic Erythrocytes corresponding to Normochromatic Erythrocytes/Total Erythrocytes, MNPCE = MNPCE = Micronucleated Polychromatic Erythrocytes, CMC = Carboxy methyl cellulose.

JAI RESEARCH FOUNDATION

APPENDIX 8 (Continued)

Male –Positive Control		
**Vehicle**	**P/E Ratio**	**%MNPCE**
1% CMC	0.570	1.45
	0.582	1.24
	0.547	1.24
	0.555	1.35
	0.559	1.2
Distilled Water	0.598	0.95
	0.563	1.45
	0.496	1.05
	0.546	1.65
	0.584	2.15
0.5% CMC	0.463	1
	0.520	0.91
	0.471	0.91
	0.512	1
	0.488	1.11
0.5% CMC	0.486	1
	0.532	1.22
	0.497	1.37
	0.502	1
	0.500	1.18

Keys: P/E = Polychromatic Erythrocytes corresponding to Normochromatic Erythrocytes/Total Erythrocytes, MNPCE = MNPCE = Micronucleated Polychromatic Erythrocytes, CMC = Carboxy methyl cellulose.

JAI RESEARCH FOUNDATION

APPENDIX 9: Water Analysis Reports

SGS

Test Report

Print Date : 07/04/2017

SAMPLE NOT DRAWN BY SGS INDIA PVT. LTD.

Report No : CE17-001724.001 JOE No : CE17-001724

Report Control No : CER0000142381

Sample Described by Customer as : WATER

Client Name	:	JAI RESEARCH FOUNDATION
Client Address	:	Off National Highway No.8,
	:	Near Daman ganga river bridge
City	:	valvada - Vapi
Postal Code	:	396195
State	:	Gujarat
Country	:	India
Sample Type	:	WATER
Received	:	30/03/2017
Sample Qty. Recd.	:	3L & 1L
Marks on Sample	:	WATER-NEW BUILDING
Date	:	22.03.2017
Test Start/End Date	:	30/03/2017 - 07/04/2017

Analysis	Method	Result	Unit
Arsenic as As	APHA 3125 B	BDL(DL:0.005)	mg/L
Cadmium as Cd	APHA 3125 B	BDL(DL:0.001)	mg/L
Lead as Pb	APHA 3125 B	BDL(DL:0.005)	mg/L
Mercury as Hg	APHA 3125 B	BDL(DL:0.001)	mg/L
Aldrin	USEPA 3510C & 8081A	BDL(DL:0.01)	µg/l
Dieldrin	USEPA 3510C & 8081A	BDL(DL:0.01)	µg/l
Alpha Endosulfan	USEPA 3510C & 8081A	BDL(DL:0.01)	µg/l
Beta Endosulfan	USEPA 3510C & 8081A	BDL(DL:0.01)	µg/l
Endrin	USEPA 3510C & 8081A	BDL(DL:0.01)	µg/l
Gamma HCH (Lindane)	USEPA 3510C & 8081A	BDL(DL:0.01)	µg/l
Methyl parathion	USEPA 3510C & 8141A	BDL(DL:0.01)	µg/l
Malathion	USEPA 3510C & 8141A	BDL(DL:0.01)	µg/l
Phorate	USEPA 3510C & 8141A	BDL(DL:0.01)	µg/l
Methoxychlor	USEPA 3510C by GC/MS	BDL(DL:0.1)	µg/l

Remark : BDL: Below detection limit, DL: Detection limit

Remark :

This document is issued by the Company under its General Conditions of Service printed overleaf or available on request and accessible at http://www.sgs.com/terms_and_conditions.htm and Terms and Conditions for electronic documents www.sgs.com/terms_e-document.htm. Attention is drawn to the limitation of liability, indemnification and jurisdiction issues defined therein. Any holder of this document is advised that information contained hereon reflects the Company's findings at the time of its intervention only and within the limits of Client's instructions, if any. The Company's sole responsibility is to its Client and this document does not exonerate parties to a transaction from exercising all their rights and obligations under the transaction documents. Any unauthorized alteration, forgery or falsification of the content or appearance of this document is unlawful and offenders may be prosecuted to the fullest extent of the law.

Unless otherwise stated the results shown in this test report refer only to the sample(s) tested and such sample(s) are retained for 7 days (in case of perishable items) and 30 days for all other samples. The samples from regulatory bodies are to be retained as specified. This document cannot be reproduced except in full, without prior written approval of the Company.

For any feedback or complaint please write to us at feedback.mlgurgaon@sgs.com.

SGS India Pvt. Ltd. Multi Laboratory, 28 B/1 (SP), 28 B/2 (SP), IInd Main Road, Opposite to State Bank of India, Ambattur Industrial Estate, Chennai - 600 058, Tel: 91-44-66081600
Regd & Corp. Off : SGS House, 4B, A.S. Marg, Vikhroli (West), Mumbai-400083. Tel : (022) 25798421 to 28 Fax : (022) 25798431 to 35 www.sgs.com

APPENDIX 9 (Continued)

SGS

Test Report

Print Date : 07/04/2017

SAMPLE NOT DRAWN BY SGS INDIA PVT. LTD.

Report No : CE17-001724.001

JOE No : CE17-001724

Report Control No : CER0000142381

Per pro SGS India Private Ltd

Remark: All parameters are within acceptable limit as per the JRF/TOX/SOP-2077

14/04/2017

K_MANOKARAN
Authorized Signatory

****End of Report****

This document is issued by the Company under its General Conditions of Service printed overleaf or available on request and accessible at http://www.sgs.com/terms_and_conditions.htm and Terms and Conditions for electronic documents www.sgs.com/terms_e-document.htm. Attention is drawn to the limitation of liability, indemnification and jurisdiction issues defined therein. Any holder of this document is advised that information contained hereon reflects the Company's findings at the time of its intervention only and within the limits of Client's instructions, if any. The Company's sole responsibility is to its Client and this document does not exonerate parties to a transaction from exercising all their rights and obligations under the transaction documents. Any unauthorized alteration, forgery or falsification of the content or appearance of this document is unlawful and offenders may be prosecuted to the fullest extent of the law.

Unless otherwise stated the results shown in this test report refer only to the sample(s) tested and such sample(s) are retained for 7 days (in case of perishable items) and 30 days for all other samples. The samples from regulatory bodies are to be retained as specified. This document cannot be reproduced except in full, without prior written approval of the Company.

For any feedback or complaint please write to us at feedback.mlgurgaon@sgs.com.

SGS India Pvt. Ltd. Multi Laboratory, 28 B/1 (SP), 28 B/2 (SP), IInd Main Road, Opposite to State Bank of India, Ambattur Industrial Estate, Chennai - 600 058, Tel: 91-44-66081600
Regd & Corp. Off : SGS House, 4B, A.S. Marg, Vikhroli (West), Mumbai-400083. Tel : (022) 25798421 to 28 Fax : (022) 25798431 to 35 www.sgs.com

APPENDIX 9 (Continued)

JAI RESEARCH FOUNDATION

MICROBIOLOGICAL ANALYSIS CERTIFICATE OF WATER SAMPLE

Sample Type : **RO Water**
Sample Received From : **BMR Facility**
Identification N° : **JRF/BMF/43**
Date of Sample Collection : **20/03/2017**
Sample Analysis
Date of Initiation : **20/03/2017**
Date of Completion : **22/03/2017**
Sample Analysed At : **Microbiology Lab - JRF**

Parameters		Results Observed	Permissible Limits
Total viable organisms	Bacteria	Nil*	<20CFU/mL
	Fungus	Nil*	None/100 mL
Salmonella sp.		Absent	None/100 mL
Coliform organisms		Absent	< 10/100 mL
E.coli type I		Absent	None/100 mL

* = No colony in first dilution.

Conclusion: The results of analysis indicate that the microbial load is within the permissible limit (JRF/MIC/SOP-619).

Analysed by : Rahul G Badgha
Sign & Date : RBadgha March 22, 2017

Verified by : Dr. Rajesh Posia
Sign & Date : [signature] March 22, 2017

APPENDIX 9 (Continued)

JAI RESEARCH FOUNDATION

MICROBIOLOGICAL ANALYSIS CERTIFICATE OF WATER SAMPLE

Sample Type : **RO Water**
Sample Received From : **BMR Facility**
Identification N° : **JRF/BMF/58**
Date of Sample Collection : **20/03/2017**
Sample Analysis
Date of Initiation : **20/03/2017**
Date of Completion : **22/03/2017**
Sample Analysed At : **Microbiology Lab - JRF**

Parameters		Results Observed	Permissible Limits
Total viable organisms	Bacteria	Nil*	<20CFU/mL
	Fungus	Nil*	None/100 mL
Salmonella sp.		Absent	None/100 mL
Coliform organisms		Absent	< 10/100 mL
E.coli type I		Absent	None/100 mL

* = No colony in first dilution.

Conclusion: The results of analysis indicate that the microbial load is within the permissible limit (JRF/MIC/SOP-619).

Analysed by : Rahul G Badgha

Sign & Date : R Badgha March 22, 2017

Verified by : Dr. Rajesh Posia

Sign & Date : [signature] March 22, 2017

Micronucleus Test of Acetamide in Rat

APPENDIX 10: Feed Analysis Reports

2016SC

ENVIGO

Teklad Certified Global 16% Protein Rodent Diet (Sterilizable)

Lot Number	2016SC-031517MA
Date of Manufacture	03/15/17
Expiry Date	12/10/2017
Report Date	03/28/17

Analysis	Result (%)
**Proximate Analysis**	
Protein	15.50
Fat	3.84
Fiber	3.62
Moisture	12.19
Ash	4.99
Calcium	0.88
Phosphorus	0.72

Laboratory Diet Certification Report

The following data is a consolidation of results obtained from one or more independent testing laboratories. The actual laboratory results are available upon request.

Kurt Schafer
Quality Assurance Coordinator, Teklad Diets
Research Models and Services
Envigo RMS, Inc.

I have reviewed this document
2017.03.29
07:33:16 -05'00'

Analysis	Result	Units	Established Maximum Concentration
**Heavy Metals**			
Arsenic	0.12	ppm	1.00
Cadmium	< 0.10	ppm	0.50
Lead	< 0.20	ppm	1.50
Mercury	< 0.05	ppm	0.20
Selenium	0.28	ppm	0.50
**Mycotoxin**			
Aflatoxin B1, B2, G1, G2	< 5.00	ppb	5.00
**Chlorinated Hydrocarbons**			
Aldrin	< 0.01	ppm	0.03
Lindane	< 0.01	ppm	0.05
Chlordane	< 0.01	ppm	0.05
DDT & related substances	< 0.03	ppm	0.15
Dieldrin	< 0.02	ppm	0.03
Endrin	< 0.02	ppm	0.03
Heptachlor	< 0.01	ppm	0.03
Heptachlor Epoxide	< 0.01	ppm	0.03
Toxaphene	< 0.10	ppm	0.15
PCB's	< 0.10	ppm	0.15
a-BHC	< 0.01	ppm	0.05
b-BHC	< 0.01	ppm	0.05
d-BHC	< 0.01	ppm	0.05
Hexachlorobenzene	< 0.01	ppm	0.03
Mirex	< 0.01	ppm	0.02
Methoxychlor	< 0.05	ppm	0.50
**Organophosphates**			
Thimet	< 0.15	ppm	0.50
Diazinon	< 0.14	ppm	0.50
Disulfaton	< 0.15	ppm	0.50
Methyl Parathion	< 0.14	ppm	0.50
Malathion	< 0.14	ppm	0.50
Parathion	< 0.12	ppm	0.50
Thiodan	< 0.02	ppm	0.50
Ethion	< 0.14	ppm	0.50
Trithion	< 0.15	ppm	0.50

Teklad Global Diets is a trademark of Envigo. © Envigo 2015

April 29, 2017

APPENDIX 10 (Continued)

JAI RESEARCH FOUNDATION

MICROBIOLOGICAL ANALYSIS CERTIFICATE OF ANIMAL FEED

Name of Sample : **Teklad Certified Global 16% Protein Rodent Diet (Sterilizable)**
Sample Received From : **UV Room-BMR Facility**
Date of Sample Collection : **24/05/2017**
Batch N° : **2016SC-031517MA**
Sample Analysis
Date of Initiation : **24/05/2017**
Date of Completion : **26/05/2017**
Sample Analysed At : **Microbiology Lab - JRF**

Parameters		Results Observed	Permissible Limits
Total viable organisms	Bacteria	Nil*	20000 CFU/g
	Fungus	Nil*	200 CFU/g
Salmonella sp.		Absent	None/g
Coliform organisms		Absent	< 10/g
E.coli type I		Absent	None/g

*= Not detected in first dilution.

Conclusion: The results of analysis indicate that the microbial load is within the permissible limit (JRF/MIC/SOP/616).

Analysed by : Rahul G. Badgha
Sign & Date : R Badgha May 26, 2017

Verified by : Dr. Rajesh Posia
Sign & Date : [signature] May 27, 2017

Micronucleus Test of Acetamide in Rat

APPENDIX 11: Bedding Material Analysis Reports

Test Report

SGS

SAMPLE NOT DRAWN BY LABORATORY

Print Date : 05/04/2017
JOE No : CG17-006372

Report No : CG17-006372.001

Report Control No : CGR0000703548

Sample described by customer as : PADDY HUSK (RICE HUSK)

Customer Name	: JAI RESEARCH FOUNDATION
Customer Address	: OFF N.H-8,NEAR DAMAN GANGA BRIDGE, VAIVADA
	: VAPI, UMBERGAON
City	: VALSAD DIST
Postal Code	: 396195
State	: GUJARAT
Country	: INDIA
Sample Type	: PADDY HUSK (RICE HUSK)
Received	: 30/03/2017
Sample Qty. Recd.	: 500G
Date of Collection	: 22.03.2017
Test Start	: 30/03/2017
Test End Date	: 05/04/2017

Test/Parameter	Method	Result	Unit
Lead (as Pb)	SO-IN-MUL-TE-063	0.63	mg/kg
Arsenic (as As)	SO-IN-MUL-TE-063	0.07	mg/kg
Cadmium (as Cd)	SO-IN-MUL-TE-063	BLQ (LOQ : 0.01)	mg/kg
Mercury (as Hg)	SO-IN-MUL-TE-063	BLQ (LOQ : 0.01)	mg/kg
Alpha-endosulfan	AOAC 970.52	BLQ(LOQ : 0.01)	mg/kg
Beta-Endosulfan	AOAC 970.52	BLQ(LOQ : 0.01)	mg/kg
Aldrin	AOAC 970.52	BLQ(LOQ : 0.01)	mg/kg
Dieldrin	AOAC 970.52	BLQ(LOQ : 0.01)	mg/kg
Methoxychlor	AOAC 970.52	BLQ(LOQ : 0.01)	mg/kg
Endrin	AOAC 970.52	BLQ(LOQ : 0.01)	mg/kg
Methylparathion	AOAC 970.52	BLQ(LOQ : 0.01)	mg/kg
Gamma-HCH(Lindane)	AOAC 970.52	BLQ(LOQ : 0.01)	mg/kg
Phorate	AOAC 970.52	BLQ(LOQ : 0.01)	mg/kg
Malathion	AOAC 970.52	BLQ(LOQ : 0.01)	mg/kg

Per pro SGS India Private Ltd

M Thaneermalai
Authorized Signatory

Remarks :- All parameters are within acceptable limit as per the JRF/Tox/SOP-2077
14/04/17

****End of Report****

Page 1 of 1

This document is issued by the Company under its General Conditions of Service printed overleaf or available on request and accessible at http://www.sgsgroup.in/en-GB/Terms-and-Conditions.aspx for electronic documents www.sgs.com/terms_e-document.htm. Attention is drawn to the limitation of liability, indemnification and jurisdiction issues defined therein. Any holder of this document is advised that information contained hereon reflects the Company's findings at the time of its intervention only and within the limits of Client's instructions, if any. The Company's sole responsibility is to its Client and this document does not exonerate parties to

Unless otherwise stated the results shown in this test report refer only to the sample(s) tested and such sample(s) are retained for 7 days (in case of perishable items) and 30 days for all other samples. The samples from regulatory bodies are to be retained as specified. This document cannot be reproduced except in full, without prior written approval of the Company.

For any feedback or complaint please write to us at feedback.mlchennai@sgs.com.

SGS India Pvt. Ltd. | Multi Laboratory, 28 B/1 (SP), 28 B/2 (SP), IInd Main Road, Opposite to State Bank of India, Ambattur Industrial Estate, Chennai - 600 058, Tel: 91-44-66081600
Regd & Corp. Off : SGS House, 4B, A.S. Marg, Vikhroli (West), Mumbai-400083. Tel : (022) 25798421 to 28 Fax : (022) 25798431 to 35 www.sgs.com

APPENDIX 11 (Continued)

JAI RESEARCH
FOUNDATION

MICROBIOLOGICAL ANALYSIS CERTIFICATE OF BEDDING MATERIAL

Name of Sample : **Sterilized Rice Husk (Paddy husk)**
Sample Received From : **UV Room (BMR Facility)**
Identification N° : **BM1**
Date of Sample Collection : **15/03/2017**
Sample Analysis
Date of Initiation : **15/03/2017**
Date of Completion : **17/03/2017**
Sample Analysed at : **Microbiology Lab - JRF**

Result:

Parameter		Results Observed	Permissible Limit
1. Total Viable Count	Bacteria	None/Plate	None/Plate
	Fungus	None/Plate	None/Plate

Conclusion: -The results of analysis indicate that the microbial load is within the permissible limit as recommended in JRF/MIC/SOP-621.

Analysed by : Rahul G Badgha
Sign & Date : R Badgha March 17, 2017

Verified by : Dr. Rajesh Posia
Sign & Date : [signature] March 17, 2017

Micronucleus Test of Acetamide in Rat

APPENDIX 12: Certificate of Analysis of Acetamide (Provided by Supplier)

TCI

Certificate of Analysis

Jul 21, 2017 (JST)

TOKYO CHEMICAL INDUSTRY CO.,LTD.
4-10-1 Nihonbashi-Honcho, Chuo-ku, Tokyo 103-0023 Japan

Chemical Name: Acetamide		
Product Number: A0007<br>CAS: 60-35-5	Lot: QYD4G	

Tests	Results	Specifications
Purity(GC)	99.2 %	min. 98.0 %
Melting point	81.4 deg-C	80.0 to 84.0 deg-C
Solubility in Water	transparency	almost transparency

TCI Lot numbers are 4-5 characters in length. Characters listed after the first 4-5 characters are control numbers for internal purpose only.
The contents of the specifications are subject to change without advance notice. The specification values displayed here are the most up to date values. There may be cases where the product labels display a different specification, however, the product quality still meets the latest specification.

Customer service:

TCI Chemicals (India) Pvt. Ltd.
Tel: 044-2262 0909 / 044-2262 8878
Fax: 044-2262 8902
E-mail: Sales-IN@TCIchemicals.com

JAI RESEARCH

APPENDIX 13: Certificate of Analysis of Acetamide (Generated at JRF)

JAI RESEARCH FOUNDATION

CERTIFICATE OF ANALYSIS

This Certificate of Analysis is compiled from the exact data taken from JRF Study Number: 228-2-14-17729
The analysis was conducted in compliance with OECD Principles of Good Laboratory Practice (1998).

TEST ITEM DETAILS

**Test Item Name**	:	Acetamide
**Active Ingredient(s)**	:	Acetamide
**CAS Number**	:	60-35-5
**Molecular Weight**	:	59.07
**Molecular Formula**	:	C_2H_5NO
**Batch/Lot Number**	:	QYD4G
**Retest Date**	:	December 3, 2017
**Manufactured by**	:	Tokyo Chemical Industry Co.,Ltd.
**Sponsored by**	:	Michigan State University, United States
**Appearance**	:	White Solid
**Storage Condition (at JRF)**	:	Room Temperature

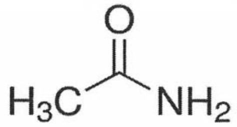

RESULT OF ANALYSIS

**Analysis Start**	:	September 04, 2017 **Analysis End** : September 04, 2017
**Method of Analysis**	:	Gas Chromatography [GC] equipped with Mass Spectrometer (GC-MS)
**Mass and Confirmation Method**	:	Not Applicable
**Analysed Purity/ Concentration**	:	99.198 % w/w

TEST FACILITY & ARCHIVES : **Jai Research Foundation, Valvada, Gujarat, India**

November 08, 2017

Signature & Date
Name: Tushar Khanvilkar
Study Director, JRF

Micronucleus Test of Acetamide in Rat

APPENDIX 14: GLP Endorsement of Compliance

सत्यमेव जयते

GOVERNMENT OF INDIA

Department of Science and Technology

National Good Laboratory Practice (GLP) Compliance Monitoring Authority (NGCMA)

Certificate of GLP Compliance

Based on the Inspection and the subsequent follow-up actions

Jai Research Foundation

Near Daman Ganga Bridge, N. H. No. 8
Valvada-396 105, Dist. Valsad (Gujarat)

is certified capable of conducting the below-mentioned tests/studies in compliance with Organization for Economic Co-operation & Development (OECD) Principles of GLP:

- **Physical-chemical Studies**
- **Toxicity Studies**
- **Mutagenicity studies**
- **Environmental Toxicity Studies on Aquatic and Terrestrial Organisms**
- **Studies on Behavior in Water, Soil and Air; Bioaccumulation Residue Studies**
- **Residue Studies**
- **Analytical and Clinical Chemistry Testing**
- **Others**

The specific areas of expertise, types of chemicals and test systems are listed in annexure overleaf.

Validity: August 5, 2016 – August 4, 2019

This certificate is subject to the condition that the test facility complies with the Terms & Conditions of the NGCMA's Document No. GLP-101 and OECD Principles of GLP.

Certificate No.: GLP/C-089/2016
Issue Date : 22-07-2016

भारत सरकार ★ Govt. of India
National GLP Compliance Monitoring Authority
Dept. of Science and Technology

(Anil Relia)
Head, NGCMA

APPENDIX 14 (Continued)

National GLP Compliance Monitoring Authority (NGCMA)

Annexure to Certificate of GLP Compliance No. GLP/C-089/2016

Areas of Expertise:

Physical-chemical Testing

Toxicity Studies

- Acute Toxicity
- Sub-acute Toxicity
- Chronic Toxicity
- Sub-chronic Toxicity
- Inhalation Toxicity studies
- Reproductive Studies
- Skin Sensitization Studies
- Neurotoxicity Studies
- Teratogenicity Studies
- Immunotoxicity Studies
- Endocrine Disruptor Assays
- Carcinogenicity Studies
- *In vitro* Skin Corrosion Test: Reconstructed Human Epidermis Test
- *In vitro* Skin Irritation Test
- Bovine Corneal Opacity and Permeability Test for Validation of Test

Mutagenicity Studies

- Bacterial Reverse Mutation Assay (AMES Test)
- Micronucleus Test *(In-vivo & In-vitro)*
- Chromosomal Aberration Test *(In-vivo & In-vitro)*
- Cell Gene Mutation
- Endocrine Disruptor Assay

Environmental Toxicity Studies on Aquatic & Terrestrial Organisms

- Alga Growth Inhibition Test
- Daphnia Acute Immobilization Test
- Acute Fish Toxicity
- Acute Oral and Contract Toxicity Test to Honeybee
- Acute Earthworm Toxicity Test
- Avian Acute Oral and Dietary Toxicity Study
- Earthworm and Daphnia Reproduction Toxicity Test
- Fish: Embryo Toxicity Test
- Fish: Short-term Toxicity Test on Embryo and Sac-fry Stages

Studies on Behaviour in Water, Soil and Air : Bioaccumulation
Residue Studies
Analytical and Clinical Chemistry Testing
Others

- Impurity Profile and Five Batch Analyses
- Bio-analytical and Toxicokinetics
- Drug Metabolism & Pharmacokinetics and Tissue distribution
- Safety Pharmacology

Types of Chemicals:

Industrial Chemicals, Pesticides, Pharmaceuticals, Veterinary Drugs, Cosmetics, Food Additives, and Feed Additives.

Test Systems:

Rat, Mouse, Rabbit, Guinea Pig, Dog, Fish, Algae, Daphnia, Honeybee, Earthworm, Japanese Quail, Mallard Duck, Cornea, Human Lymphocytes, CHO-K1 Cell Line, H295R Cell Line, HeLa 9903 Cell Line, J774A.1 Cell Line, Mouse Lymphoma Cell Line, BALB/3T3 Clone A31, KeratinoSens Cell Line, Human Monocyte Cell Line THP-1, Human Myeloid U937 Cells, Caco-2, Colo 205, *Salmonella typhimurium* and *Escherichia coli*

(Anil Relia)
Head, NGCMA
